# Echinacoside Inhibits Osteoclast Function by Down-Regulating PI3K/Akt/C-Fos to Alleviate Osteolysis Caused by Periprosthetic Joint Infection

**DOI:** 10.3389/fphar.2022.930053

**Published:** 2022-06-24

**Authors:** Tao Jiang, Hanwen Gu, Jian Wei

**Affiliations:** ^1^ Department of Joint Orthopedics, Affiliated Liutie Central Hospital of Guangxi Medical University, Liuzhou, China; ^2^ Department of Orthopedic Surgery, Zhongnan Hospital of Wuhan University, Wuhan, China

**Keywords:** infected osteolysis, echinacoside, osteoclast, periprosthetic joint infection, bone loss

## Abstract

Infected osteolysis as a common secondary osteoporosis is associated with excessive osteoclastogenesis and bone resorption. The inhibition of osteoclastogenesis and bone resorption have been demonstrated an effective approach in the treatment of osteolytic diseases. Echinacoside (ECH) is a natural phenylethanoid glycoside with multiple biological functions, including anti-inflammatory, antioxidant, and osteoblast differentiation promotion. However, the effects of ECH on osteoclast differentiation and bone resorption function remain unknown. *In vitro*, we investigated the effects of ECH on osteoclast differentiation and bone resorption induced by RANKL and its potential mechanisms. *In vivo*, we established a periprosthetic joint infection (PJI) rat model and demonstrated the changes of infected osteolysis and osteoclasts activities in surgical sites. ECH (20 mg/kg) was injected intraperitoneally after debridement for 4 weeks. Radiological evaluation and bone histomorphometric analysis was performed to assess the efficacy of ECH. The results showed that ECH inhibited osteoclast differentiation, F-actin belts formation, bone resorption function and osteoclast-specific gene expression by preventing NFATc1 translocation, down-regulating its expression and affecting the PI3K/Akt/c-Fos pathway *in vitro*. ECH also alleviated *in vivo* PJI-induced osteolysis and maintained bone mass by inhibiting osteoclast activity. Our study indicated that ECH attenuated RANKL-induced osteoclastogenesis and PJI-induced bone loss and was shown as a potentially effective therapeutic agent for osteoclast-related bone diseases.

## 1 Introduction

Periprosthetic joint infection (PJI) is one of the most serious and devastating complications after total joint arthroplasty (TJA) surgery, which severely affects the quality of patients’ life and brings a heavy medical burden (Cahill et al., 2008; Aggarwal et al., 2014). Anti-infective therapy is the mainstay of clinical practice in the PJI treatment because the most fundamental pathological manifestations of PJI are infection and inflammation (Romano et al., 2011; Hodges et al., 2021). However, the bone mass of patients with PJI has not received much attention, especially after revision surgery. Several studies have shown that some inflammatory diseases such as infected osteomyelitis (Dapunt et al., 2015), tuberculosis (Liu et al., 2020), and corona virus disease (Awosanya et al., 2022) promote bone resorption by affecting osteoclasts and developing to secondary osteoporosis. Therefore, PJI-induced inflammation may contribute to the development of osteoporosis by altering osteoclast function.

Echinacoside (ECH), a natural phenylethanoid glycoside, is the main active ingredient presenting in Cistanche salsa, which has been reported to be extensively studied in neuroprotection (Lu et al., 2016), antitumor (Ye et al., 2019), anti-aging (Wu et al., 2019), and myocardial remodeling (Ni et al., 2021), antioxidant (Chuang et al., 2022), anti-apoptotic (Zhang et al., 2017), and anti-inflammatory (Zhou et al., 2020). Studies have demonstrated that ECH alleviates hypoxia-induced memory impairment (Zheng et al., 2019), inhibits the development of breast cancer (Bian et al., 2021), alleviates inflammatory bowel disease (Li et al., 2018), and improves heart failure by reversing myocardial remodeling (Ni et al., 2021). ECH has also been found to promote bone formation by enhancing osteoblast proliferation and differentiation as well as alleviating the reduction in bone mass caused by diabetes (Gong et al., 2019) or ovariectomy (Liu et al., 2019). To our knowledge, the effects of ECH on osteoclast differentiation and bone resorption of infected osteolysis remain unknown.

Herein, this study aimed to explore the effects of ECH on osteoclasts differentiation and bone resorption function *in vitro* and *in vivo*, and investigate the regulatory mechanisms of ECH treatment by using an established PJI rat model (Wei et al., 2021a; Wei et al., 2021b), to provide an important experimental basis for elucidating the role and mechanism of ECH in infected-induced osteolysis and important practical implications for guiding the treatment of clinical PJI patients.

## 2 Material and Methods

### 2.1 Osteoclast Culture

Fresh primary bone marrow cells were extracted from lower limb bone of 4 weeks old rat. Briefly, under aseptic conditions, long bone tissue from the lower extremities of rats was cut and the bone marrow cavity was flushed with PBS (Servicebio, China) until it turned from red to white. The fluid was collected and centrifuged, and then the supernatant was discarded. The cells were lysed with erythrocyte lysate (Servicebio, China) for 10 min and centrifuged again, then the supernatant was discarded. After washing the cells twice with PBS, the cells were resuspended with a complete medium supplemented and then inoculated in culture flasks. 24 h later, the flask liquid was collected, centrifuged and the supernatant discarded to obtain cells, which were cultured for 3 d in a complete medium supplemented with 100 ng/ml M-CSF (MedCemExpress, United States) to obtain bone marrow-derived macrophages (BMMs). Then, we induced BMMs differentiation with 100 ng/mL M-CSF and 50 ng/ml RANKL (R&D systems, United States) for 4 d.

### 2.2 Cytotoxicity Assay

**FIGURE 1:**
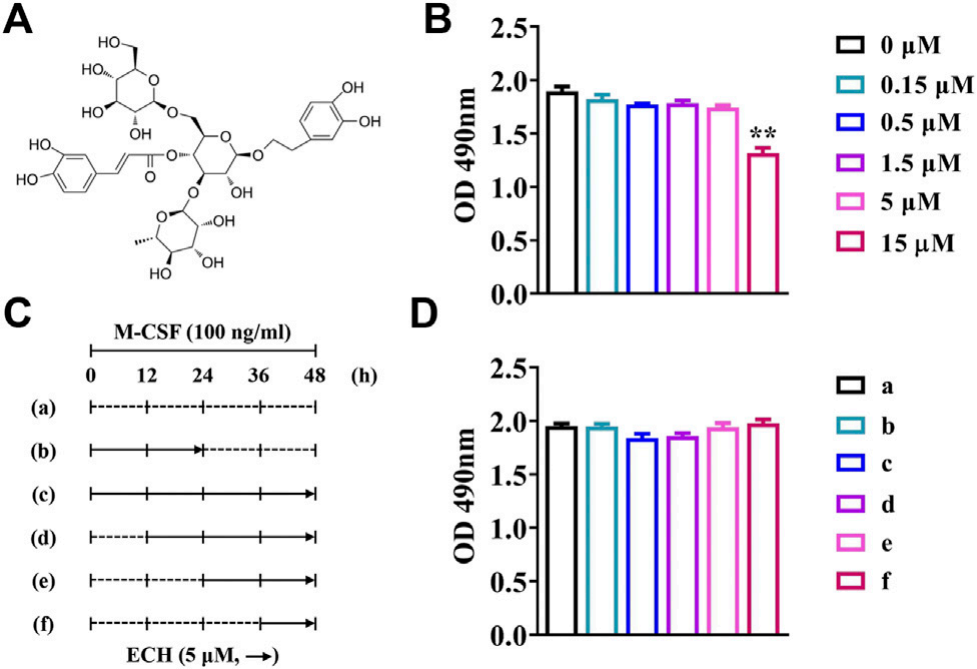
The cytotoxicity of ECH at different concentrations and different times **(A)** Chemical structure of ECH **(B)** The cytotoxic effect of ECH on BMMs at different concentrations was measured by the MTS assay **(C)** Schematic diagram of ECH treatment at different periods and times **(D)** The cytotoxic effect of ECH (5 μM) on BMMs at different times was measured by the MTS assay. Mean ± S.E.M., the experiment was performed six times independently, the quantitative data shown were pooled from multiple experiments. ^*^ *p* < 0.05, ^**^ *p* < 0.01 (Compared with 0 μM of ECH). One-way ANOVA followed by Tukey’s post hoc test was performed for multiple group comparisons. OD: Optical density; M-CSF, Macrophage colony-stimulating factor; ECH, Echinacoside; BMMs, Bone marrow-derived macrophages; MTS, Multiple tumor suppressor.

The 3-(4,5-dimethyltiazol-2-yl)-5-(3-carboxymethoxyphenyl)-2-(4-sulfophenyl)-2H-tetrazolium (MTS) assay kit (Promega, United States) was used to assess cell proliferation. BMMs (3 × 10^4^ cells/well) were inoculated in 96-well plates and incubated with culture medium (including M-CSF) overnight. Echinacoside (ECH) was commercially purchased from Merck (Germany) and added into the wells as shown in Figure 1. MTS solution (20 μl/well) was added into each well 48 h later and incubated for 2 h at 37°C. The effects of compounds on cells were measured by absorbance at 490 nm using a spectrophotometer (BioTek, United States).

### 2.3 Tartrate-Resistant Acid Phosphatase (TRAP) Staining

In brief, after removing the medium, the cells were fixed by adding 4% paraformaldehyde and the cells membrane was broken by adding 0.1% Triton. After cleaning with PBS, tartrate-containing buffer and chromogenic substrate were added. After incubation at 37°C for 1 h, washing with PBS, redyeing with hematoxylin for 2 min, and washing with ddH2O, cells were stained for TRAP enzymatic activity according to the TRAP staining kit scheme (Sigma, United States). NIS Elements BR light microscope (Nikon, Japan) was used for photography, and ImageJ software was used for quantification analyses.

### 2.4 F-Actin Staining

After removing the medium, the cells (6 × 10^4^ cells/well) were fixed by adding 4% paraformaldehyde for 30 min and the cells membrane was broken by adding 0.1% Triton for 10 min. The cells were added F-actin fluorescent dye (Beyotime, China) followed by cleaning with PBS. After incubation at room temperature without light for 30 min and then washing with PBS, the cells were redyed with DAPI for 10 min without light. The cells were imaged using an inverted fluorescence microscope (Nikon, Japan), and ImageJ software was used for quantification analyses.

### 2.5 Bone Resorption Assay *in Vitro*

As for bone resorption, BMMs (3 × 10^3^ cells/well) were cultured on sterile bovine bone plates, using the same culture methods and reagents as before. After 6 days of induction, the cells were rinsed three times with 10% sodium hypochlorite and water. Finally, the scanning electron microscope using Gatan digital camera system (Zeiss, Germany) was used to take pictures, and ImageJ software was used for quantification analyses.

### 2.6 RNA Extraction and Quantitative Real-Time Polymerase Chain Reaction (qRT-PCR)

BMMs were pretreated with gradient concentrations of ECH for 5 days followed by 50 ng/ml RANKL with or without ECH for 12 h. Total RNA from BMMs was extracted utilizing TRIzol reagent (Invitrogen, Carlsbad CA, United States) in accordance with the manufacturer’s protocol. The isolated RNA was collected and quantify by Nanodrop 3000 (United States). cDNA was generated from RNA samples using M-MLV reverse transcriptase and oligo dT primers (Promega). RT-qPCR was performed using diluted cDNA. Total RNA from periprosthetic bone tissues was extracted using Trizol reagent. In brief, 20 mg of bone tissues was placed into a 1.5 ml EP tube, with 3 zirconia beads and 1 ml of Trizol. The EP tubes were placed in a homogenizer (China; 60HZ, 4 min, 4°C). After sufficient homogenization, the EP tube was added with 200 µl of chloroform and shaken vigorously for 30 s, and then placed on ice for about 10–15 min. After centrifugation (12000 rpm, 4°C, 15 min), the supernatants were carefully aspirated and transferred to a new EP tube, with an equal volume of isopropanol, mixing the mixture upside down and then leave at room temperature for 10 min after centrifugation (12000 rpm × 10 min, 4°C), the supernatant was carefully discarded. After centrifugation (12000 rpm × 10 min, 4°C), the supernatant was carefully discarded and the RNA precipitate was retained. The RNA was then washed twice with pre-cooled 75% ethanol and finally mixed with appropriate amount of ultrapure water. The isolated RNA, generated cDNA and RT-qPCR procedure was performed following the above protocol. The PCR primers for amplification of rat, including Acid phosphatase 5 (Acp5), Cathepsin K (CtsK), Protooncogene c-Fos (c-Fos), Nuclear factor of active T cells 1 (NFATc1), and glyceraldehyde phosphate dehydrogenase (GAPDH) are presented in Table 1. All primers were synthesized by Sangon Biotech (China). The relative amounts of the mRNA levels of the target genes were normalized to GAPDH and calculated by using the 2^−ΔΔCT^ method.

**TABLE 1:** Rat oligonucleotide primers and reaction conditions used in qRT-PCR.

Genes	Forward primers (5′-3′)	Reverse primers (5′-3′)	Annealing (°C)
Acp5	CAA​AGA​GAT​CGC​CAG​AAC​CG	GAG​ACG​TTG​CCA​AGG​TGA​TC	60
CtsK	CAG​AGG​CCA​CAA​CTC​TCA​GAA	GTG​TCC​ATC​GAT​GCA​AGC​TT	60
c-Fos	AGC​TCC​CAC​CAG​TGT​CTA​CC	TCA​CCG​TGG​GGA​TAA​AGT​TGG	60
NfATc1	CCG​TTG​CTT​CCA​GAA​AAT​AAC​A	TGT​GGG​ATG​TGA​ACT​CGG​AA	60
Oscar	CGATTGGCACAGCAGGCG	AAG​ACA​CAT​GAA​GGA​AAT​AGA​G	60
Mmp9	TCG​AAG​GCG​ACC​TCA​AGT​G	GCG​GCA​AGT​CTT​CGG​TGT​AG	60
GAPDH	GCC​TCC​AAG​GAG​TAA​GAA​AC	GTC​TGG​GAT​GGA​ATT​GTG​AG	60

qRT-PCR, quantitative real-time polymerase chain reaction; Acp5, Acid phosphatase 5; CtsK, Cathepsin K; c-Fos, Protooncogene c-Fos; NFATc1, Nuclear factor of active T cells 1; GAPDH, glyceraldehyde phosphate dehydrogenase.

### 2.7 Western Blotting Analysis

Osteoclasts were induced to differentiate 2 d after addition of RANKL. Then cells were changed with fresh medium containing serum and cytokine, and treated with ECH in the meantime. After holding for 2 d, cells were collected with RIPA lysate (Servicebio, China) containing 1% PMSF (Servicebio, China), and then proteins were extracted. The corresponding protein expression in osteoclasts was detected by the western blotting technique. Briefly, after protein quantification, proteins were denatured, separated on SDS-PAGE gels, and transferred onto PVDF membranes (EMD Millipore, Burlington, MA, United States). The membranes were immunoblotted with primary rabbit antibody for PI3K, p-Akt, Akt, c-Fos, NFATc1, and CtsK (diluted 1:1000, respectively; Abclonal, China), primary mouse antibody for GAPDH (diluted 1:5,000; Abclonal, China) overnight at 4°C. The next day, membranes were washed and then incubated with horseradish peroxidase-conjugated secondary antibodies (goat anti-mouse IgG and goat anti-rabbit IgG; diluted 1:5,000, respectively; Abclonal, China) at room temperature for 1 h. Antibodies were detected with enhanced chemiluminescence substrate (PerkinElmer, United States), and ImageJ software was used for quantification analyses.

### 2.8 Immunofluorescence (IF) Analysis

BMMs were fixed with 4% paraformaldehyde under 4°C for 15 min and then added with a blocking solution (10% goat serum-PBS) for 2 h at room temperature. Rabbit anti-c-Fos antibody (diluted 1:100; Abclonal, China) was added and incubated overnight at 4°C in a humid chamber. After washing with deionized water 5 times, FITC goat anti-rabbit IgG (H + L) (diluted 1:5,000; Abclonal, China) secondary antibody was incubated in the dark at room temperature for 60 min. The cells were added DAPI, and incubated in the dark for 10 min at room temperature following washing 3 times with PBS, then rinsing with deionized water for 15 min. Images were collected using an inverted fluorescence microscope (Nikon, Japan). The Image-Pro Plus 6.0 (Media Cybernetics, Silver Spring, MD, United States) was used for quantification.

### 2.9 Periprosthetic Joint Infection-Induced Bone Loss Rat Model

The protocol of all animal experiments was approved by the Committee on the Ethics of Animal Experiments of the School of Medicine, Wuhan University. All animal experimental procedures were performed following the Guidelines for the Care and Use of Laboratory Animals of the Chinese Animal Welfare Committee. Forty Wistar rats (male, 255 ± 6 g, 10 weeks old) were supplied by the Animal Experiment Center of Zhongnan Hospital (Wuhan, China). All rats were randomly divided into four groups: Control group (femur prosthesis implantation (FPI) surgery without infection, *n* = 10), PJI group (FPI surgery with infection, *n* = 10), PJI + debridement (DEB) group (*n* = 10), and PJI + DEB + ECH (20 mg/kg) group (*n* = 10). The surgical procedure was performed based on previously described rat PJI models (Wei et al., 2021a; Wei et al., 2021b). Briefly, after general anesthetization by inhalation delivered via nose cone with 2.5% isoflurane, the right legs of all rats were shaved and disinfected. The knee was surgically exposed, and a 1.4-mm hole was drilled into the femoral canal just anterior to the Blumensaat line. The prosthesis (diameter 1.5 mm, length 8 mm) was manually placed through retrograde insertion with a screwdriver, with 1 mm screw cap protruding into the joint. After closing the capsule, 40 µl of 1.5 × 10^7^ CFU/ml *S. aureus* (ATCC 25923) was injected into the articular cavity of knee assigned to the PJI rat model. On days 7 after surgery and bacterial inoculation, debridement and retention of the prosthesis procedures were carried out for those rats of PJI + DEB and PJI + DEB + ECH groups. The rats in the PJI + DEB + ECH group were intraperitoneally injected of ECH at 20 mg/kg every day for 4 weeks after debridement. The other groups of rats were intraperitoneally injected with PBS as vehicle control. In addition, we have previously performed comparative studies with the PJI rat model and the ovariectomy (OVX) + FPI surgery rat model. Briefly, the animals were divided into three groups: 1) Control group (FPI surgery, *n* = 8), 2) PJI group (FPI surgery with infection, *n* = 8), 3) OVX + FPI group, *n* = 8. Micro-CT was carried out at post-surgical 6 weeks to analyze changes in distal femur bone mass around the prosthesis. The results are shown in Supplementary Figure S1. Both the PJI group and OVX + FPI group showed a significant reduction in bone mass compared with the control group (*p* < 0.01). However, no statistical differences were observed between the PJI group and OVX + FPI group (*p* > 0.05, Supplementary Figure S1). Thus, our previous studies suggested that the PJI-induced osteolysis rat model established in this study was reliable. Table 2 reports the allocation of animals per group and the relative analysis.

**TABLE 2:** Allocation of animals per group and investigations are as follows: the surgery, X-ray, and Micro-CT detection are performed in all animals per group; the assigned animals per group of numbers 1 to 5 are used for femur H&E, TRAP, and immunofluorescence staining, while numbers 6 to 10 are used for qRT-PCR.

Analyses	Number of animals
Total animals (*n* = 10 per group)	1 2 3 4 5 6 7 8 9 10
Arthroplasty surgery	x x x x x x x x x x
X-ray	x x x x x x x x x x
Micro-CT	x x x x x x x x x x
Femur H&E staining	x x x x x
Femur TRAP staining	x x x x x
Femur Immunofluorescence staining	x x x x x
qRT-PCR (periprosthetic bone tissues)	x x x x x

Micro-CT: Micro-Computed Tomography; TRAP: Tartrate-Resistant Acid Phosphatase; H&E: hematoxylin and eosin; qRT-PCR: quantitative real-time polymerase chain reaction.

### 2.10 X-Ray and Micro-Computed Tomography (Micro-CT)

X-ray images were taken using the Bruker Xtreme BI (Germany; filter: 0.4 mm, 45 kvp, exposure time: 1.2 s, bin: 1 × 1 pixels, F Stop: 2) to determine the position of the prosthesis and osteolysis around the prosthesis. Femur bone was scanned and analyzed by Skyscan1276 micro-CT system (Bruker, Germany) using the following settings: voltage 85 kV; filter 1 mm; current 200 μA; exposure time 384 ms; image pixel size: 17.420 μm, including bone volume per trabecular volume (BV/TV), trabecular number (Tb. N), trabecular thickness (Tb. Th) and trabecular separation (Tb. Sp). All the above parameters refer to the guidelines of micro-CT for evaluating bone microstructure (Bouxsein et al., 2010).

### 2.11 Bone Histomorphometry Analysis

All samples were fixed in 4% paraformaldehyde for 48 h, then decalcified with EDTA for 28 d, dehydrated, embedded with paraffin, and finally sectioned into 4 µm slices. After the slices were dewaxed and washed in PBS, hematoxylin and eosin (H&E), TRAP and IF staining were performed. H&E staining method for tissue was as follows. Paraffin sections were first dewaxed with xylene and then dehydrated through graded ethanol of decreasing concentration (100%, 90%, 80%, 70%; 5 min/concentration, respectively), hematoxylin staining was performed for 5min after washing with PBS for 5 min (triplicate), then washing with PBS for 5 min (twice). Eosin staining was performed for 3 min and then washed with PBS for 30 s (twice), and then dehydrated through graded ethanol of increasing concentration (70%, 80%, 90%,100%; 30 s, respectively). Finally, the slices were rehydrated and sealed with a neutral resin. TRAP staining method for tissue was as follows. Paraffin sections were first dewaxed with xylene and then dehydrated through graded ethanol of decreasing concentration (100%, 90%, 80%, 70%; 5 min/concentration, respectively), washing with PBS for 5 min (triplicate), then soaked in preheated PBS at 37°C for 10 min, incubated the staining with the prepared TRAP working solution (Sigma, United States) for 2 h at 37°C, followed by washing with PBS 3 times. Hematoxylin staining was performed for 3 min and then washed with PBS. Differentiation and blue return with differentiation and blue return solution was carried out. Finally, the slices were rehydrated and sealed with a neutral resin. For bone tissue IF staining, paraffin sections were first dewaxed to water, then antigen repair was performed in a microwave oven with EDTA antigen repair buffer (medium heating for 3 min, low heating for 10 min). After drawing circles around the tissue with a histochemical pen, 3% BSA (Roche, United States) was added dropwise to the section and blocked for 30 min. The subsequent treatment was referred to the protocols used for cells in section 2.8.

### 2.12 ELISA Assay

Serum samples were obtained by centrifugation (3000 rpm, 4°C,15 min) at 4 weeks after debridement and ECH treatment. Serum creatinine (Cr), urea nitrogen (UN), alanine aminotransferase (ALT) and aspartate aminotransferase (AST) of rats in each group were measured by ELISA kit (Huamei Biotech Co., Ltd., China).

### 2.13 Statistical Analysis

Data were analyzed using GraphPad Prism software (version 8.0, La Jolla, CA, United States). All data were expressed as mean ± standard error of the mean (S.E.M). One-way ANOVA test was used for multi-group comparison, followed by Dunnett *t*-test to determine whether the difference between the two groups was significant. A value of *p* < 0.05 was considered statistically significant.

## 3 Results

### 3.1 Echinacoside Inhibited RANKL-Induced Osteoclast Formation Without Cytotoxicity

The results of the MTS assay showed that no significant inhibition of the proliferation of BMMs was observed when the ECH concentration was no more than 5 μM (*p* < 0.01, Figure 1B). No significant effects of inhibiting the proliferation of BMMs was observed while ECH was given at a concentration of 5 μM both the administration period (early/late) and the duration of administration (12/24/36/48 h) (Figure 1C,D).

**FIGURE 2:**
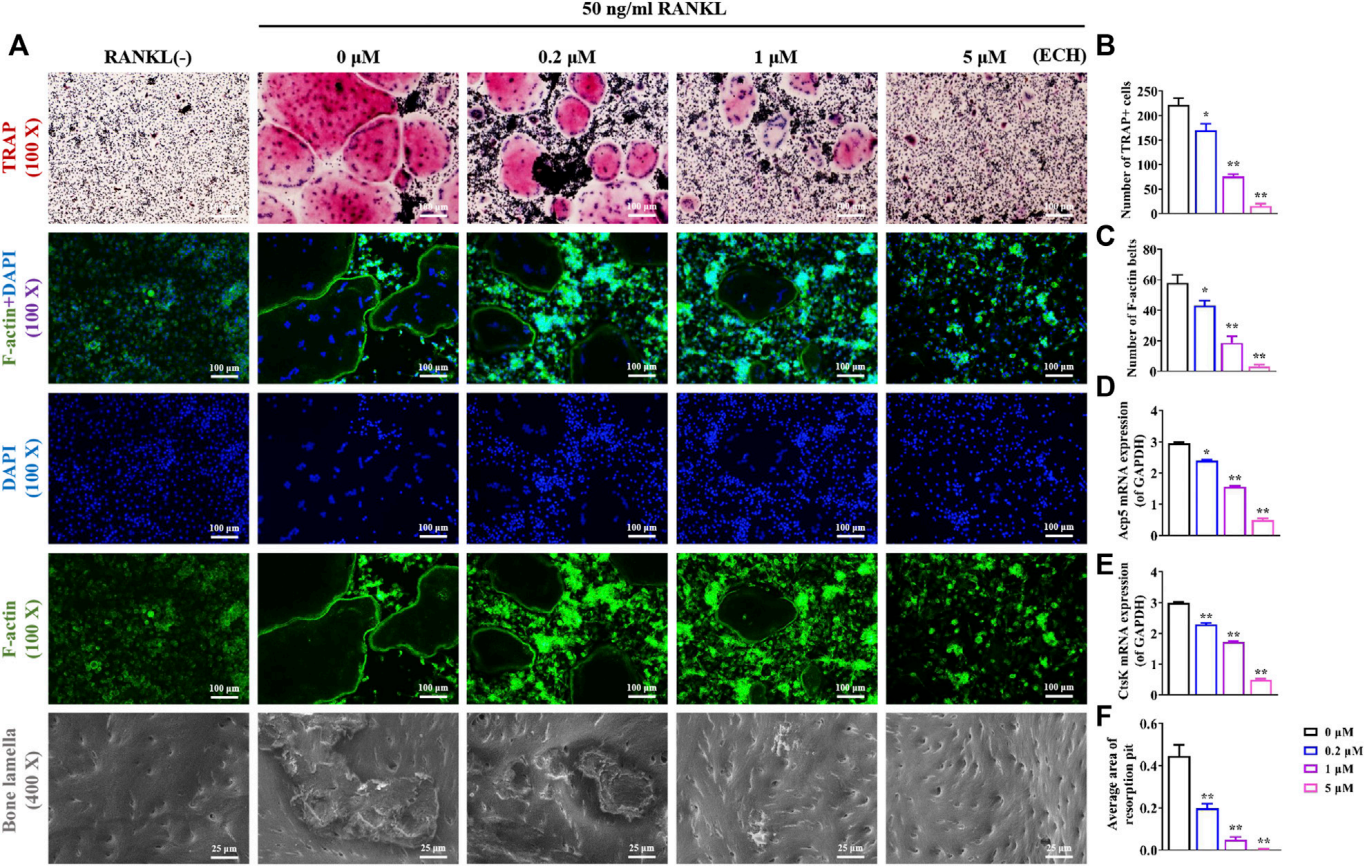
ECH inhibited osteoclasts differentiation, F-actin formation and bone resorption function **(A)** Representative images of TRAP staining, F-actin staining and bone lamella resorption. TRAP^+^ osteoclasts were stained as Red, F-actin belts were stained as Green and nuclei were stained as Blue **(B,C)** Quantification analyses of the number of TRAP^+^ osteoclasts and F-actin belts **(D,E)** Acp5 and Ctsk mRNA expression in osteoclasts treated with ECH at different concentrations **(F)** Quantification analyses of the average area of resorption pit. Mean ± S.E.M., the experiments of TRAP, F-actin staining and bone lamella resorption were performed three times independently, the experiment of mRNA expression was performed six times independently, the quantitative data shown were pooled from multiple experiments. ^*^ *p* < 0.05, ^**^ *p* < 0.01 (Compared with 0 μM of ECH). One-way ANOVA followed by Tukey’s post hoc test was performed for multiple group comparisons. TRAP, Tartrate resistant acid phosphatase; Acp5: Acid phosphatase 5; CtsK, Cathepsin K, ECH, Echinacoside.

As shown in Figure 2A, the addition of RANKL significantly promoted osteoclast differentiation and formation. Besides, an inhibitory effect of different concentrations of ECH on osteoclast differentiation and bone resorption function was detected. TRAP staining results showed that the control group presented mature TRAP^+^ osteoclasts. In contrast, osteoclast development was significantly inhibited in the ECH group, along with a concentration-dependent phenomenon (Figure 2A). The visualization indicated that ECH significantly reduced the number of TRAP^+^ osteoclasts (*p* < 0.05, *p* < 0.01, Figure 2B) and inhibited the expression of the osteoclast marker genes Acp5 and CtsK (*p* < 0.05, *p* < 0.01, Figures 2D,E). The F-actin belt is an important symbol of mature osteoclasts, and an intact F-actin belt is necessary for bone resorption function (Sun et al., 2019). To further verify the inhibiting osteoclastogenesis of ECH, we performed the F-actin belt staining assay. The number and mean size of F-actin belts after ECH treatment were significantly lower than those of the control group (*p* < 0.05, *p* < 0.01, Figure 2C). The bone plate resorption images were shown in Figure 2A, the control group presented large resorption pits, while the resorption pits reduced in varying degrees after ECH treatment and showed a concentration-dependent reduction (*p* < 0.01, Figure 2F). These data suggested that ECH significantly inhibited RANKL-induced osteoclastogenesis and F-actin belt formation as well as bone resorption function of osteoclasts in the absence of cytotoxicity.

### 3.2 Echinacoside Inhibited Osteoclasts Differentiation and Function by Preventing PI3K/Akt/C-Fos Pathway *in vitro*

**FIGURE 3:**
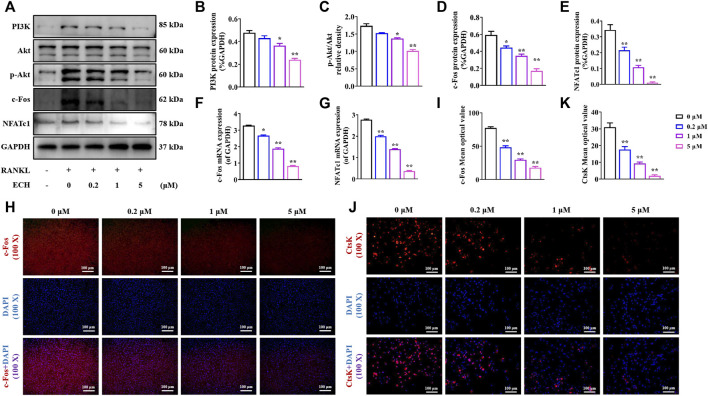
ECH reduced PI3K/Akt/c-Fos pathway in osteoclasts **(A)** PI3K, P-Akt, Akt, c-Fos, NFATc1 and CtsK protein expression in osteoclasts treated with ECH at different concentrations **(B)** Immunofluorescence staining for c-Fos with DAPI in osteoclasts treated with ECH at different concentrations. C-Fos protein was stained as Red and nuclei were stained as Blue **(C)** Quantification analyses of PI3K protein expression **(D)** Quantification analyses of p-Akt/Akt relative density **(E–G)** Quantification analyses of c-Fos, NFATc1 and CtsK protein expression **(H,I)** C-Fos and NFATc1 mRNA expression in osteoclasts treated with ECH at different concentrations **(J)** Quantification analyses of c-Fos mean optical density. Mean ± S.E.M., the experiments of protein expression and immunofluorescence staining were performed three times independently, the experiment of mRNA expression was performed six times independently, the quantitative data shown were pooled from multiple experiments. ^*^ *p* < 0.05, ^**^ *p* < 0.01 (Compared with 0 μM of ECH). One-way ANOVA followed by Tukey’s post hoc test was performed for multiple group comparisons. PI3K: Phosphatidylinositol 3 kinase; P-Akt, P-protein kinase B, c-Fos: Protooncogene c-Fos, NFATc1, Nuclear factor of active T cells 1; CtsK, Cathepsin **(K)**; ECH, Echinacoside.

PI3K/Akt signaling is an important pathway for RANKL-induced osteoclastogenesis. The RANKL/RANK complex leads to osteoclast PI3K activation and Akt phosphorylation, further upregulating osteoclast-associated important transcription factor protooncogene c-Fos, which mediates osteoclast differentiation and maturation (Baek et al., 2016). It has been found that Rehmanniae Radix Praeparata which contained ECH could enhance osteoblastic bone formation via PI3K/Akt pathway in diabetic rats (Gong et al., 2019). It has also been confirmed that ECH can induce nitric oxide production via PI3K/Akt pathway in endothelial cells (Gu et al., 2020). ECH can also inhibit the proliferation and migration of ovarian cancer cells via PI3K/Akt/mTOR pathway (Liu et al., 2022). These above studies suggested that PI3K/Akt/c-Fos pathway may be an important downstream pathway of ECH. To further investigate the specific mechanism of inhibiting osteoclast differentiation and function by ECH, we examined the effect of ECH on the PI3K/Akt/c-Fos pathway. As shown in Figure 3A, RANKL significantly activated the PI3K/Akt/c-Fos/NFATc1 signaling pathway in osteoblasts. Quantitative results showed that ECH concentration-dependently inhibited PI3K expression and Akt phosphorylation (*p* < 0.05, *p* < 0.01, Figures 3A–C) and the expression of the c-Fos gene (*p* < 0.05, *p* < 0.01, Figure 3F) and protein (*p* < 0.05, *p* < 0.01, Figures 3A,D, 3H,I), further down-regulated the downstream NFATc1 (*p* < 0.01, Figures 3A,E,G) as well as CtsK expression (*p* < 0.01, Figures 3J,K). These data suggested that ECH exerted an inhibitory effect on osteoclasts through the PI3K/Akt/c-Fos signaling pathway.

### 3.3 Echinacoside Alleviated Periprosthetic Joint Infection-Induced Bone Loss

**FIGURE 4:**
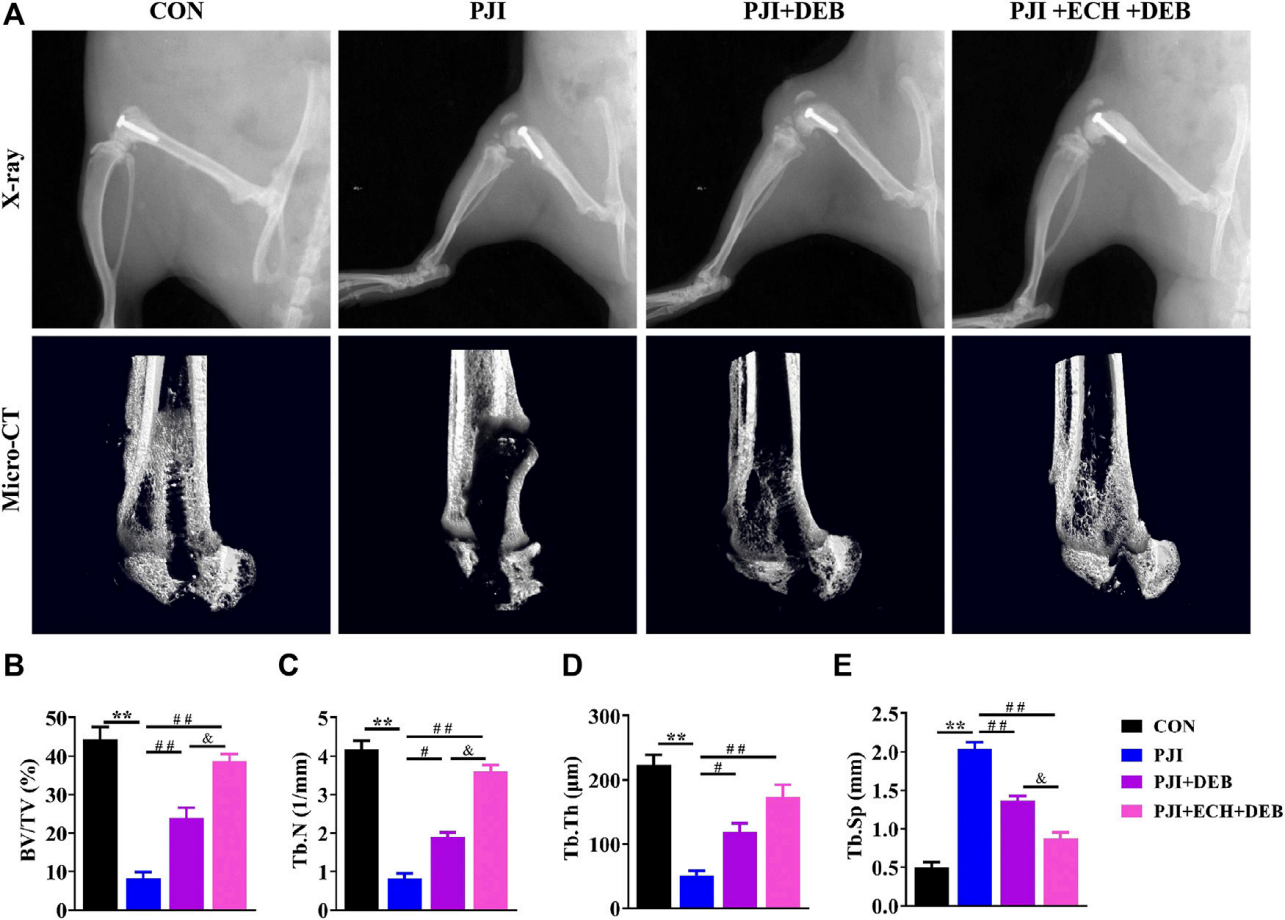
ECH alleviated bone loss induced by PJI **(A)** Representative X-ray and micro-CT images in each treatment group of rats **(B–E)** Quantification analyses of all bone sections, including BV/TV, Tb.N, Tb.Th, Tb. Sp. Mean ± S.E.M., *n* = 10 per group for X-ray and micro-CT analysis. ^**^ *p* < 0.01 (Compared with the CON group), ^#^ *p* < 0.01, ^##^ *p* < 0.01(Compared with the PJI group), ^&^ *p* < 0.05 (Compared with the PJI + DEB group). One-way ANOVA followed by Tukey’s post hoc test was performed for multiple group comparisons. CON, Control; PJI, Periprosthetic joint infection; ECH, Echinacoside; DEB, Debridement; BV/TV, Bone volume per tissue volume; Tb.N, Trabecula number; Tb.Th, Trabecular thickness; Tb. Sp, Trabecula separation.

After determining the inhibition of osteoclast production and function by ECH, we investigated the inhibitory effect of ECH on bone erosion of PJI rats and evaluated the potential of ECH as a therapeutic agent for inflammatory osteolysis. We administrated ECH (20 mg/kg) intraperitoneally once daily for 4 weeks after debridement in PJI rats. No adverse effects or deaths were recorded after both PJI modeling and ECH treatment. No obvious structural changes were observed in the kidney and liver of each treatment group (Supplementary Figure S2A,B). No significant differences were detected in the serum Cr, UN, ALT and AST among each treatment group (*p* > 0.05, Supplementary Figure S2C–F). X-rays showed that the PJI group exhibited significant periprosthetic osteolysis compared to the control group (without infection). Osteolysis was significantly alleviated in the DEB combined with ECH treatment group, which was superior to that of the DEB group (Figure 4A). Micro-CT images and analysis revealed that a significant increase of bone volume (BV/TV, Tb. N, Tb. Th, and Tb. Sp) were detected in the ECH treatment group compared with the PJI and DEB group, but lower than the control group (*p* < 0.05, *p* < 0.01, Figures 4A–E). This suggested that ECH treatment significantly improved bone mass of PJI-induced rat model.

### 3.4 Echinacoside Attenuated Osteoclasts Function by Down-Regulating c-Fos/NFATc1 in Periprosthetic Joint Infection Rats

**FIGURE 5:**
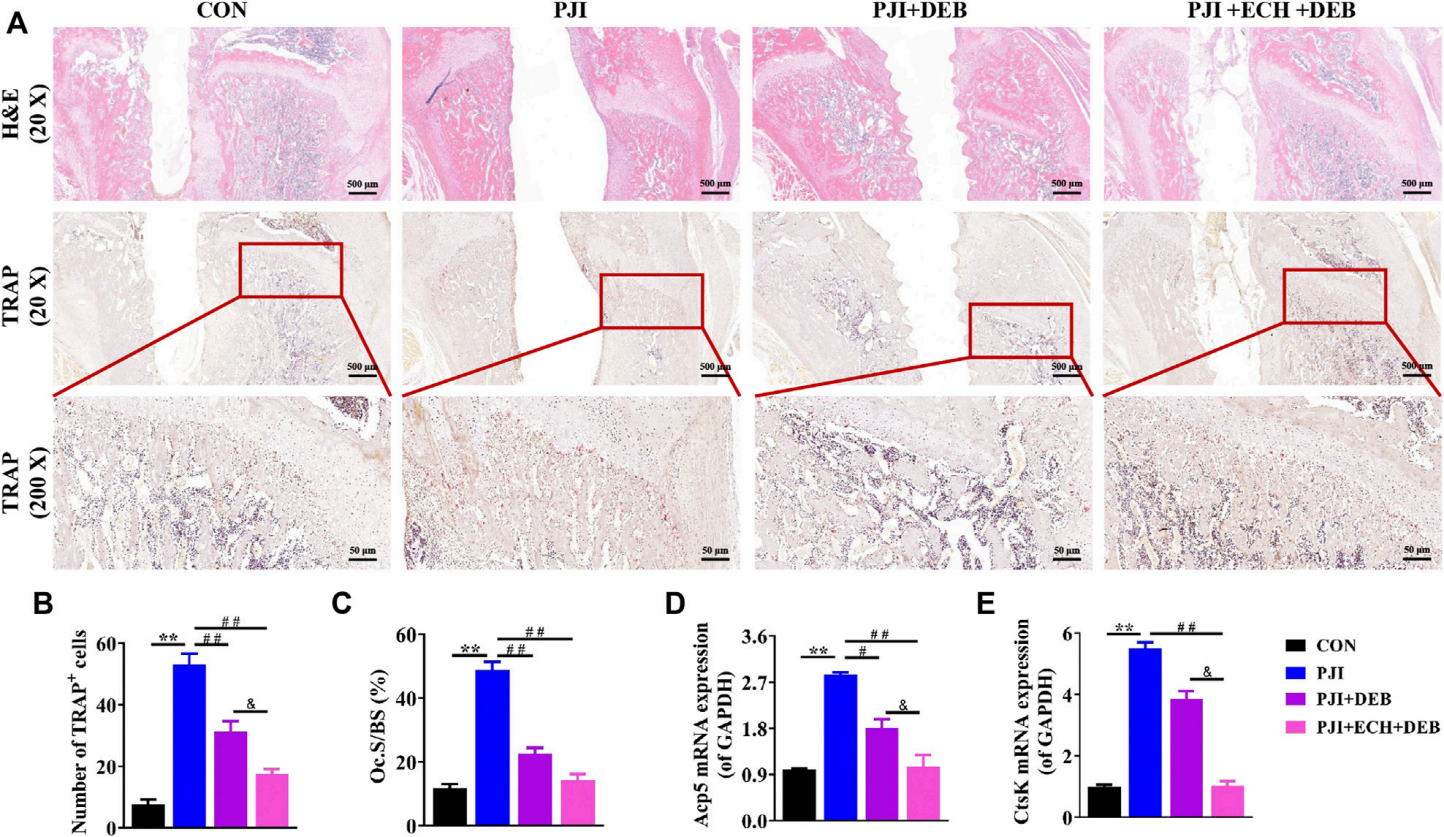
ECH inhibited osteoclast function and improved bone mass in PJI rats **(A)** Representative images of H&E and TRAP staining of decalcified bone sections **(B)** Quantification analyses of the number of TRAP^+^ osteoclasts per high magnification field of view **(C)** Quantification analyses of osteoblast surface per bone surface **(D,E)** Acp5 and CtsK mRNA expression in rat bone tissue. Mean ± S.E.M., n = 5 per group for H&E staining, TRAP staining and mRNA expression. ^**^ *p* < 0.01 (Compared with the CON group), ^#^ *p* < 0.01, ^##^ *p* < 0.01(Compared with the PJI group), ^&^ *p* < 0.05 (Compared with the PJI + DEB group). One-way ANOVA followed by Tukey’s post hoc test was performed for multiple group comparisons. CON: Control; PJI: Periprosthetic joint infection; ECH: Echinacoside; DEB: Debridement; H&E: Hematoxylin and eosin; TRAP: Tartrate resistant acid phosphatase; Oc. S/BS: Osteoblast surface per bone surface; Acp5: Acid phosphatase 5; CtsK: Cathepsin K.

Based on the X-ray and Micro-CT results, we performed a histological study. H&E staining suggested that a significant decrease in bone destruction around the prosthesis were observed in the ECH and DEB treatment group compared with the PJI and DEB group (Figure 5A). TRAP staining and quantitative analysis showed that larger number of TRAP^+^ osteoclasts were detected in the PJI group than the control group, while these changes were significantly improved by ECH and DEB treatment (*p* < 0.01, Figures 5A,B). The osteoclast surface/bone surface (Oc.S/BS) was significantly reduced (*p* < 0.01, Figure 5C), and Acp5 and CtsK gene expression was significantly downregulated (*p* < 0.01, Figures 5D,E). These anti-osteoclast effects in ECH and DEB group were better than those of DEB group, but lower than the control group.

**FIGURE 6:**
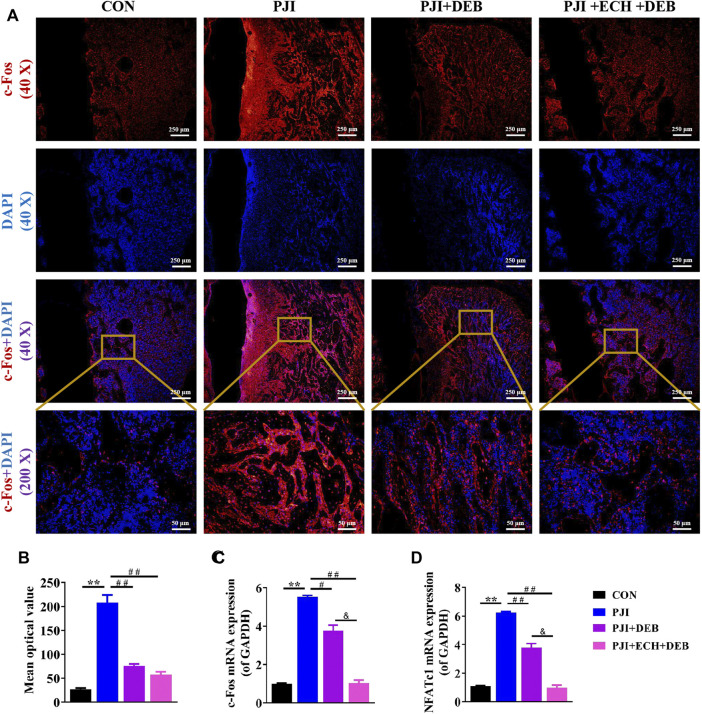
ECH inhibited osteoclast function by down-regulating c-Fos/NFATc1 in PJI rats **(A)** Immunofluorescence staining for c-Fos with DAPI in decalcified bone sections **(B)** Quantification analyses of c-Fos mean optical density **(C,D)** C-Fos and NFATc1 mRNA expression in rat bone tissue. Mean ± S.E.M., *n* = 5 per group for immunofluorescence staining and mRNA expression. ^**^ *p* < 0.01 (Compared with the CON group), ^#^ *p* < 0.01, ^##^ *p* < 0.01(Compared with the PJI group), ^&^ *p* < 0.05 (Compared with the PJI + DEB group). One-way ANOVA followed by Tukey’s post hoc test was performed for multiple group comparisons. CON, Control; PJI, Periprosthetic joint infection; ECH, Echinacoside; DEB, Debridement; c-Fos, Protooncogene c-Fos; NFATc1, Nuclear factor of active T cells 1.

Moreover, we examined the effects of ECH on c-Fos/NFATc1 expression *in vivo*. The results showed that both the gene and protein expression of c-Fos and NFATc1 were significantly upregulated in the PJI group compared with the control group, while these changes were significantly inhibited after DEB and ECH and DEB treatment. The inhibitory effects of ECH and DEB group were stronger than that in the DEB group alone (*p* < 0.01, Figures 6A–D). The above data indicated that ECH significantly reduced osteoclast function and alleviated the PJI-induced bone loss by downregulating c-Fos/NFATc1.

## 4 Discussion

In recent years, an increasing number of studies have found that infections or aseptic inflammatory conditions (e.g., infected osteomyelitis, septic arthritis, AIDS, rheumatoid arthritis, and inflammatory bowel disease) can contribute to the development of secondary osteoporosis (Sakurai et al., 2003; Mundy, 2007; Dapunt et al., 2015; Gallego-Escuredo et al., 2017; Rychter et al., 2021). Redlich introduced the concept of inflammatory bone loss, namely inflammation causes bone loss by disrupting bone metabolism, activating bone degradation, and inhibiting bone remodeling (Redlich and Smolen, 2012). The occurring of inflammatory diseases might bring systemic effects on the bone mass as well as increase the risk of osteoporosis and fracture. Thus, the potential pathogenesis and signal pathways of inflammatory bone loss, as well as exploring effective therapeutic agents attract the attention of the scholars increasingly. In this study, we investigated the pathogenesis and potential therapeutic agents of inflammatory bone loss caused by infection using the PJI rat model as an entry point. We observed severe osteolysis with abnormal hyperfunction of osteoclasts in the PJI rat model. Administration of ECH to PJI rats resulted in a significant reduction in bone loss and significant inhibition of osteoclast function. This study expanded the concept of inflammatory bone loss and provided a theoretical and experimental basis for strategies to prevent and treat inflammatory osteolysis.

Most current studies point to the emerging anti-inflammatory and neuroprotective effects of ECH(Zhou et al., 2020; Zeng et al., 2021). It had been reported that ECH could promote osteoblast mineralization through upregulation of OPG/RANKL (Li et al., 2012) and osteoblast proliferation and differentiation through the Wnt/β-catenin signaling pathway (Tang et al., 2020). Therefore, based on the above studies, we speculated and confirmed that ECH have a potential anti-osteoclast function in this study. TRAP staining is a standard method used to detect osteoclast differentiation (Hayman, 2008), and the F-actin belt is the most distinctive feature of mature osteoclasts (Marchisio et al., 1984), as well as the bone plate resorption assay is used to measure the bone resorption capacity of osteoclasts. In the present experiment, the concentration-dependently inhibition of ECH in the RANKL-induced osteoclast differentiation and activity was observed. F-actin staining showed that ECH exhibited concentration-dependent inhibition of osteoclast F-actin belts formation. The *in vitro* bone resorption results indicated that ECH significantly inhibited the formation of pits in the bone plate, with a concentration-dependent attenuation effect. According to the above data of anti-osteoclast function by ECH, we further performed ECH intervention treatment on the PJI rat model. After debridement and 4-week intraperitoneal injection of ECH, the bone mass of PJI rats was significantly improved, accompanied by down-regulation of c-Fos expression and significant inhibition of osteoclast function. Thus, these results confirmed the emerging role of ECH in inhibiting osteoclast function and alleviating infected osteolysis.

Several studies have identified an important role for the PI3K/Akt/c-Fos pathway in the regulation of osteoclast function (Suh et al., 2013; Li et al., 2020). During the early stages of osteoclastogenesis, the binding of RANKL secreted by osteoblasts to the osteoclast precursor cell membrane receptor RANK activates several molecular transduction pathways, including MAPK, NF-κB, PI3K/Akt, and others (Jia et al., 2019). Among these signaling pathways, the PI3K/Akt signaling cascade regulates c-Fos expression during osteoclastogenesis, which in turn regulates osteoclast function (Yeon et al., 2019). Increasing phosphorylation of Akt promoted c-Fos activation significantly (Han and Kim, 2019), while c-Fos knockout mice exhibited significant downregulation of osteoclast marker genes (Acp5, CtsK, etc.) (Fleischmann et al., 2000). Various drugs such as Idelalisib, Asperolide A, Garcinol, and Ebselen have been proven to inhibit osteoclast function via the PI3K/Akt/c-Fos pathway and exert an anti-osteolysis effects (Suh et al., 2013; Baek et al., 2016; Yeon et al., 2019; Jiang et al., 2020).

In this study, we found that ECH could inhibit the PI3K/Akt/c-Fos pathway in osteoclasts by downregulating PI3K expression, decreasing Akt phosphorylation levels, and decreasing c-Fos expression. NFATc1 is a specific transcription factor that regulates osteoclast-specific genes (Acp5, CtsK, etc.) and RANKL/RANK complex-mediated osteoclast differentiation and functional activation (Ahern et al., 2018). The present data indicated that ECH treatment significantly reduced RANKL-induced NFATc1 activation and the expression of downstream target genes Acp5 and CtsK. Therefore, these results confirmed that the inhibitory effects on osteoclast differentiation and anti-osteoclast function of ECH was performed by inhibiting NFATc1 expression through the PI3K/Akt/c-Fos signaling pathway.

However, our study does have limitations. First, although the PJI rat model established in this study is not a classical osteolysis disease model, PJI often occurs in postoperative complications in TJA patients clinically and accurately reflect the pathological changes of inflammatory osteolysis caused by infection. Therefore, the PJI model we used to explore the therapeutic drugs for inflammatory osteolysis is reliable. We plan to address this limitation by subsequently additional preclinical studies in ovariectomy-induced osteoporosis, bone defect, or fracture repair. Second, although previous studies have indicated that ECH has certain anti-inflammatory effects, this study aimed to explore the inhibitory effects of ECH on osteoclast activity and infected osteolysis after debridement in PJI rats. We planned to perform subsequent studies on the combined administration of ECH and antibiotics on infection treatment, inhibition of osteoclasts activities and infected osteolysis.

## 5 Conclusion

**FIGURE 7:**
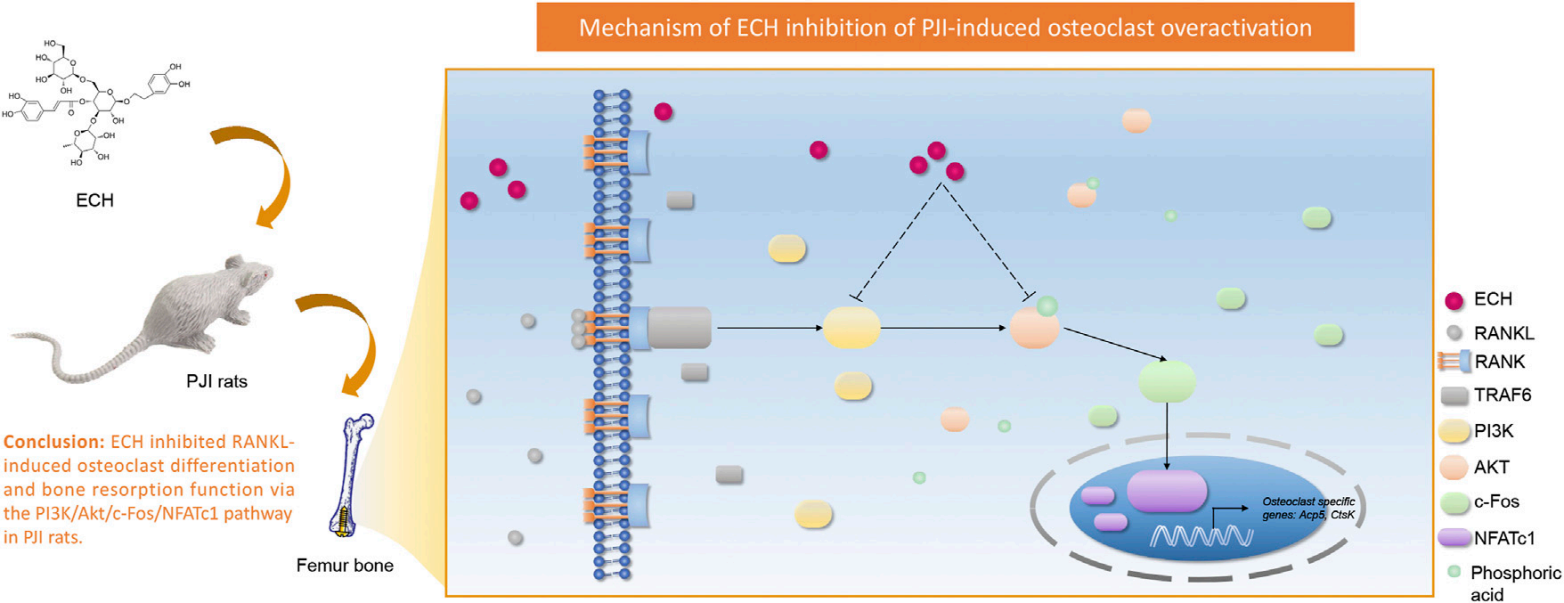
Schematic diagram for the mechanism of ECH inhibition on RANKL-induced osteoclastogenesis.

Our study showed that ECH inhibited RANKL-induced osteoclast differentiation and bone resorption function. The mechanism was that ECH inhibited c-Fos expression by down-regulating PI3K expression and Akt phosphorylation. This effect subsequently down-regulated the expression of the downstream nuclear transcription factor NFATc1 and led to the inhibition of osteoclast marker gene Acp5, CtsK expression. This data suggested that ECH was expected to be an effective therapeutic agent for inflammatory osteolysis (Figure 7).

## Data Availability

The original contributions presented in the study are included in the article/Supplementary Material, further inquiries can be directed to the corresponding author.
