# Supplementary figures and images for: Echinacoside Inhibits Osteoclast Function by Down-Regulating PI3K/Akt/C-Fos to Alleviate Osteolysis Caused by Periprosthetic Joint Infection
Source: Front Pharmacol. 2022 Jun 24;13:930053. doi: 10.3389/fphar.2022.930053 (PMC9263215; doi:10.3389/fphar.2022.930053)

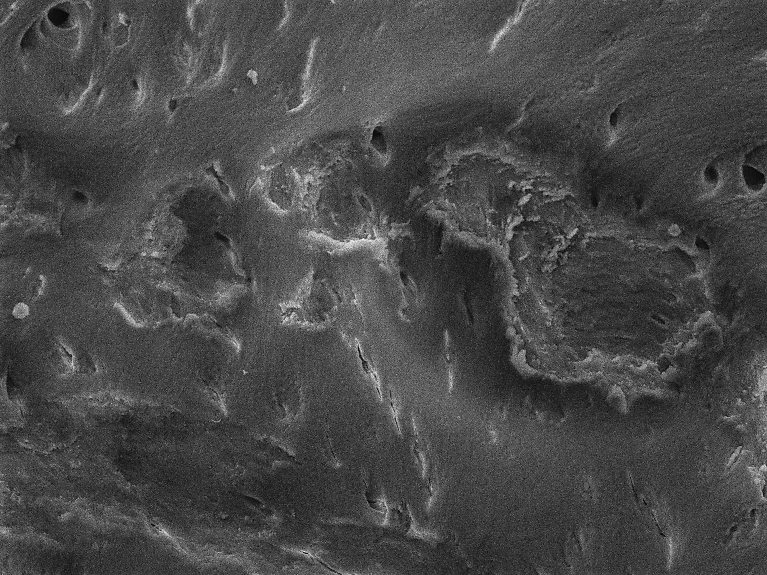

Supplement: Supplementary file 1 [file DataSheet3.ZIP › origin-Osteone(cell)/22.tif]

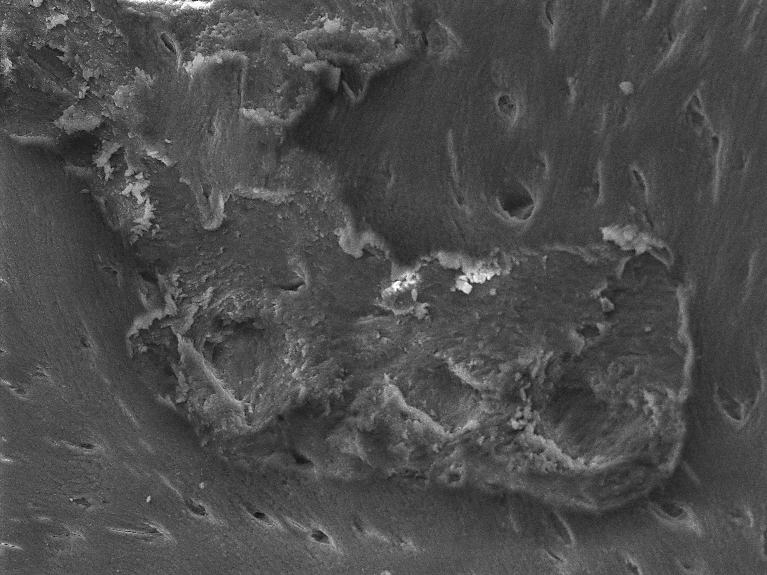

Supplement: Supplementary file 1 [file DataSheet3.ZIP › origin-Osteone(cell)/4.tif]

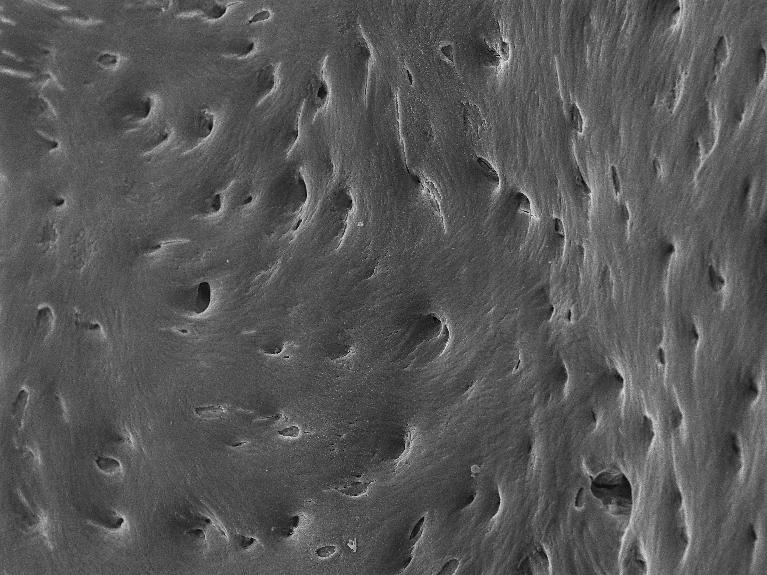

Supplement: Supplementary file 1 [file DataSheet3.ZIP › origin-Osteone(cell)/6.tif]

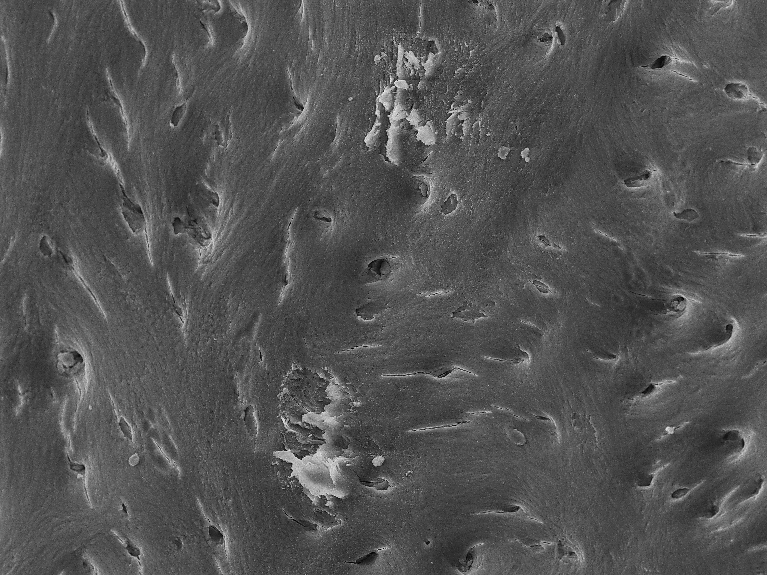

Supplement: Supplementary file 1 [file DataSheet3.ZIP › origin-Osteone(cell)/7.tif]

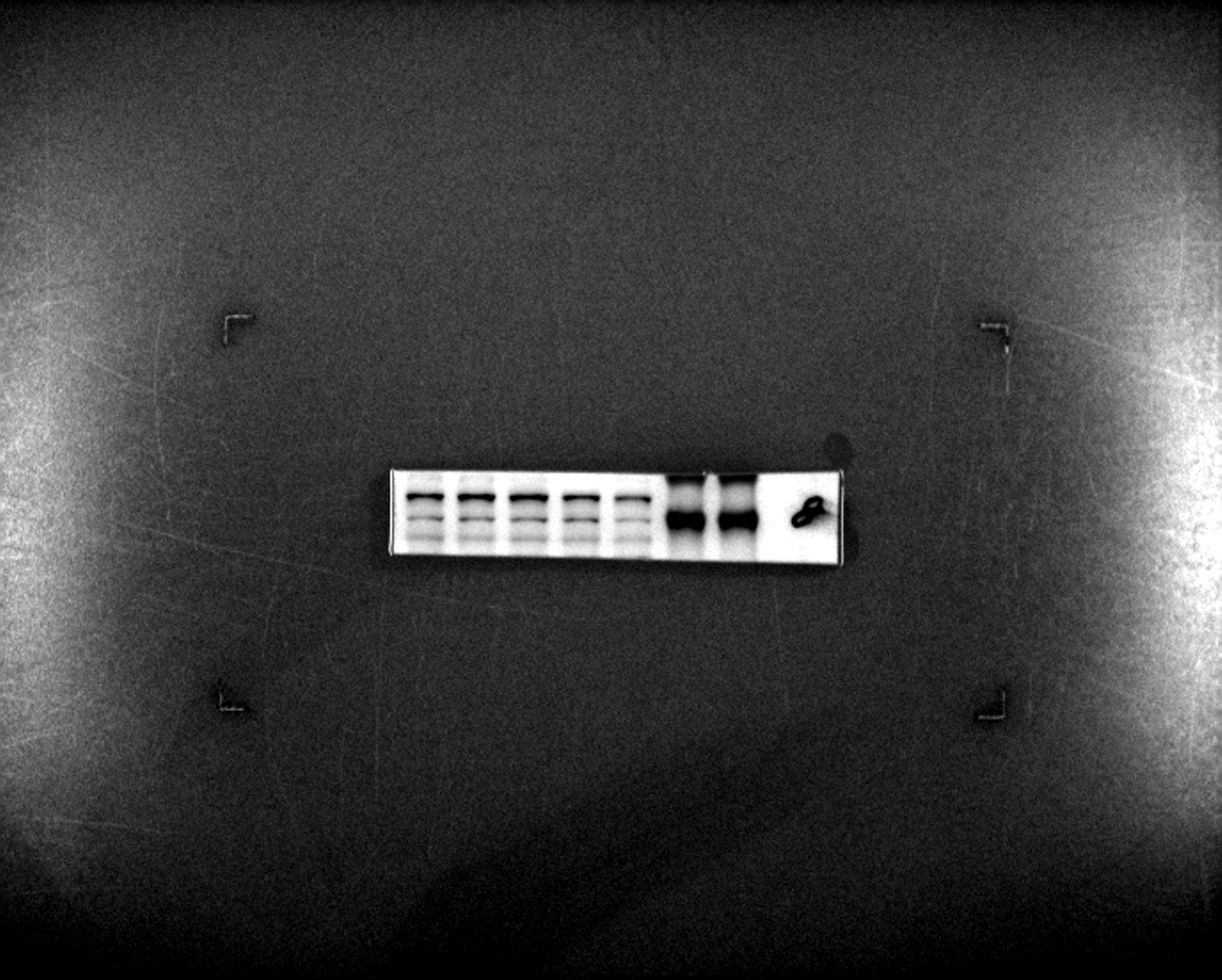

Supplement: Supplementary file 2 [file DataSheet9.ZIP › Origin-WB(cell)-Revised/AKT-origin.tif]

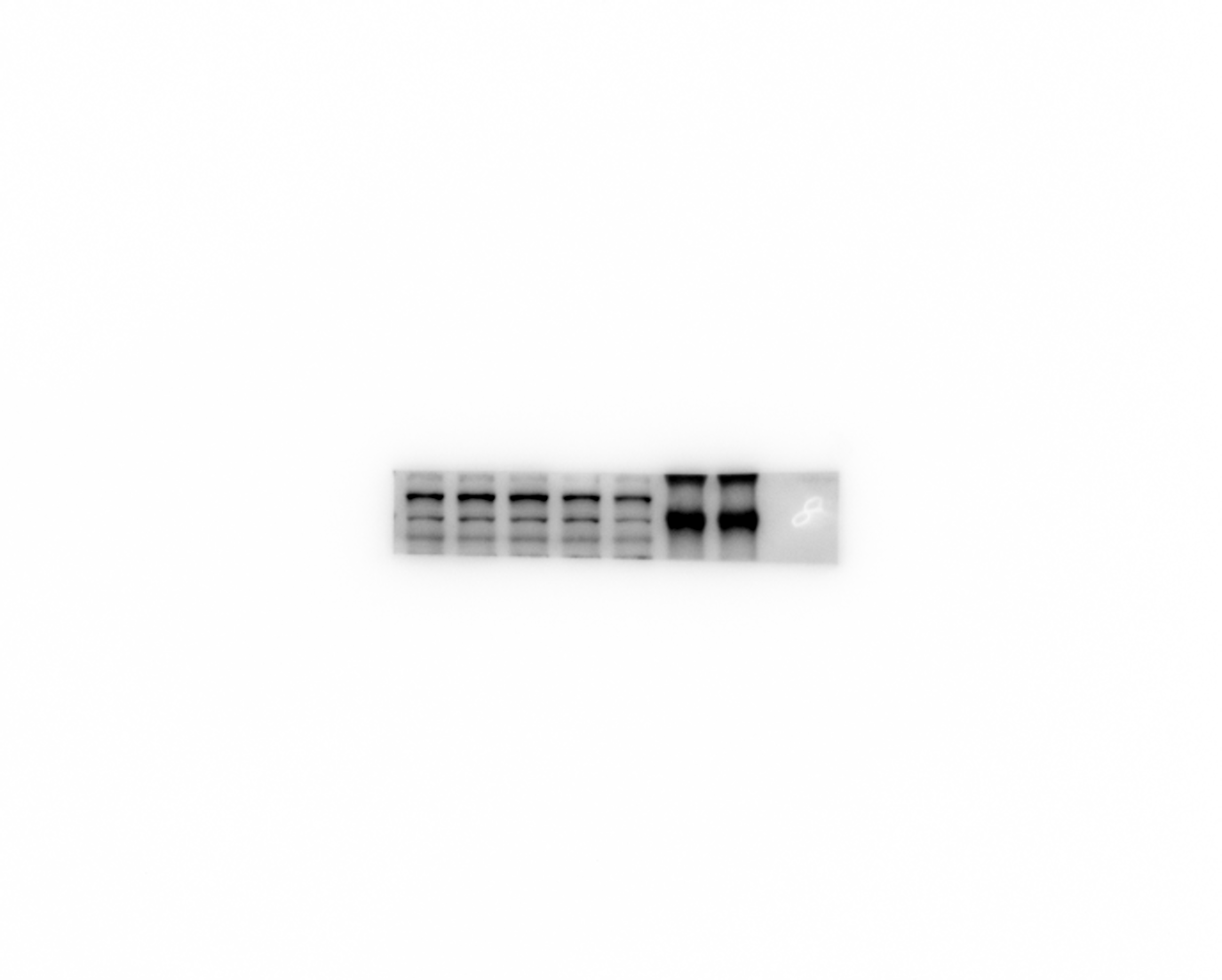

Supplement: Supplementary file 2 [file DataSheet9.ZIP › Origin-WB(cell)-Revised/AKT.tif]

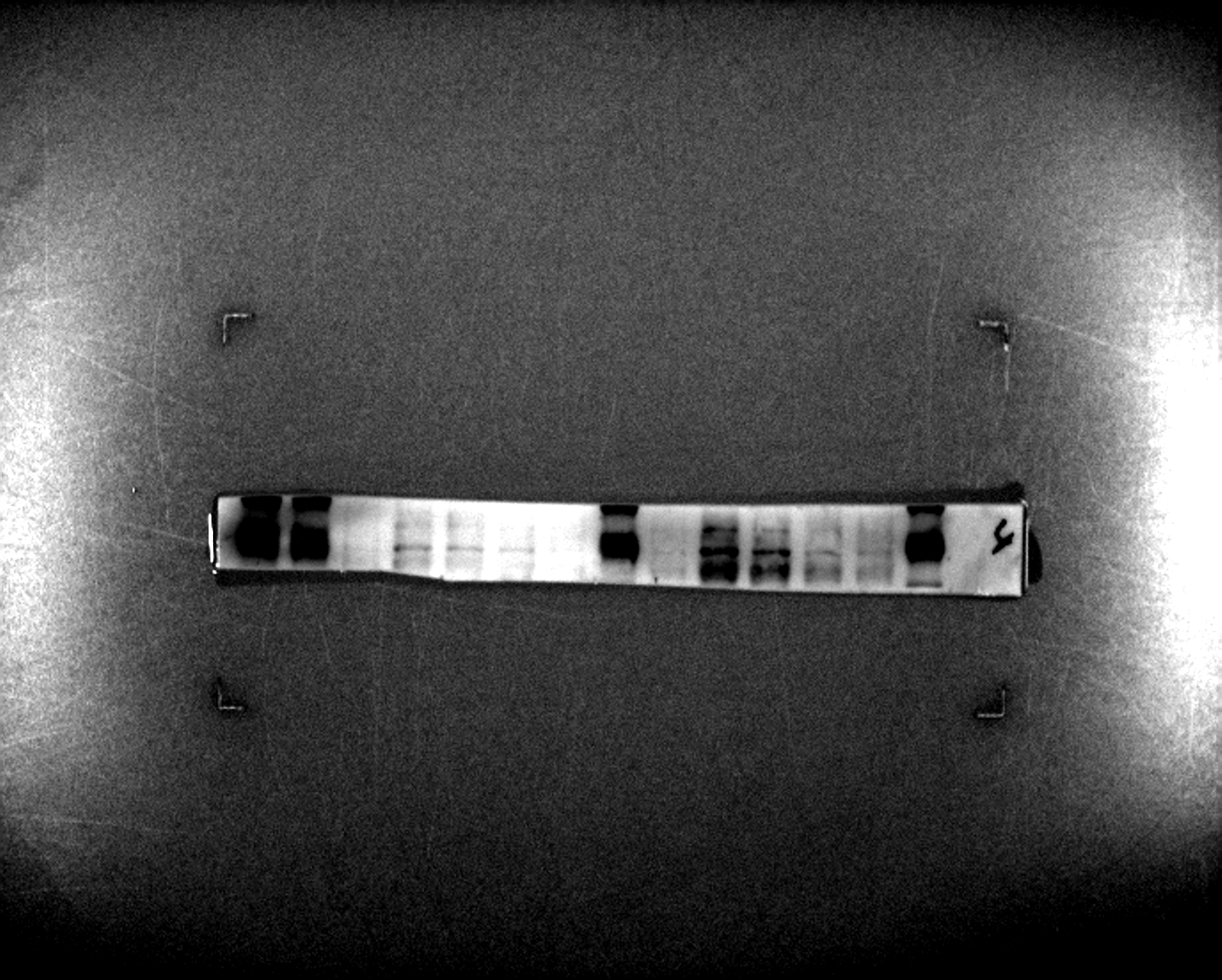

Supplement: Supplementary file 2 [file DataSheet9.ZIP › Origin-WB(cell)-Revised/C-FOS-origin.Tif]

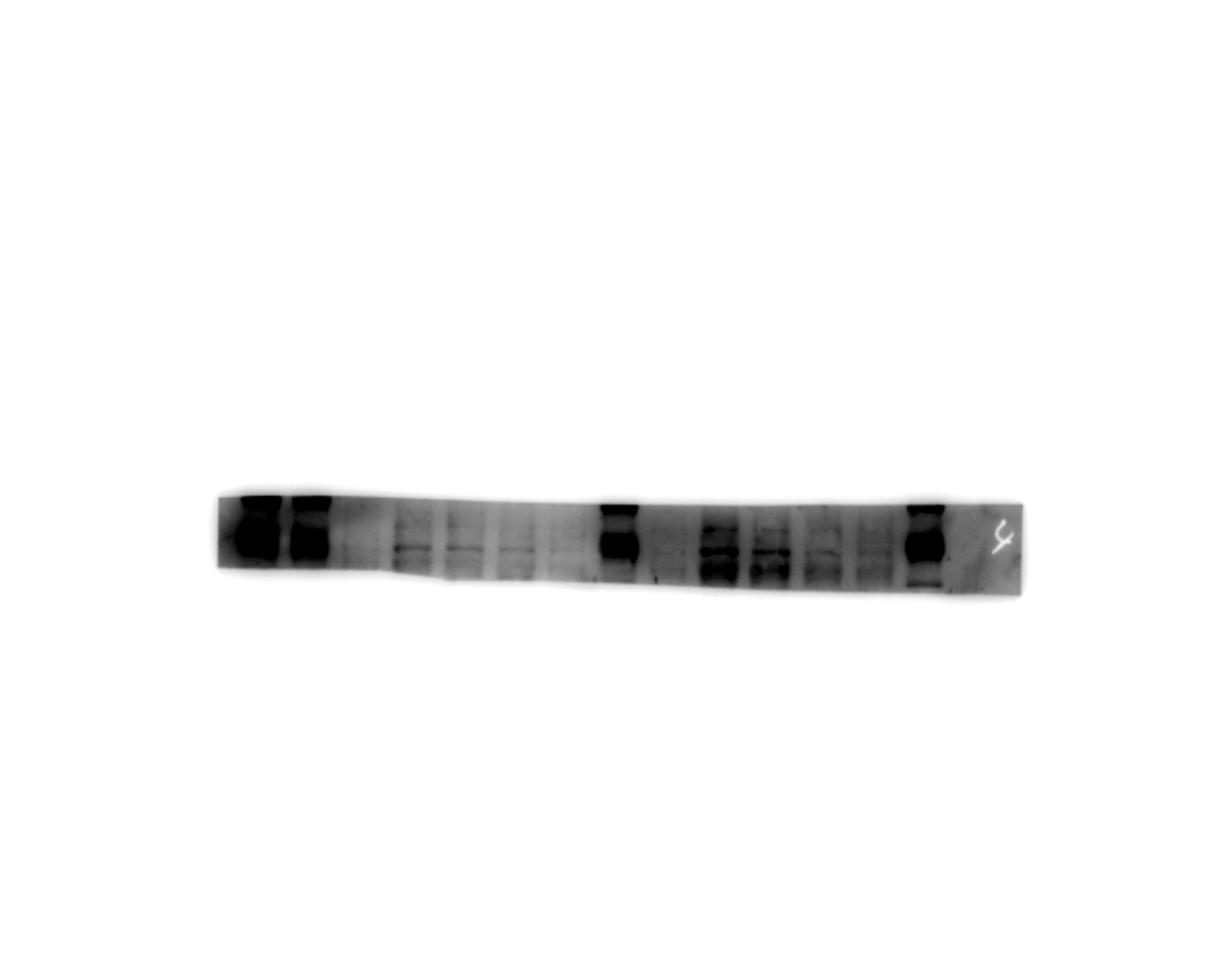

Supplement: Supplementary file 2 [file DataSheet9.ZIP › Origin-WB(cell)-Revised/C-FOS.Tif]

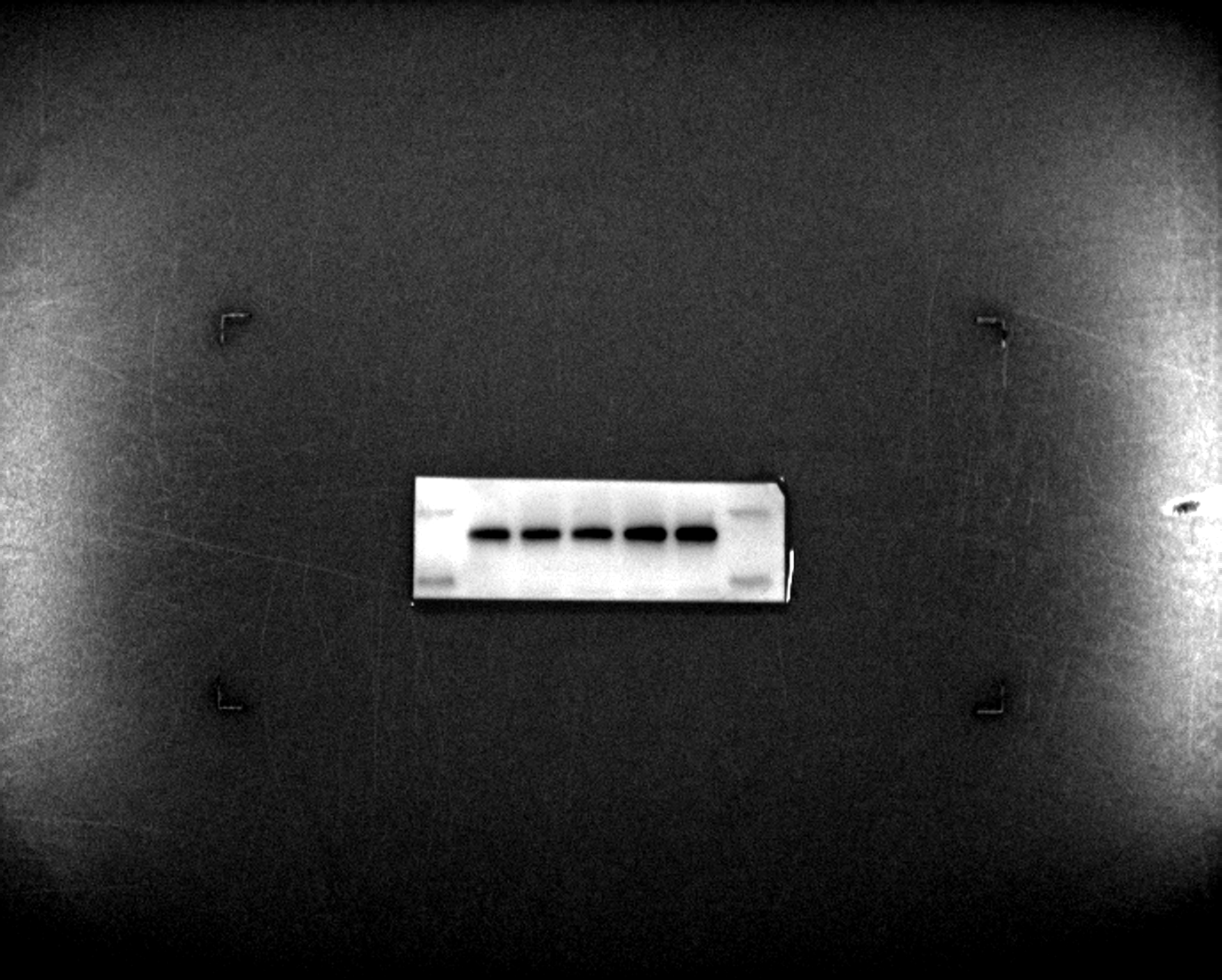

Supplement: Supplementary file 2 [file DataSheet9.ZIP › Origin-WB(cell)-Revised/GAPDH-origin.Tif]

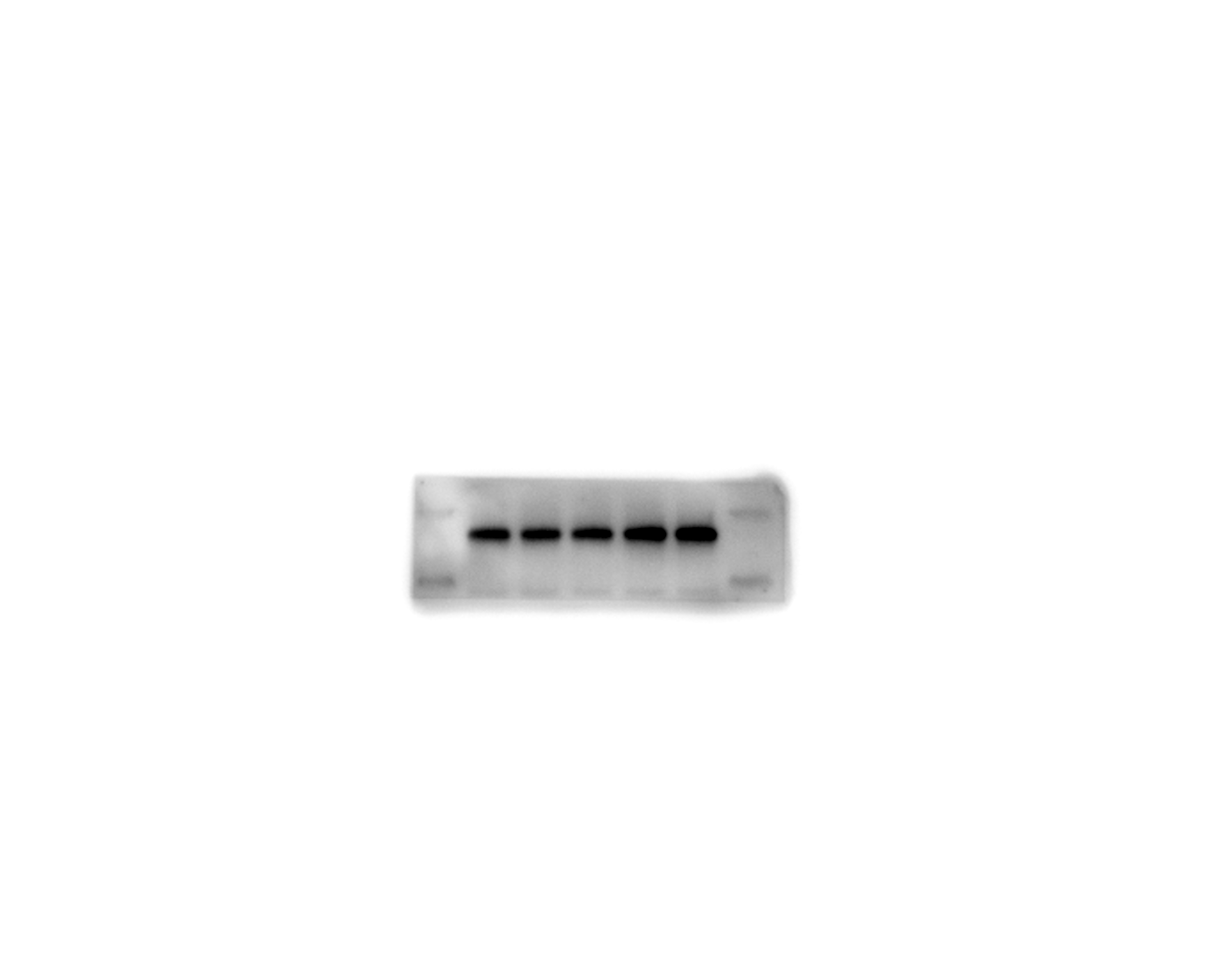

Supplement: Supplementary file 2 [file DataSheet9.ZIP › Origin-WB(cell)-Revised/GAPDH.Tif]

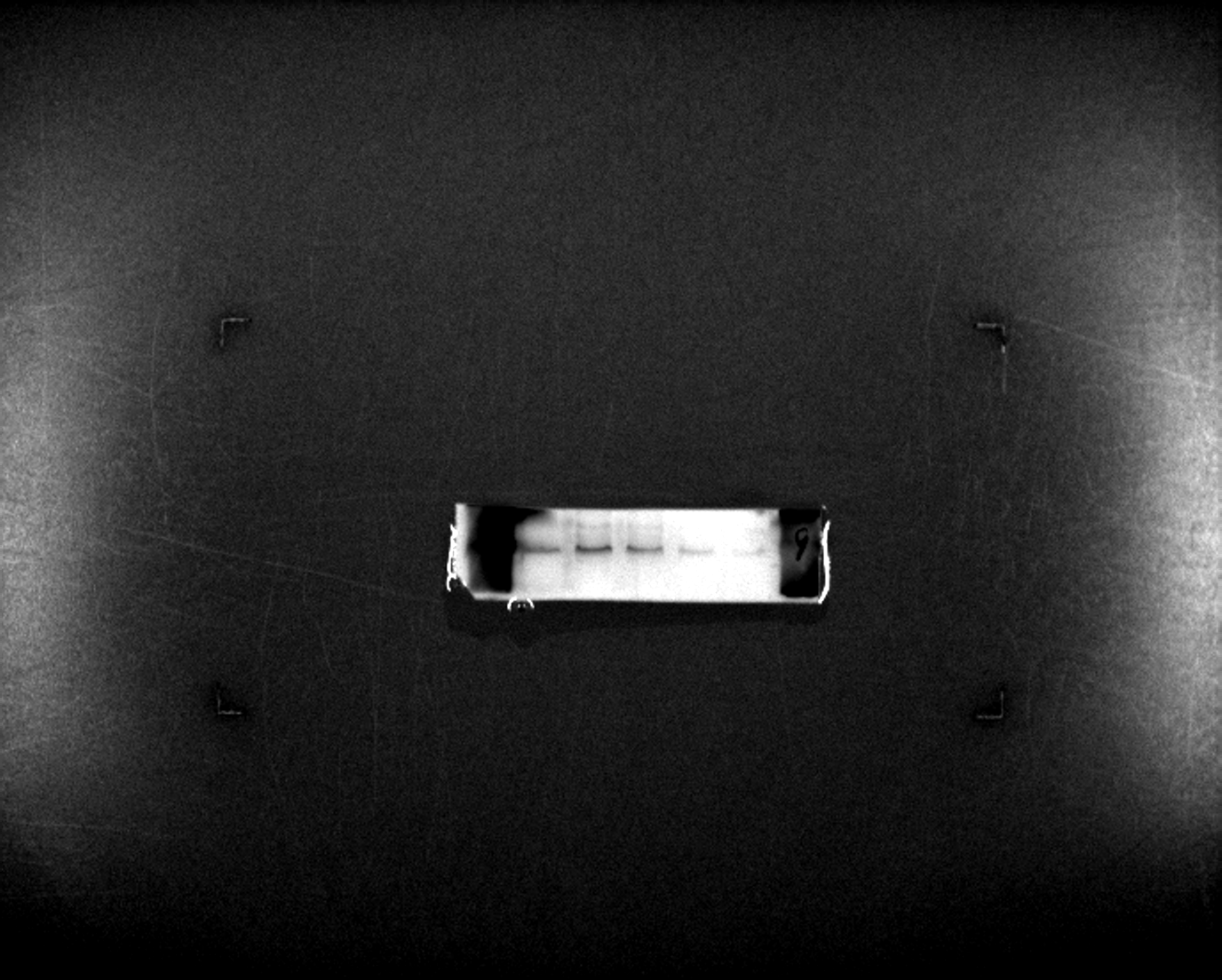

Supplement: Supplementary file 2 [file DataSheet9.ZIP › Origin-WB(cell)-Revised/NFATC1-origin.tif]

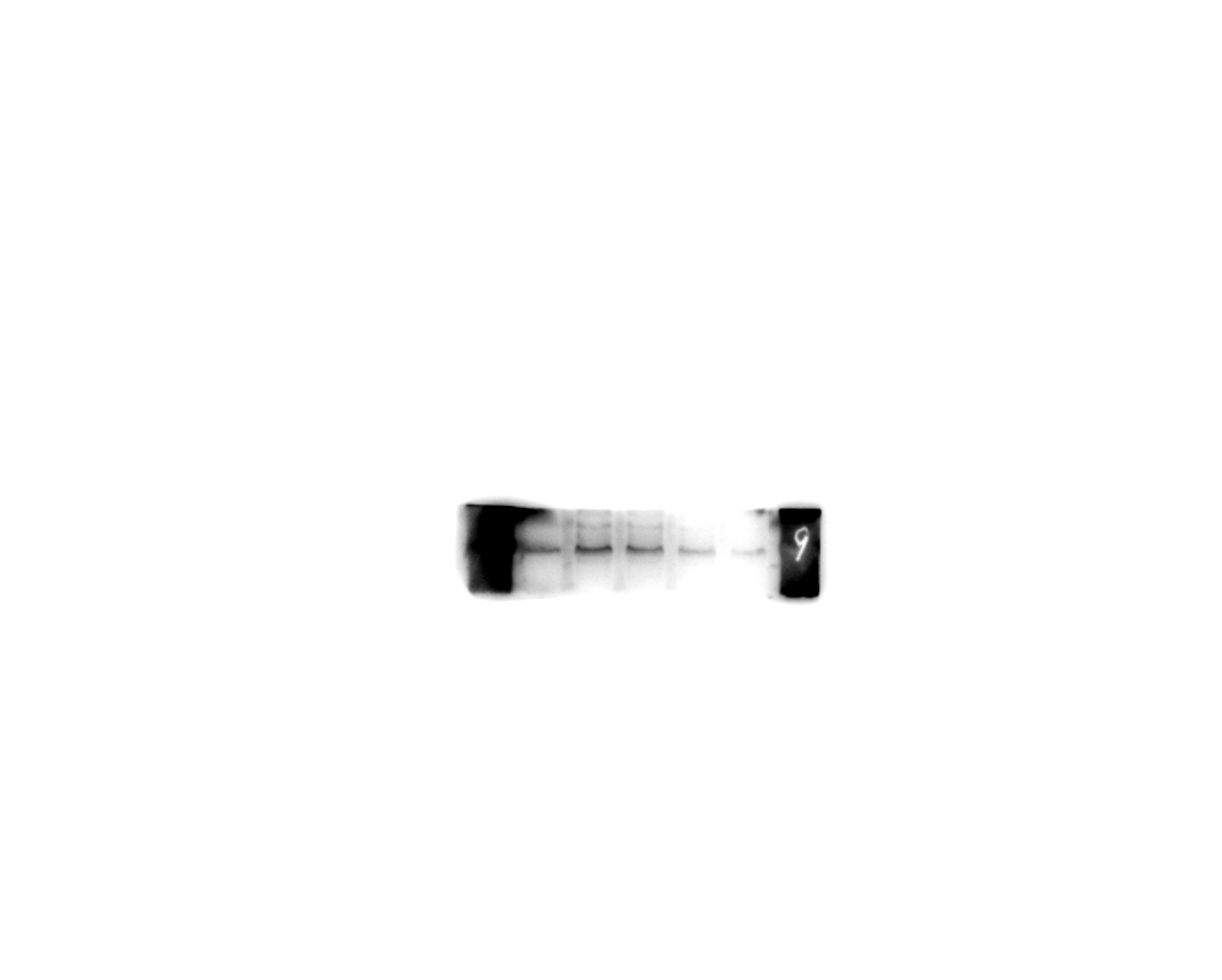

Supplement: Supplementary file 2 [file DataSheet9.ZIP › Origin-WB(cell)-Revised/NFATC1.tif]

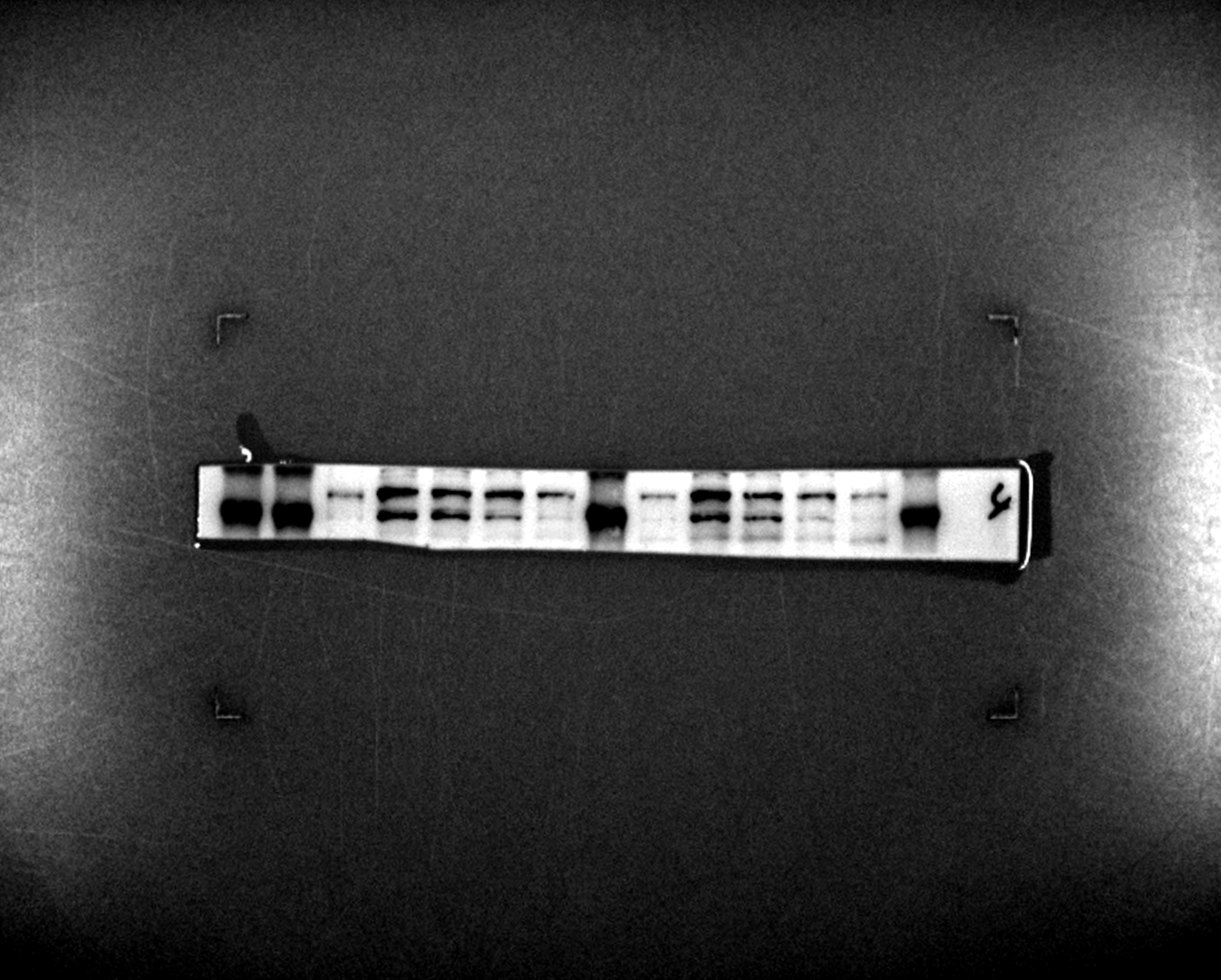

Supplement: Supplementary file 2 [file DataSheet9.ZIP › Origin-WB(cell)-Revised/P-AKT-origin.tif]

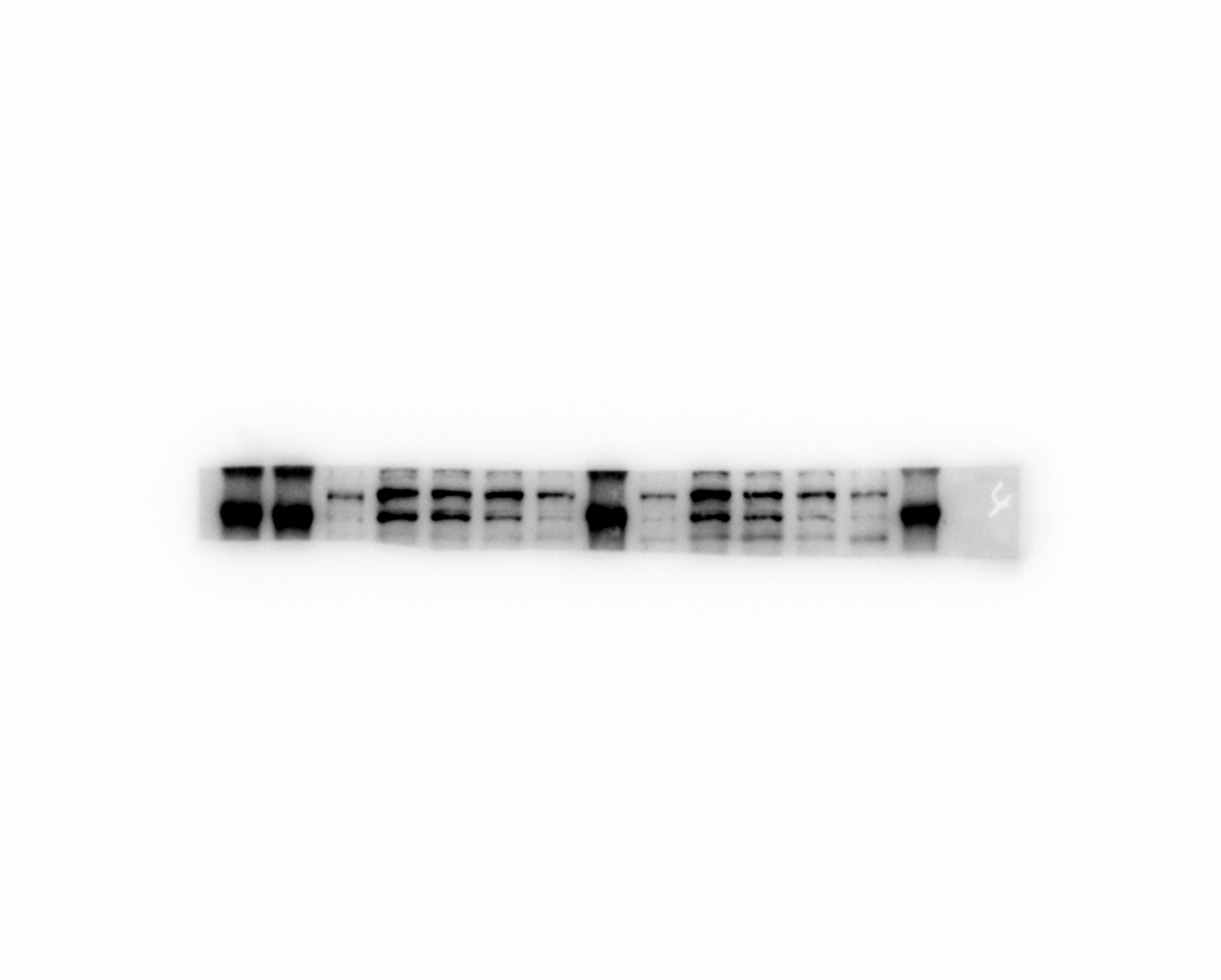

Supplement: Supplementary file 2 [file DataSheet9.ZIP › Origin-WB(cell)-Revised/P-AKT.tif]

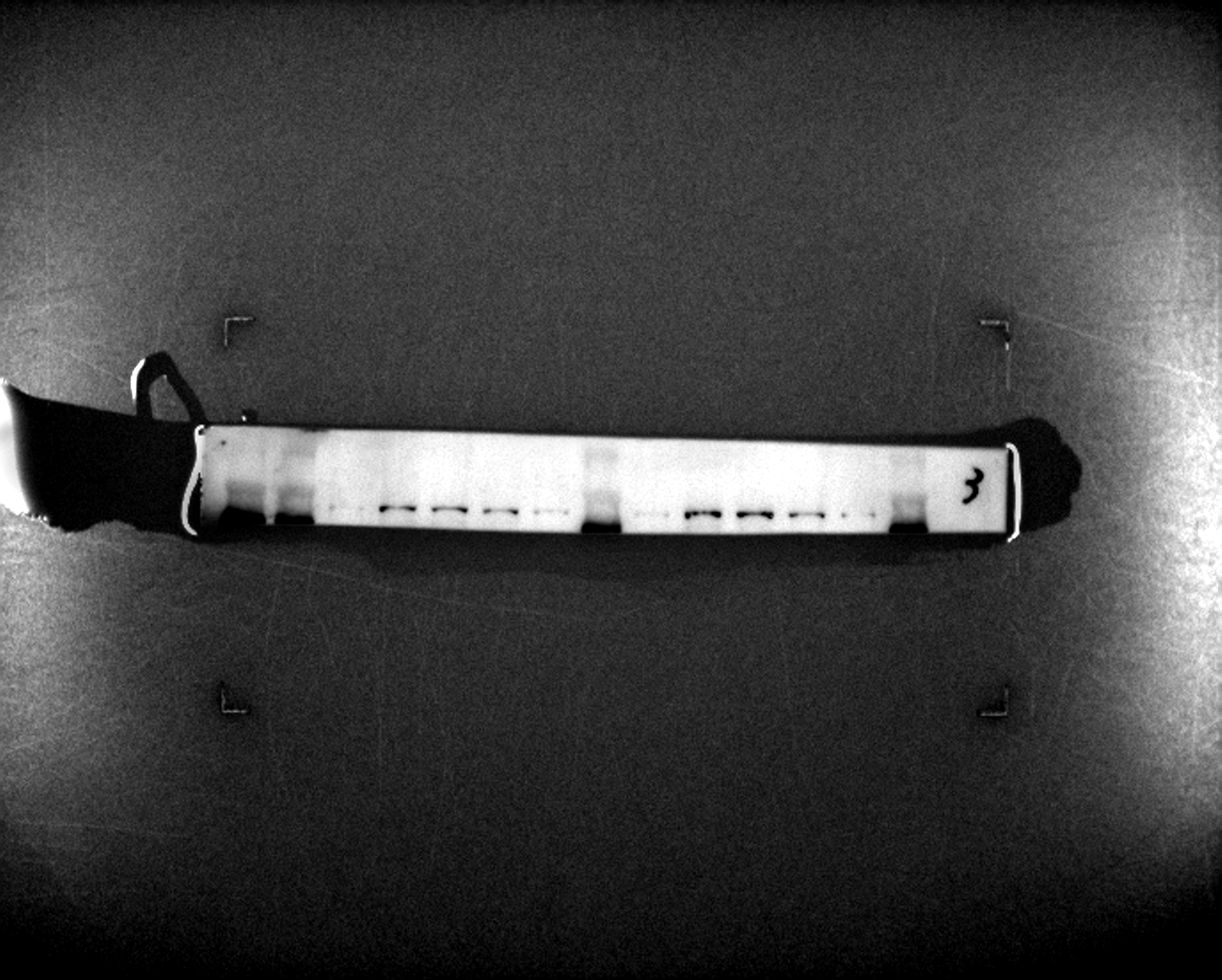

Supplement: Supplementary file 2 [file DataSheet9.ZIP › Origin-WB(cell)-Revised/PI3K-origin.tif]

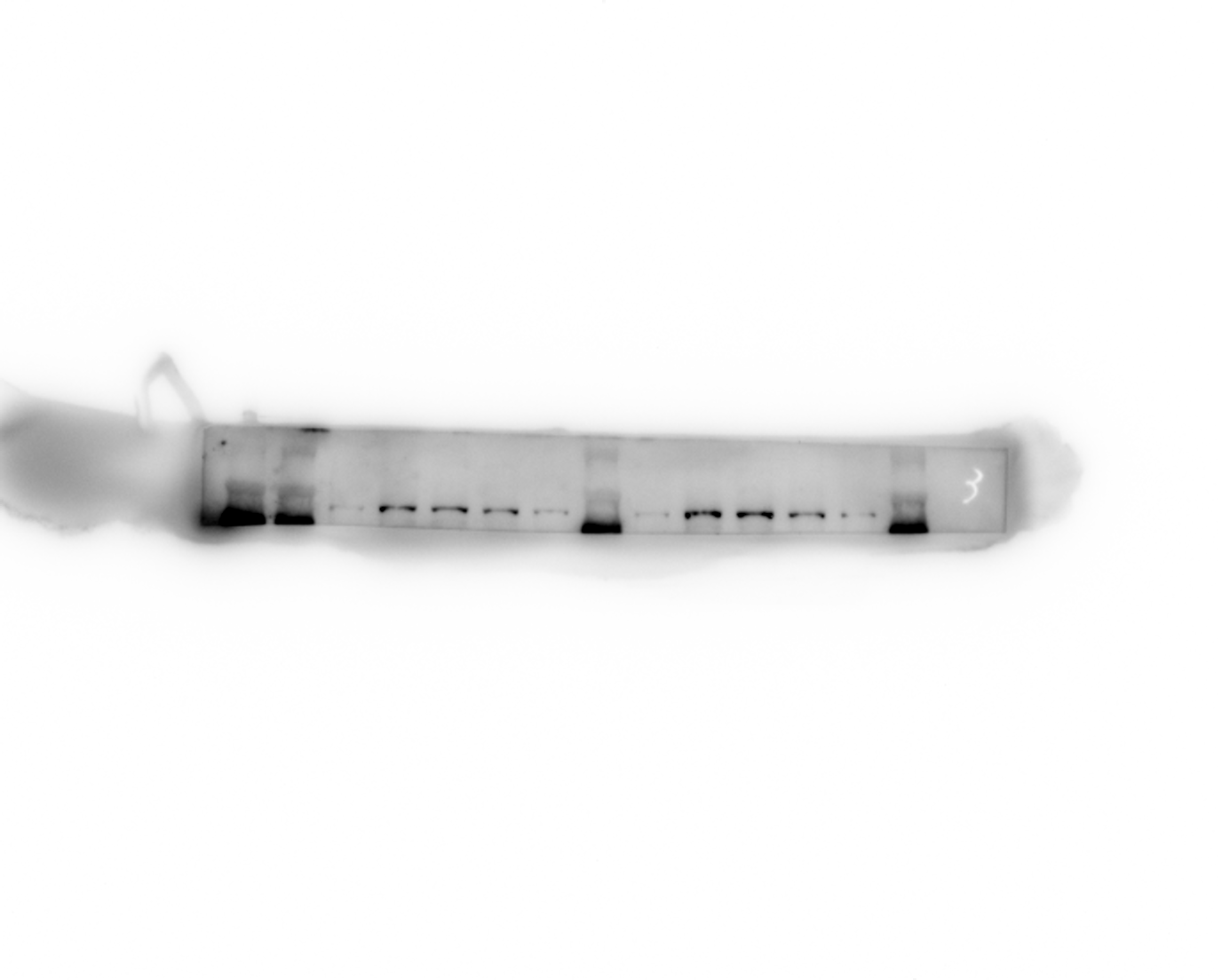

Supplement: Supplementary file 2 [file DataSheet9.ZIP › Origin-WB(cell)-Revised/PI3K.tif]

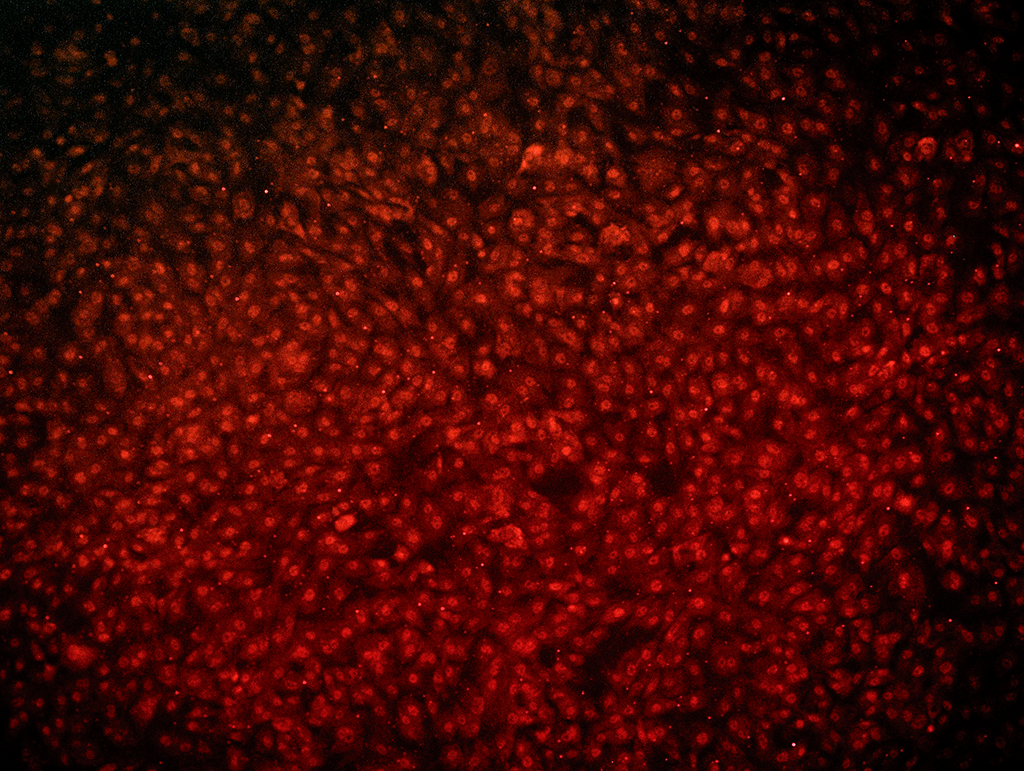

Supplement: Supplementary file 3 [file DataSheet4.ZIP › origin-IF(cell)/ECH-0 μM-c-Fos-100X.tif]

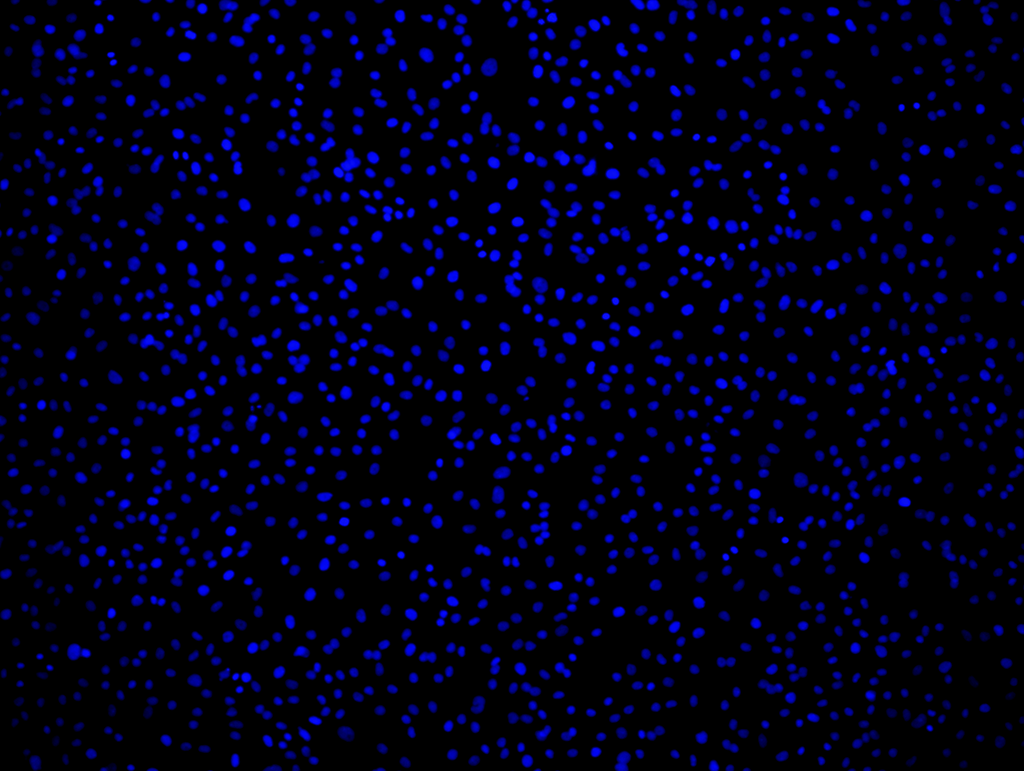

Supplement: Supplementary file 3 [file DataSheet4.ZIP › origin-IF(cell)/ECH-0 μM-DAPI-100X.tif]

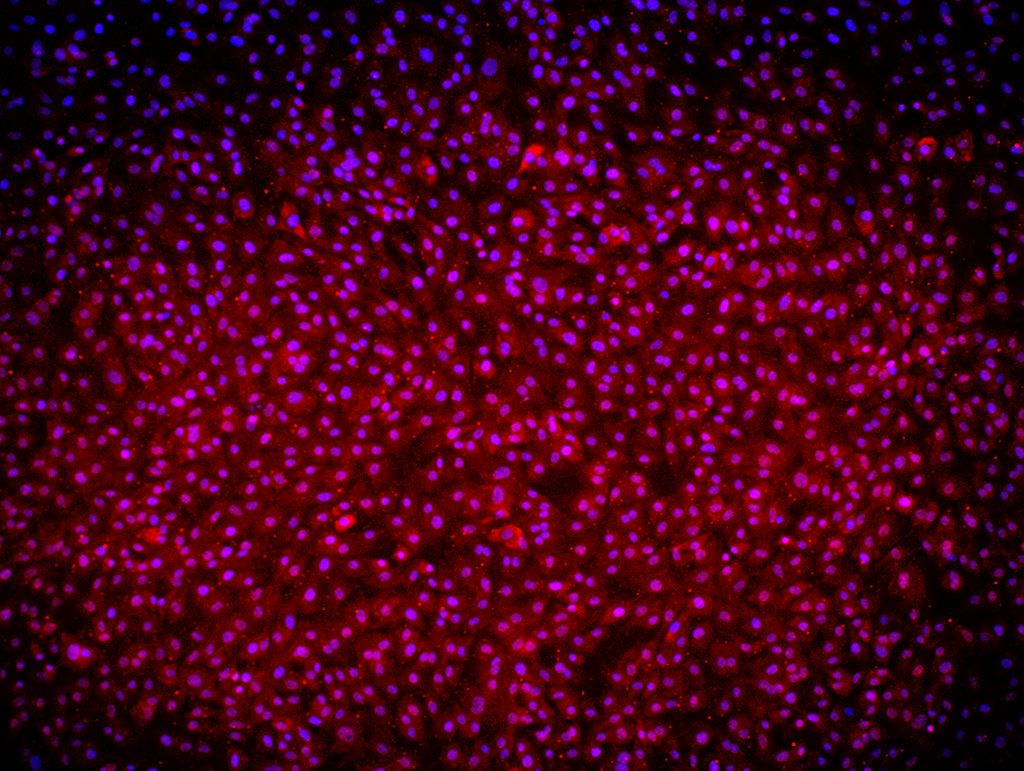

Supplement: Supplementary file 3 [file DataSheet4.ZIP › origin-IF(cell)/ECH-0 μM-merge-100X.tif]

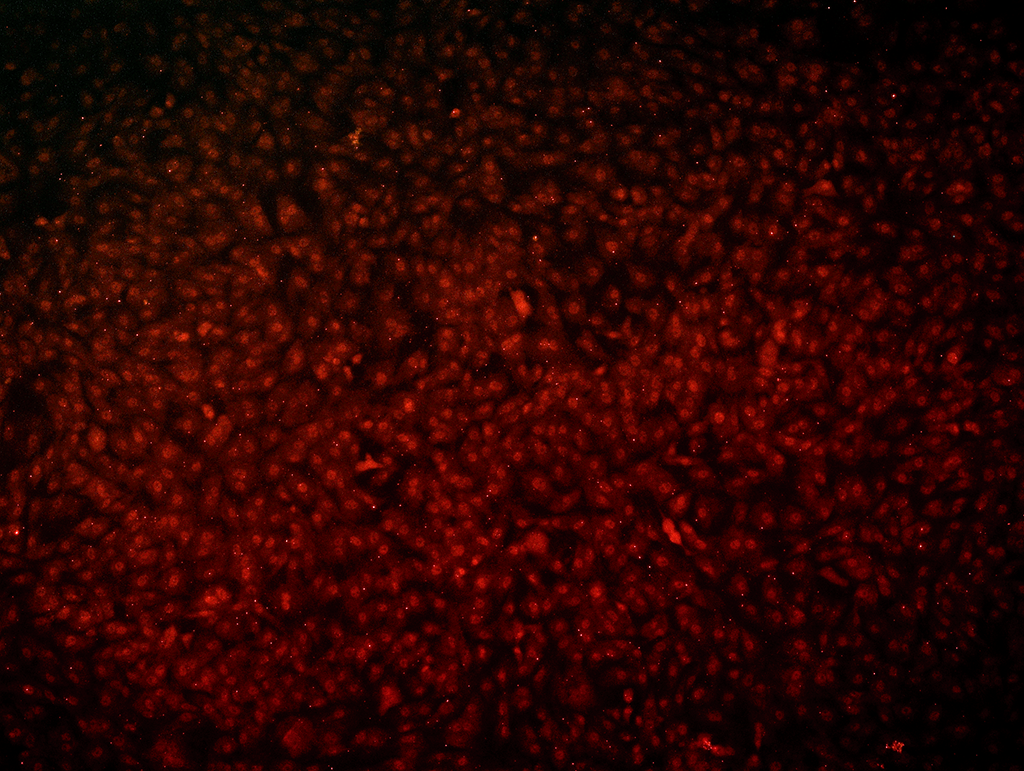

Supplement: Supplementary file 3 [file DataSheet4.ZIP › origin-IF(cell)/ECH-0.2 μM-c-Fos-100X.tif]

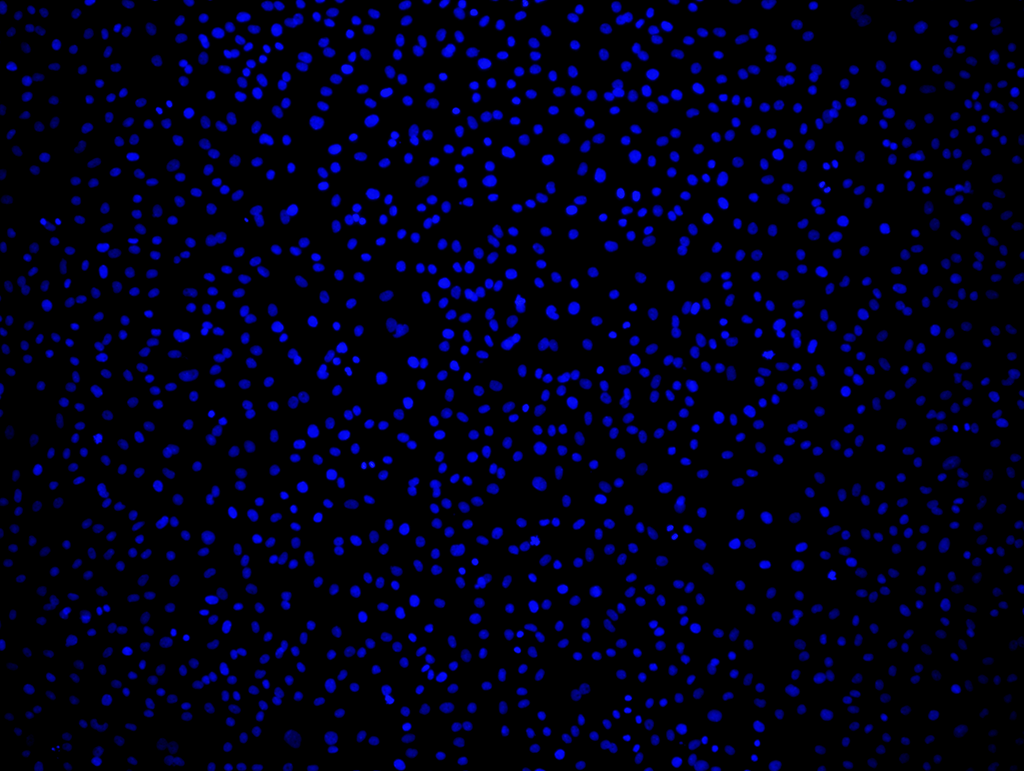

Supplement: Supplementary file 3 [file DataSheet4.ZIP › origin-IF(cell)/ECH-0.2 μM-DAPI-100X.tif]

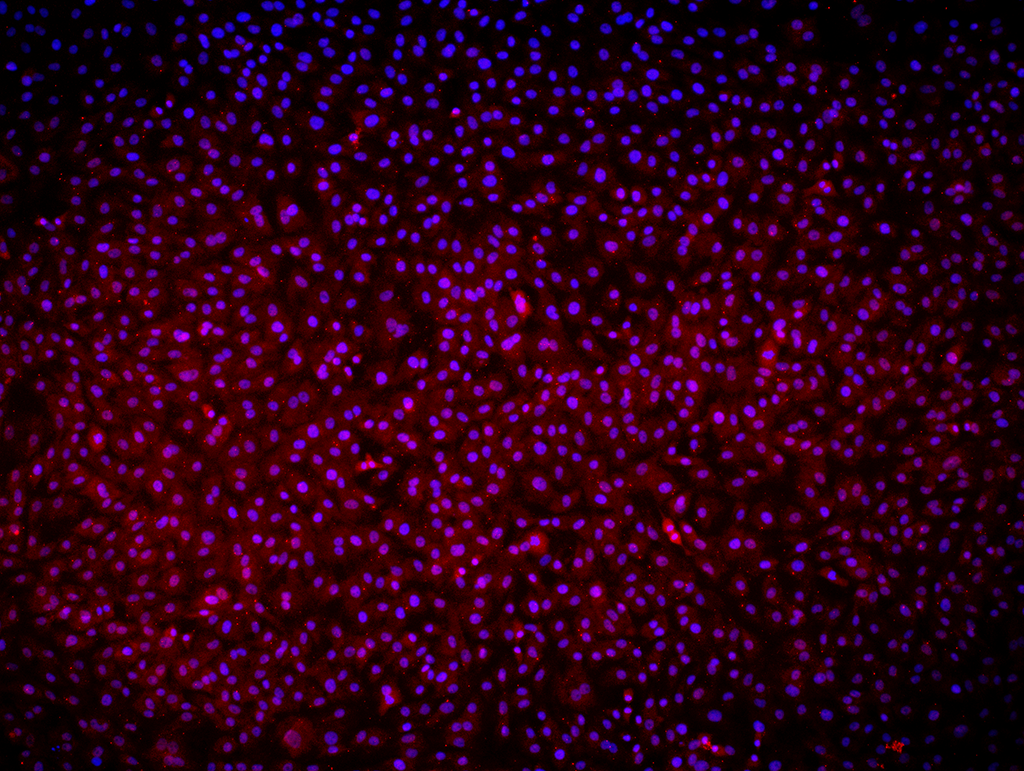

Supplement: Supplementary file 3 [file DataSheet4.ZIP › origin-IF(cell)/ECH-0.2 μM-merge-100X.tif]

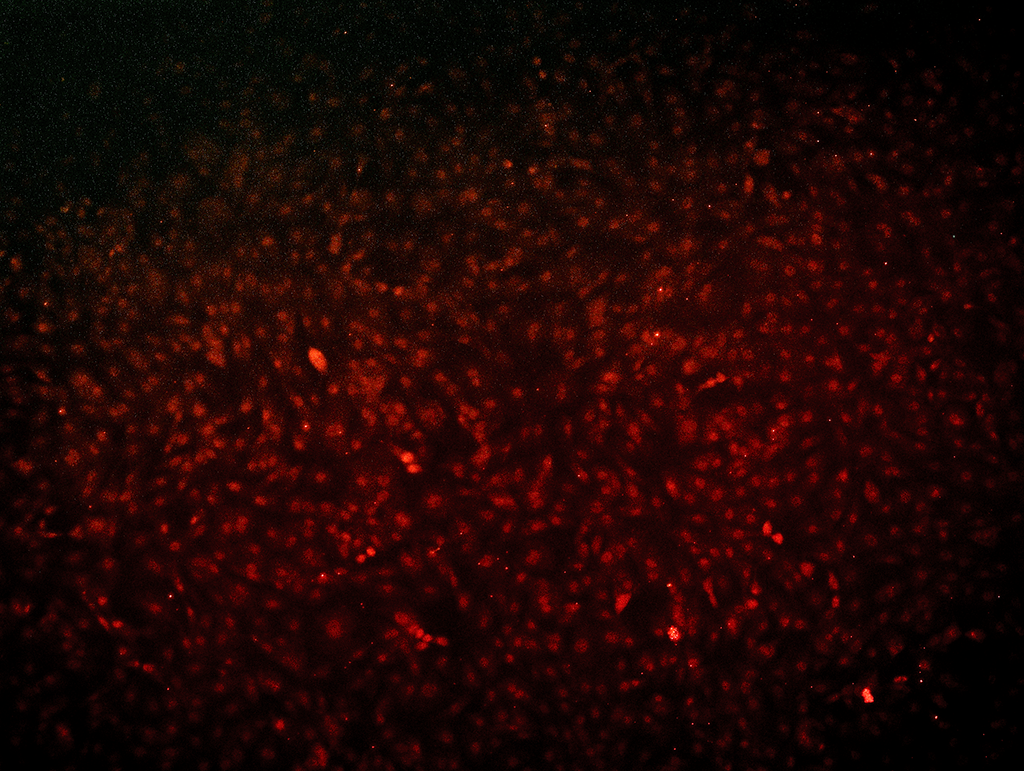

Supplement: Supplementary file 3 [file DataSheet4.ZIP › origin-IF(cell)/ECH-1 μM-c-Fos-100X.tif]

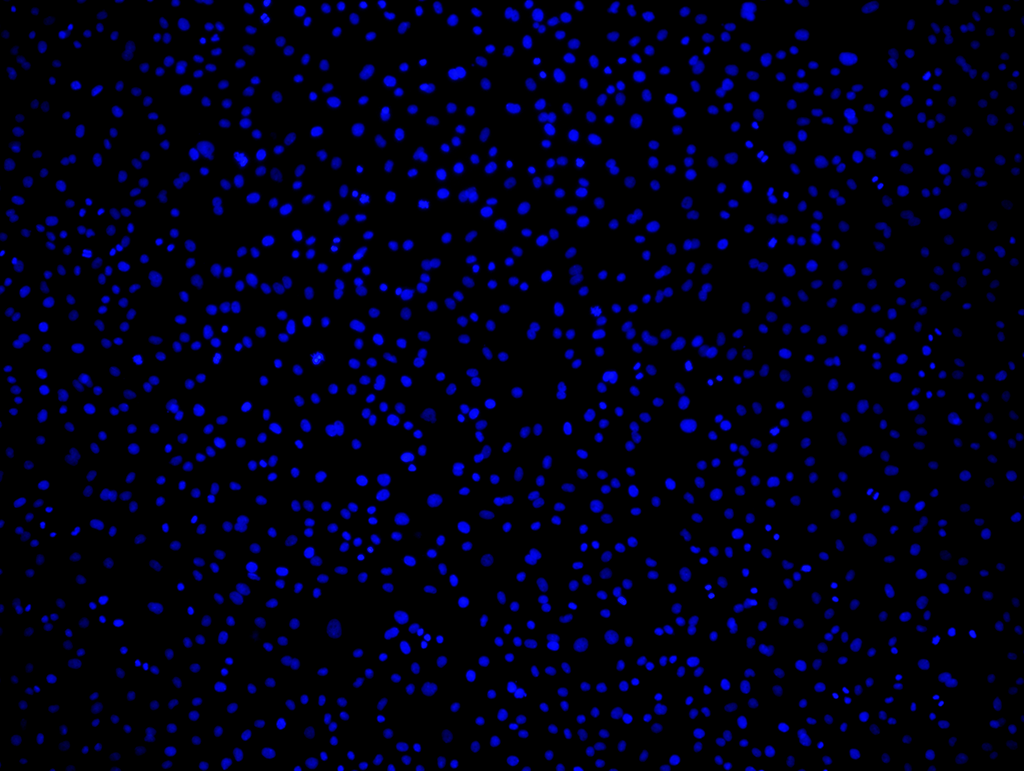

Supplement: Supplementary file 3 [file DataSheet4.ZIP › origin-IF(cell)/ECH-1 μM-DAPI-100X.tif]

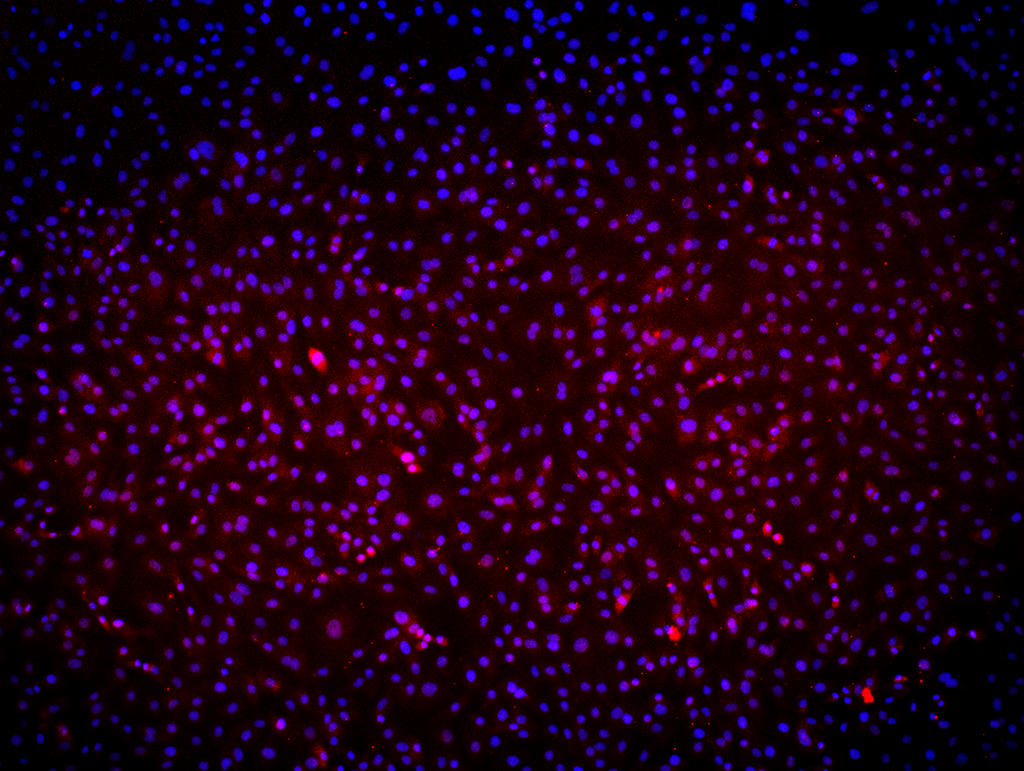

Supplement: Supplementary file 3 [file DataSheet4.ZIP › origin-IF(cell)/ECH-1 μM-merge-100X.tif]

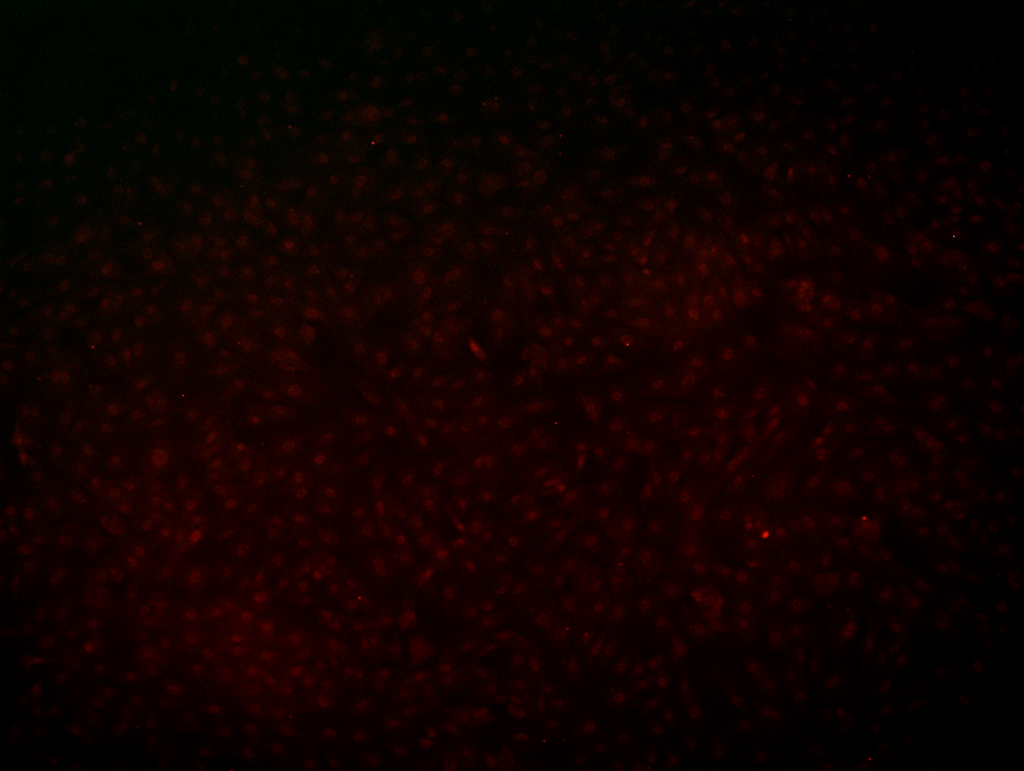

Supplement: Supplementary file 3 [file DataSheet4.ZIP › origin-IF(cell)/ECH-5 μM-c-Fos-100X.tif]

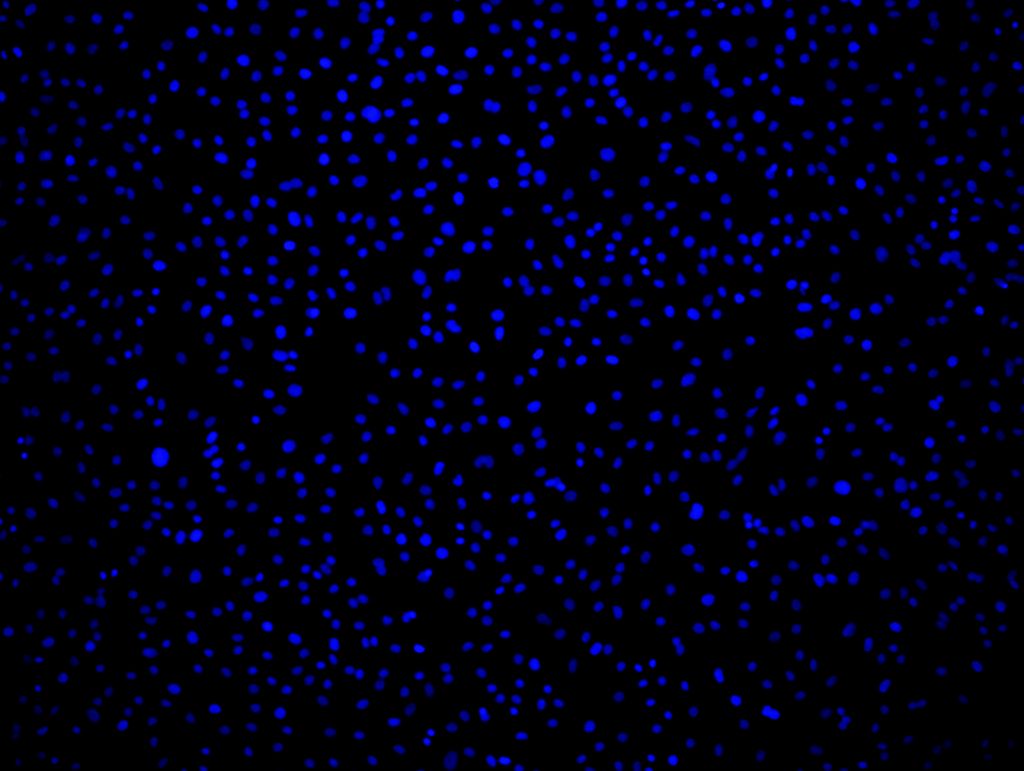

Supplement: Supplementary file 3 [file DataSheet4.ZIP › origin-IF(cell)/ECH-5 μM-DAPI-100X.tif]

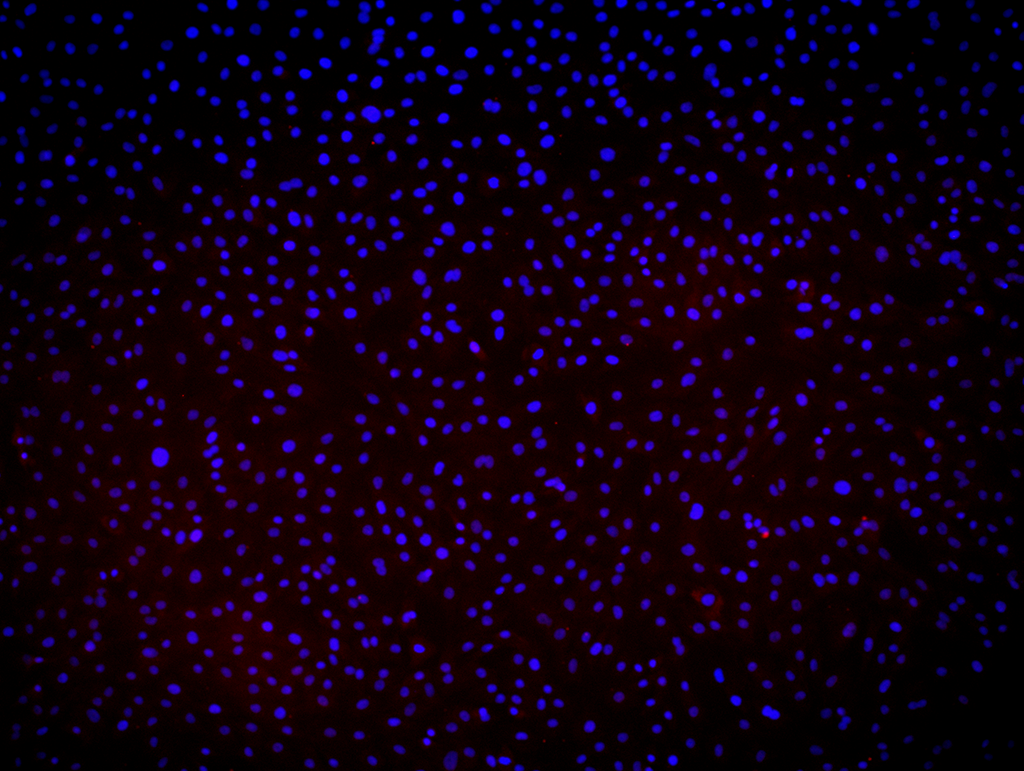

Supplement: Supplementary file 3 [file DataSheet4.ZIP › origin-IF(cell)/ECH-5 μM-merge-100X.tif]

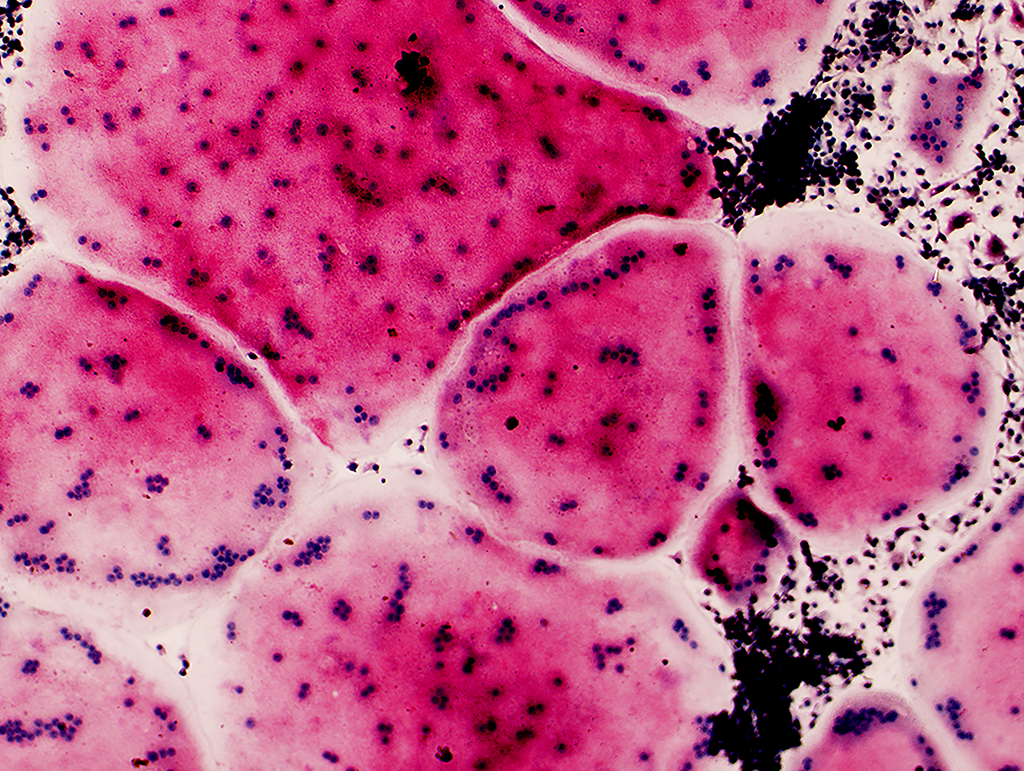

Supplement: Supplementary file 4 [file DataSheet1.ZIP › origin-TRAP(cell)/ECH-0 μM.tif]

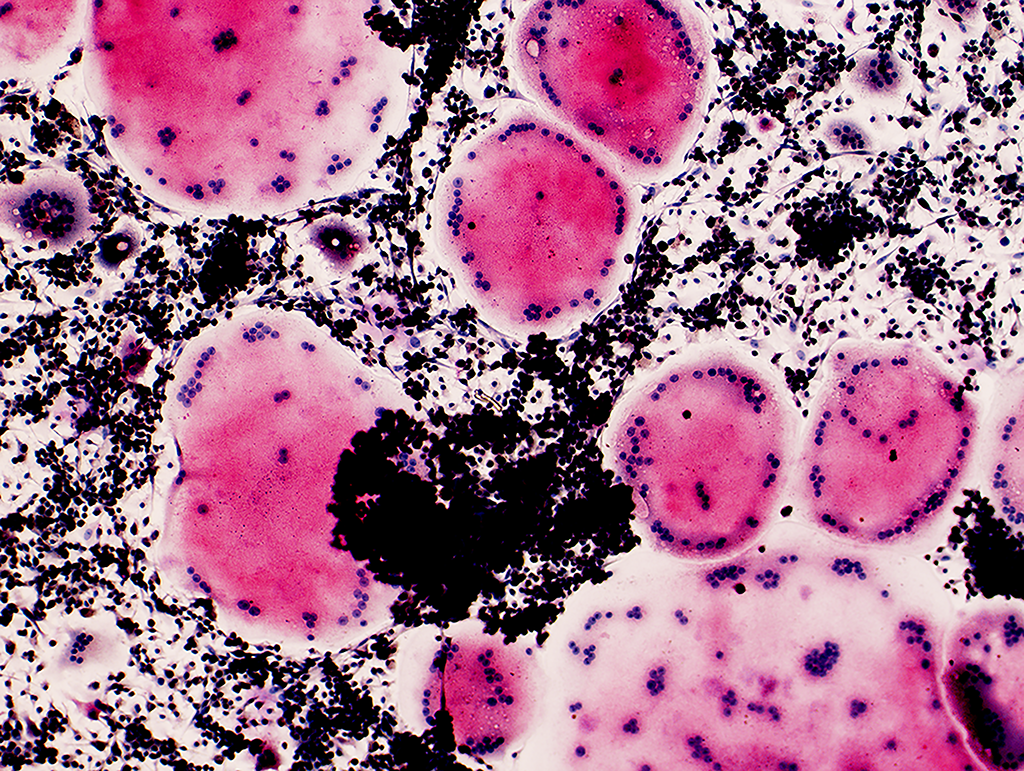

Supplement: Supplementary file 4 [file DataSheet1.ZIP › origin-TRAP(cell)/ECH-0.2 μM.tif]

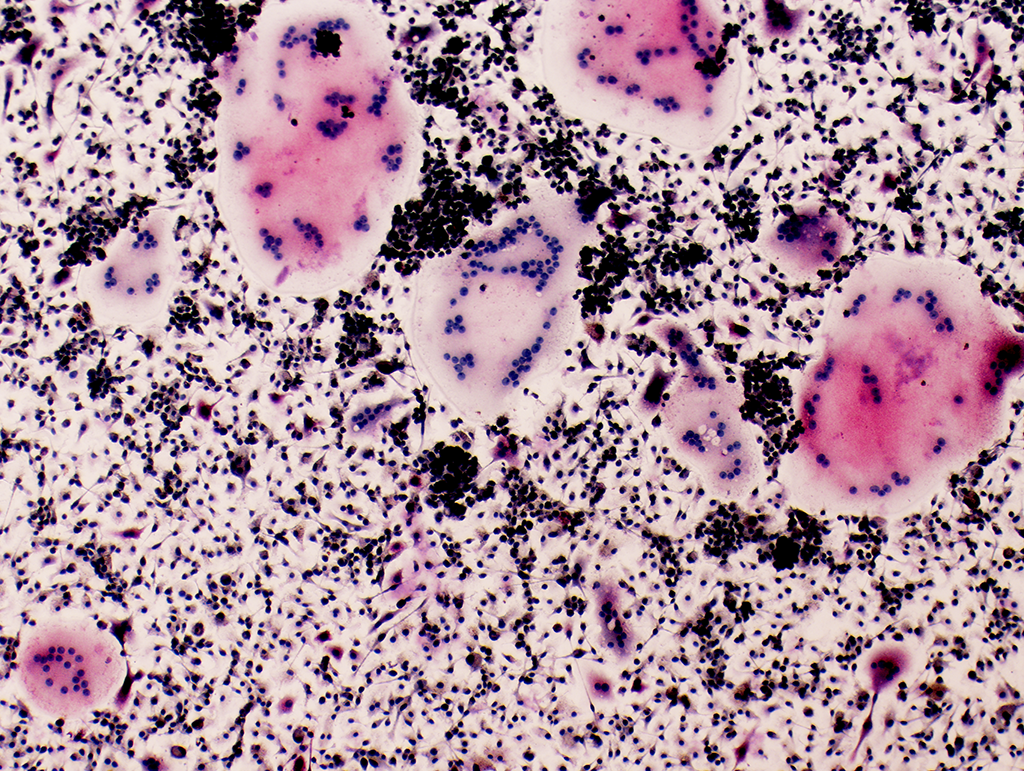

Supplement: Supplementary file 4 [file DataSheet1.ZIP › origin-TRAP(cell)/ECH-1 μM.tif]

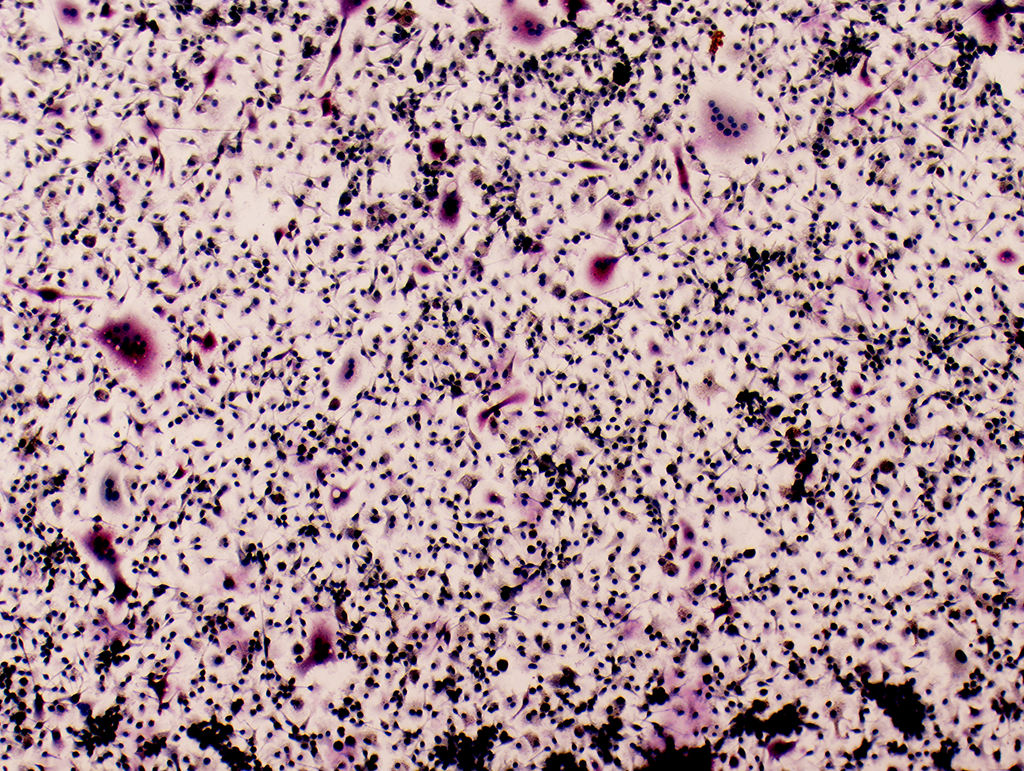

Supplement: Supplementary file 4 [file DataSheet1.ZIP › origin-TRAP(cell)/ECH-5 μM.tif]

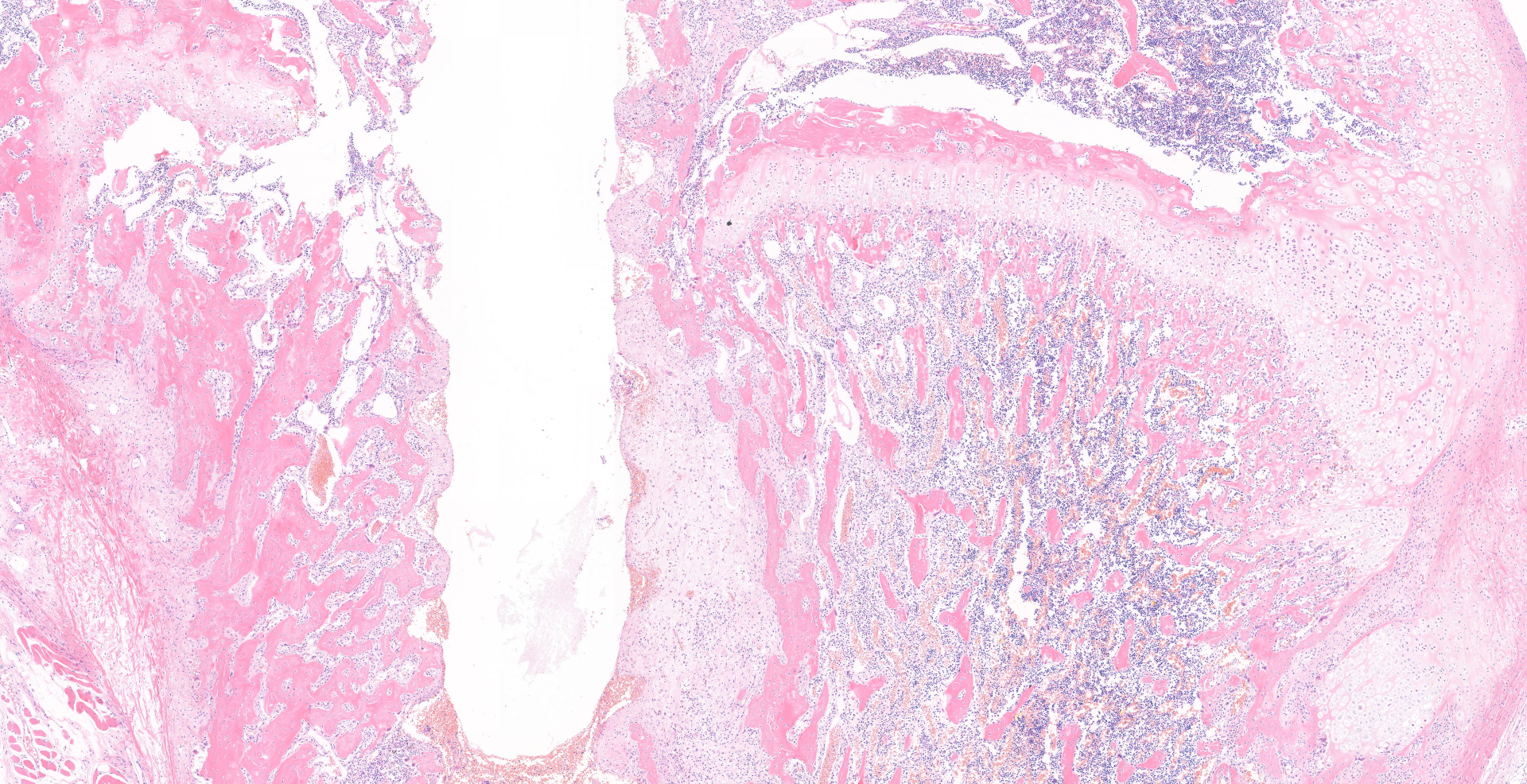

Supplement: Supplementary file 5 [file DataSheet6.ZIP › origin-HE(animal)/CON-20X.tif]

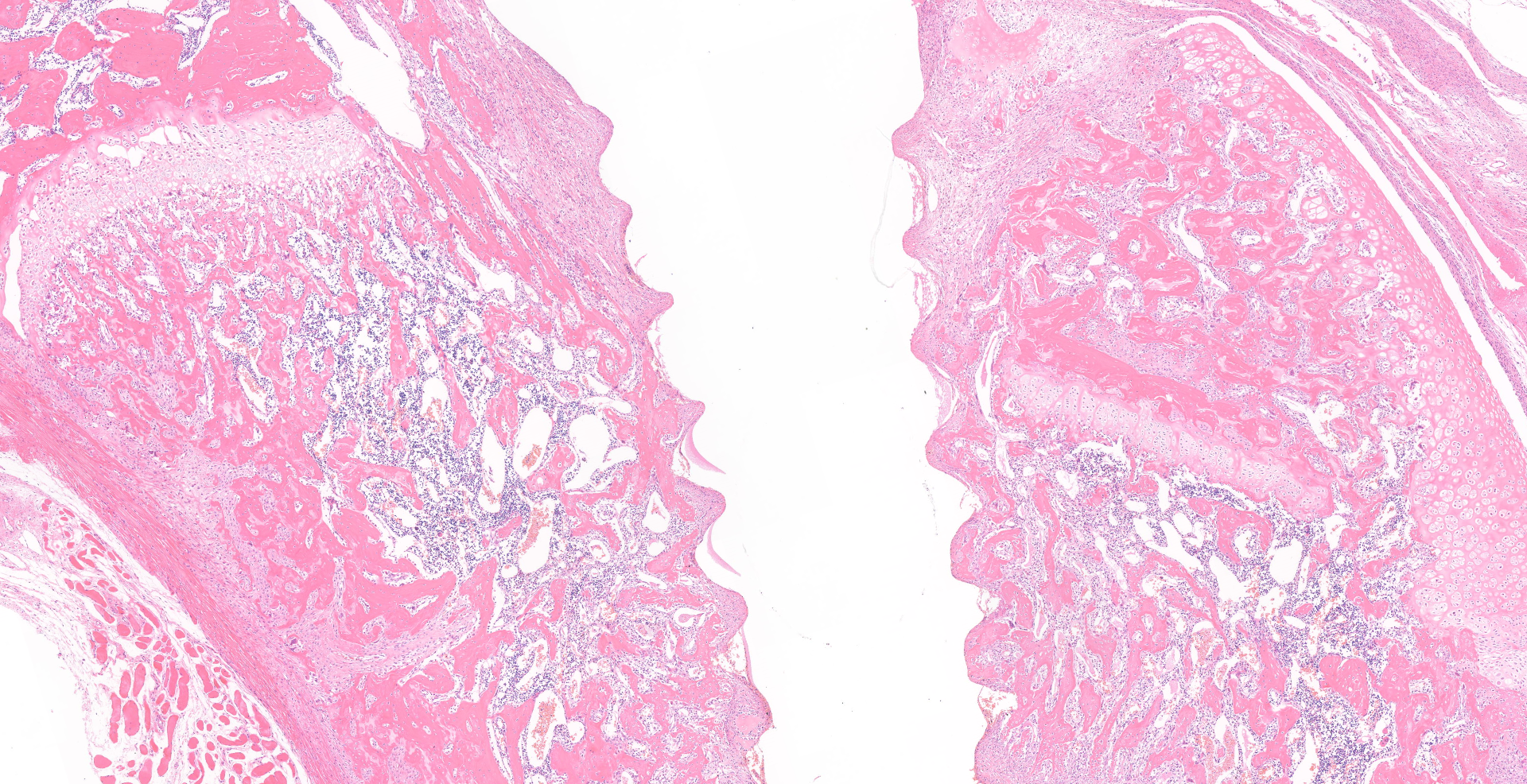

Supplement: Supplementary file 5 [file DataSheet6.ZIP › origin-HE(animal)/PJI+DEB-20X.tif]

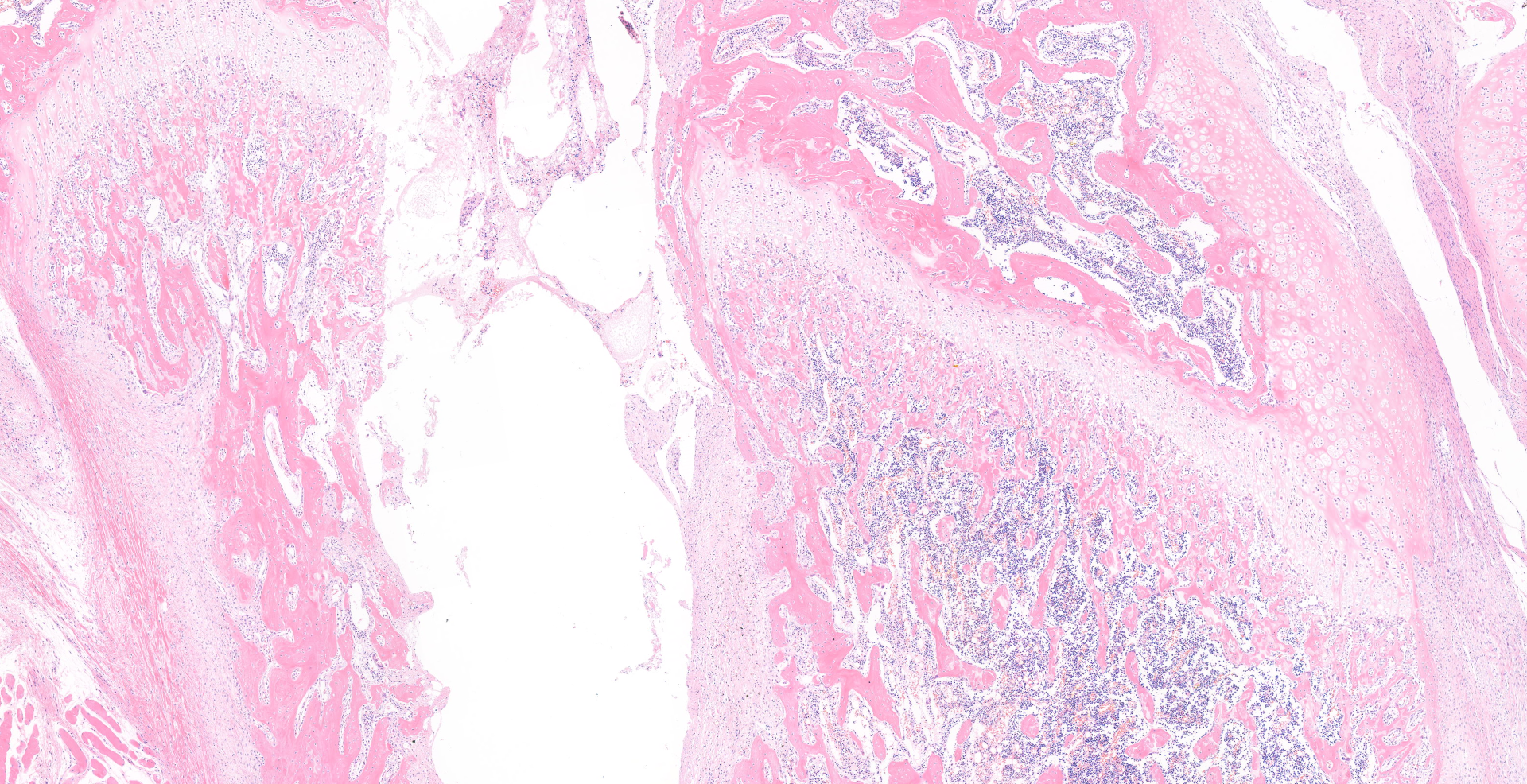

Supplement: Supplementary file 5 [file DataSheet6.ZIP › origin-HE(animal)/PJI+ECH+DEB-20X.tif]

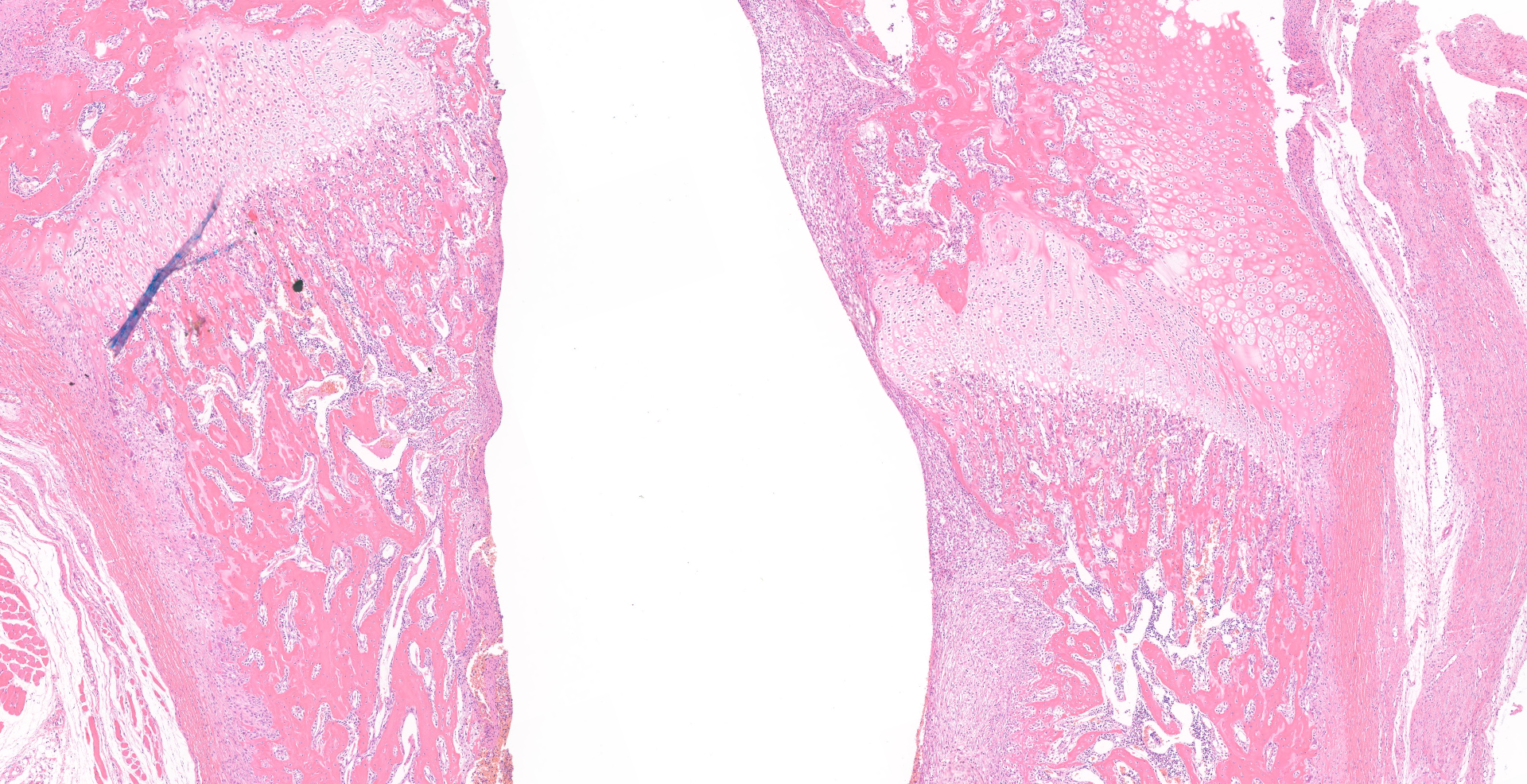

Supplement: Supplementary file 5 [file DataSheet6.ZIP › origin-HE(animal)/PJI-20X.tif]

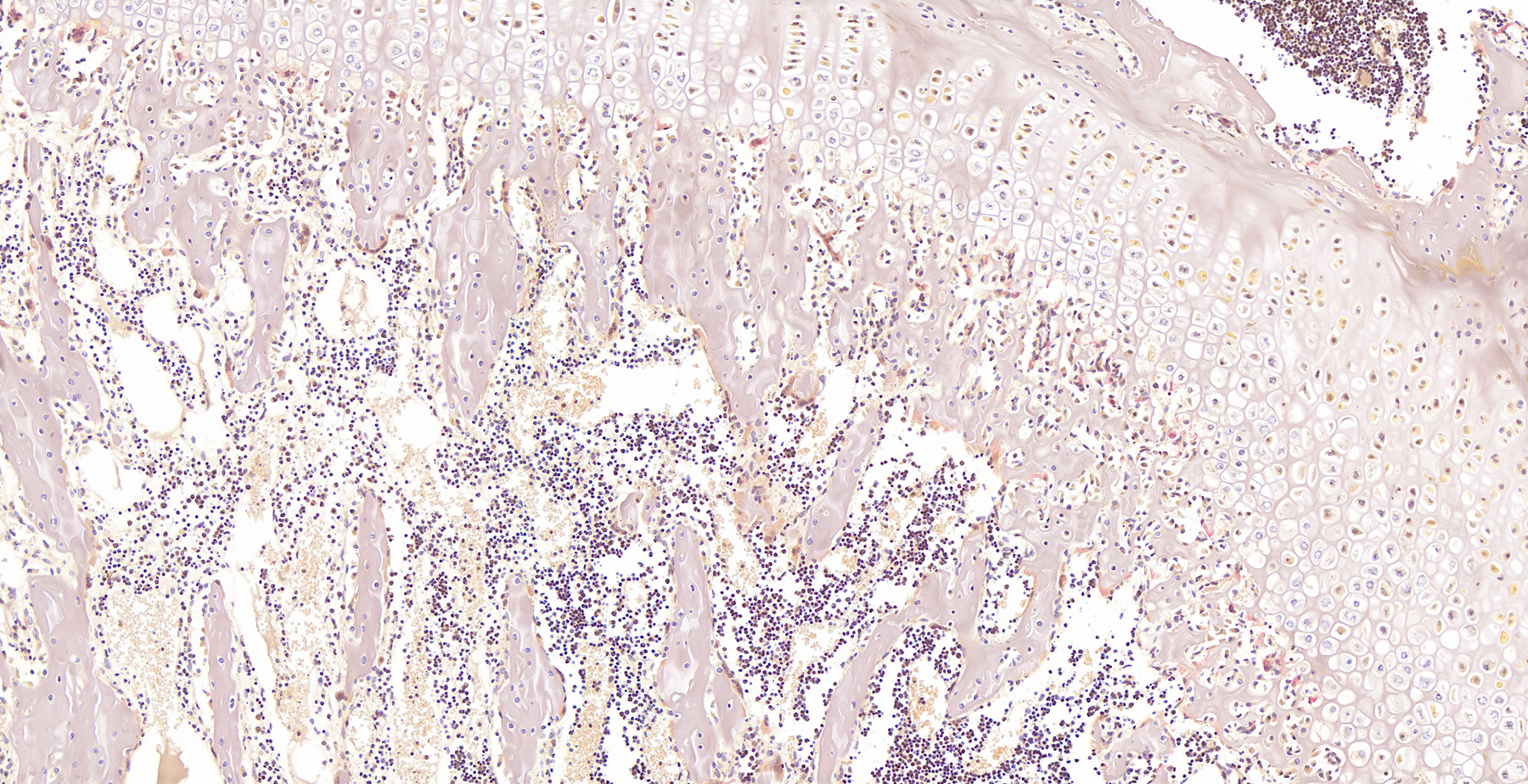

Supplement: Supplementary file 6 [file DataSheet2.ZIP › origin-TRAP(animal)/CON-200X.tif]

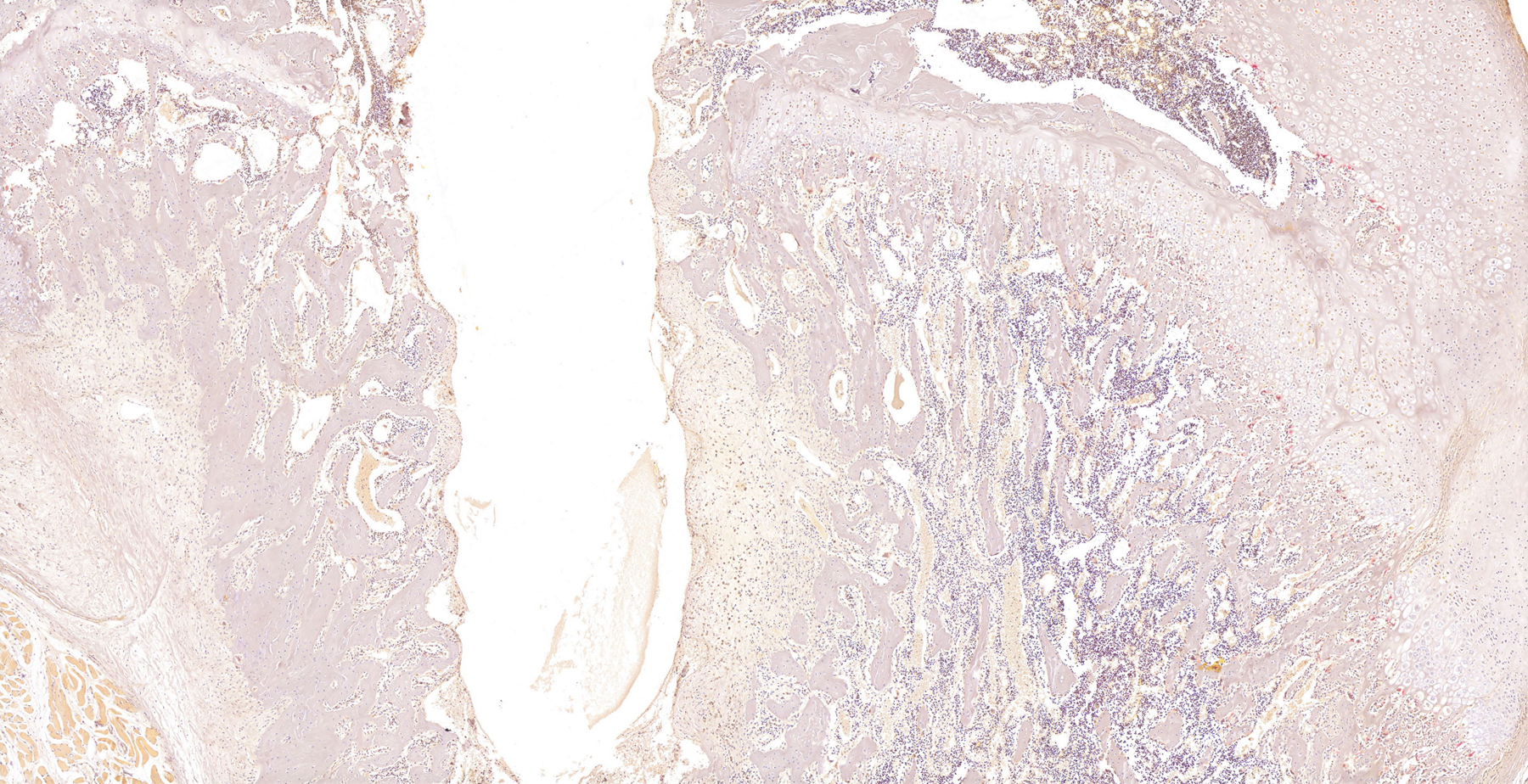

Supplement: Supplementary file 6 [file DataSheet2.ZIP › origin-TRAP(animal)/CON-20X.tif]

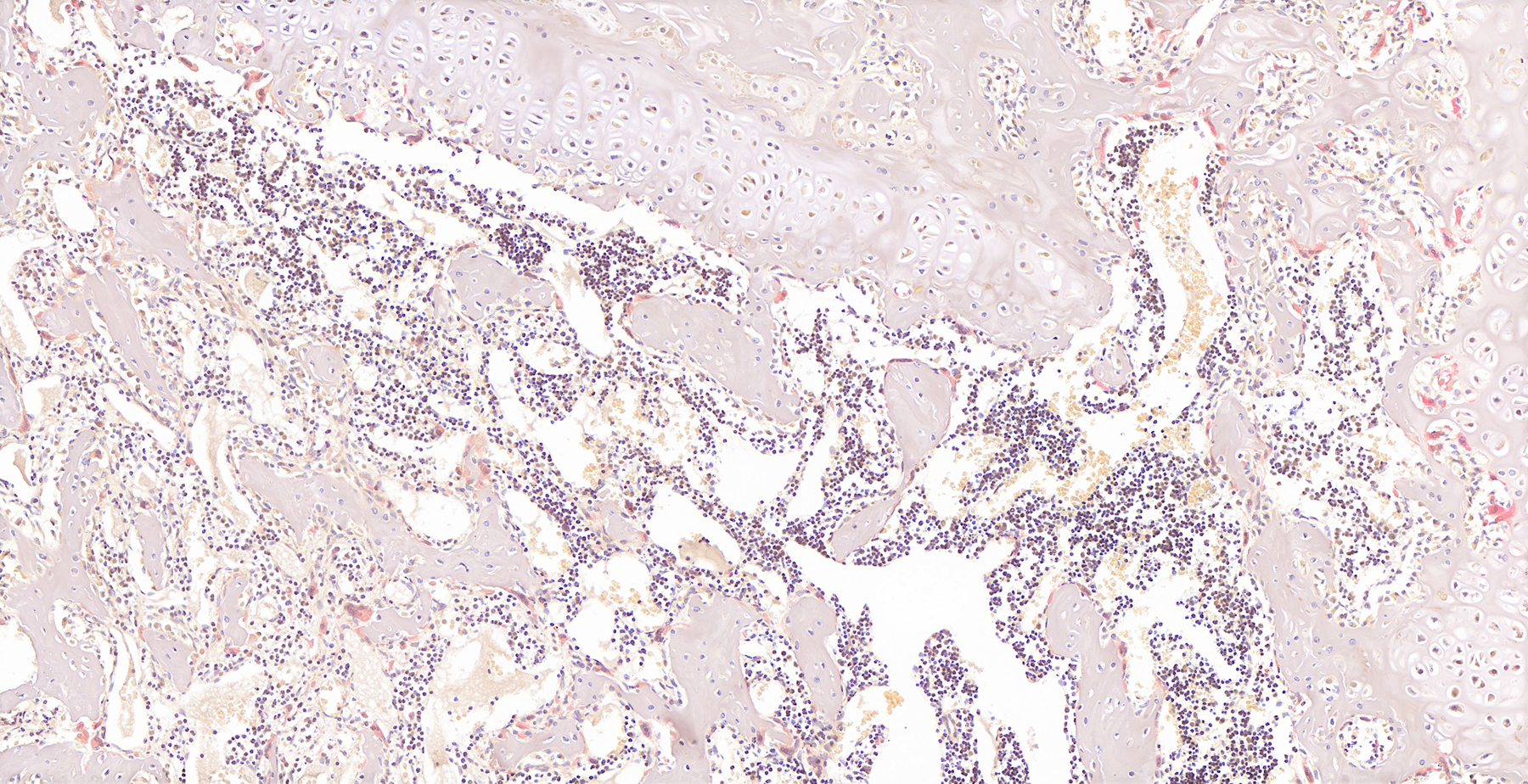

Supplement: Supplementary file 6 [file DataSheet2.ZIP › origin-TRAP(animal)/PJI+DEB-200X.tif]

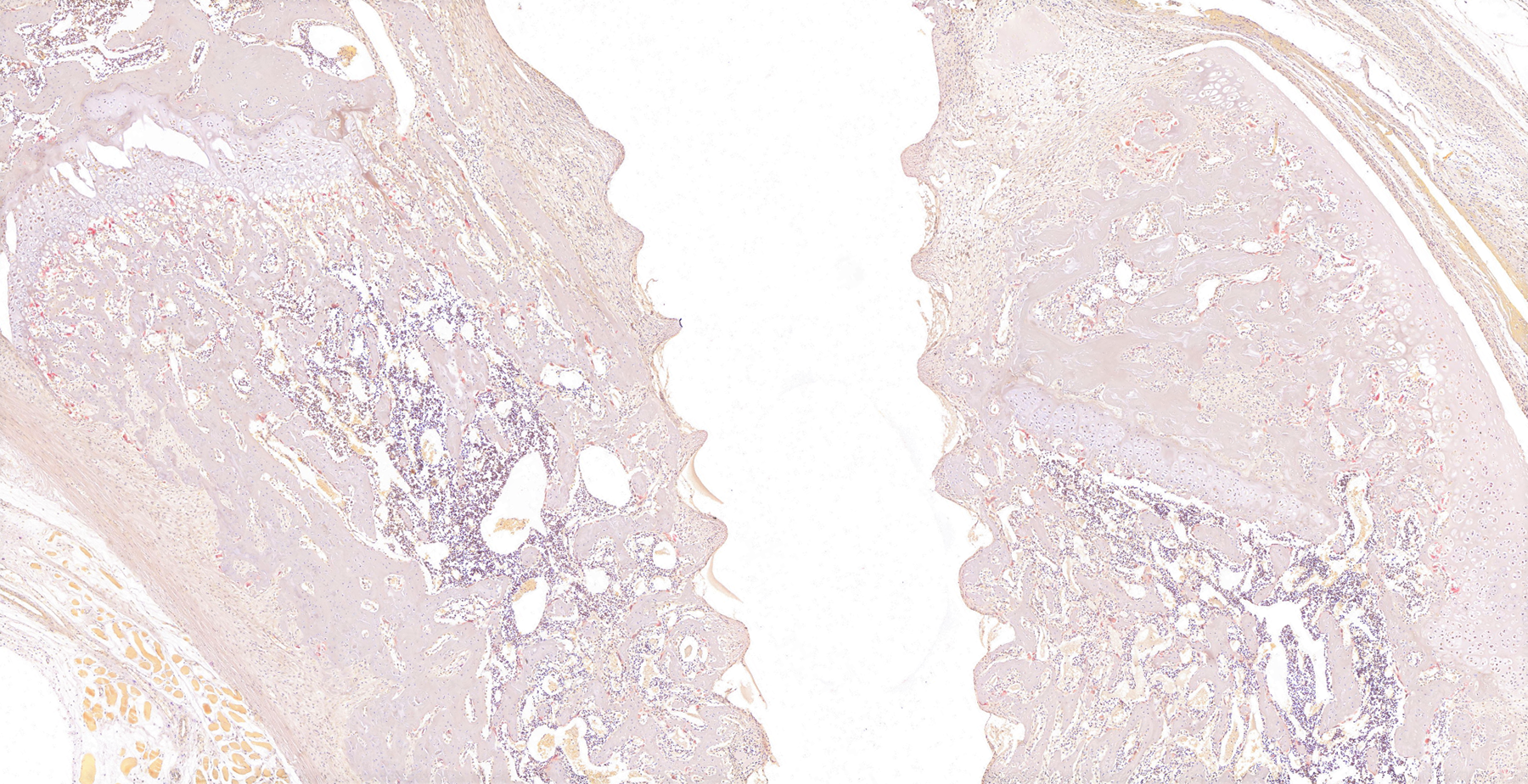

Supplement: Supplementary file 6 [file DataSheet2.ZIP › origin-TRAP(animal)/PJI+DEB-20X.tif]

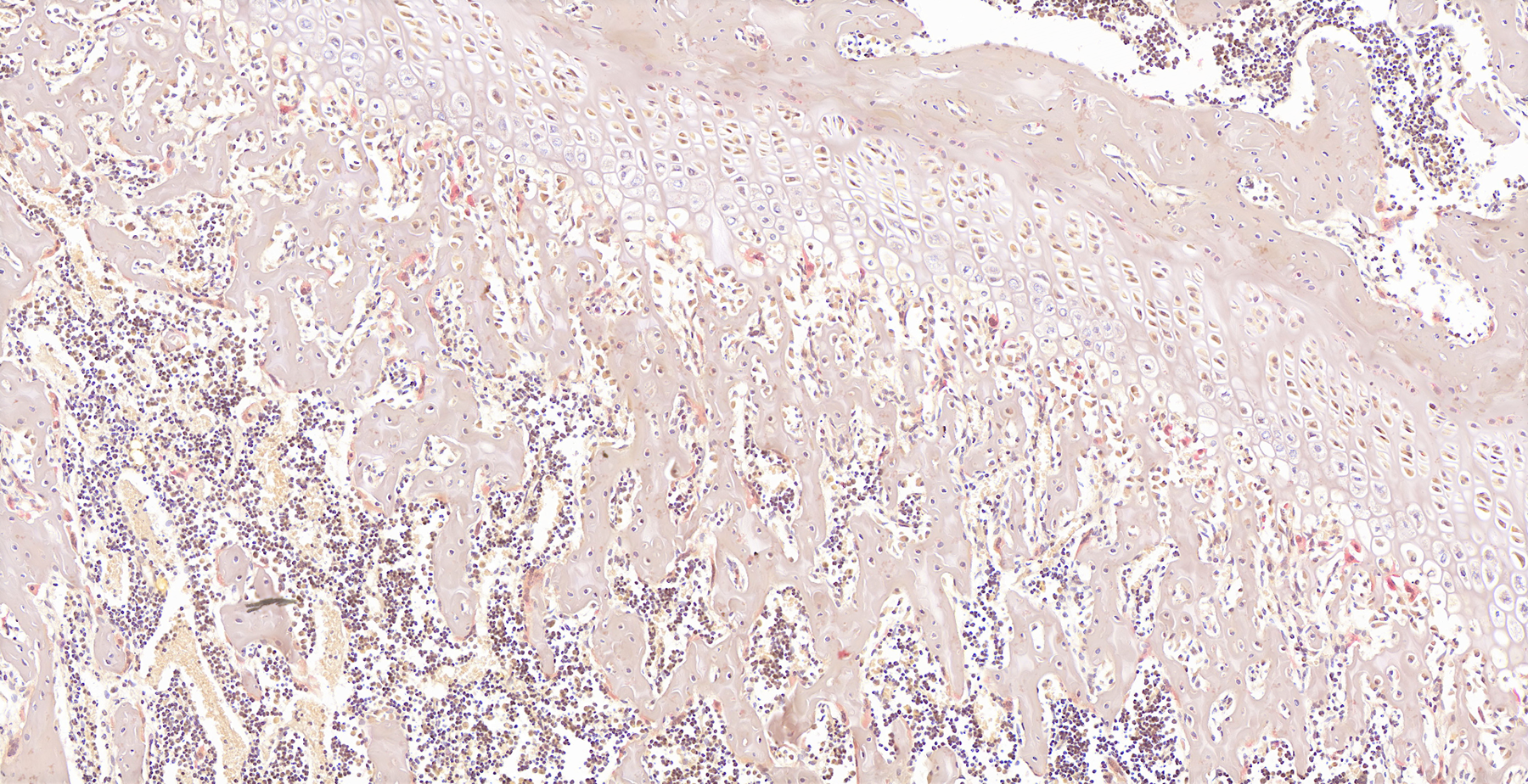

Supplement: Supplementary file 6 [file DataSheet2.ZIP › origin-TRAP(animal)/PJI+ECH+DEB-200X.tif]

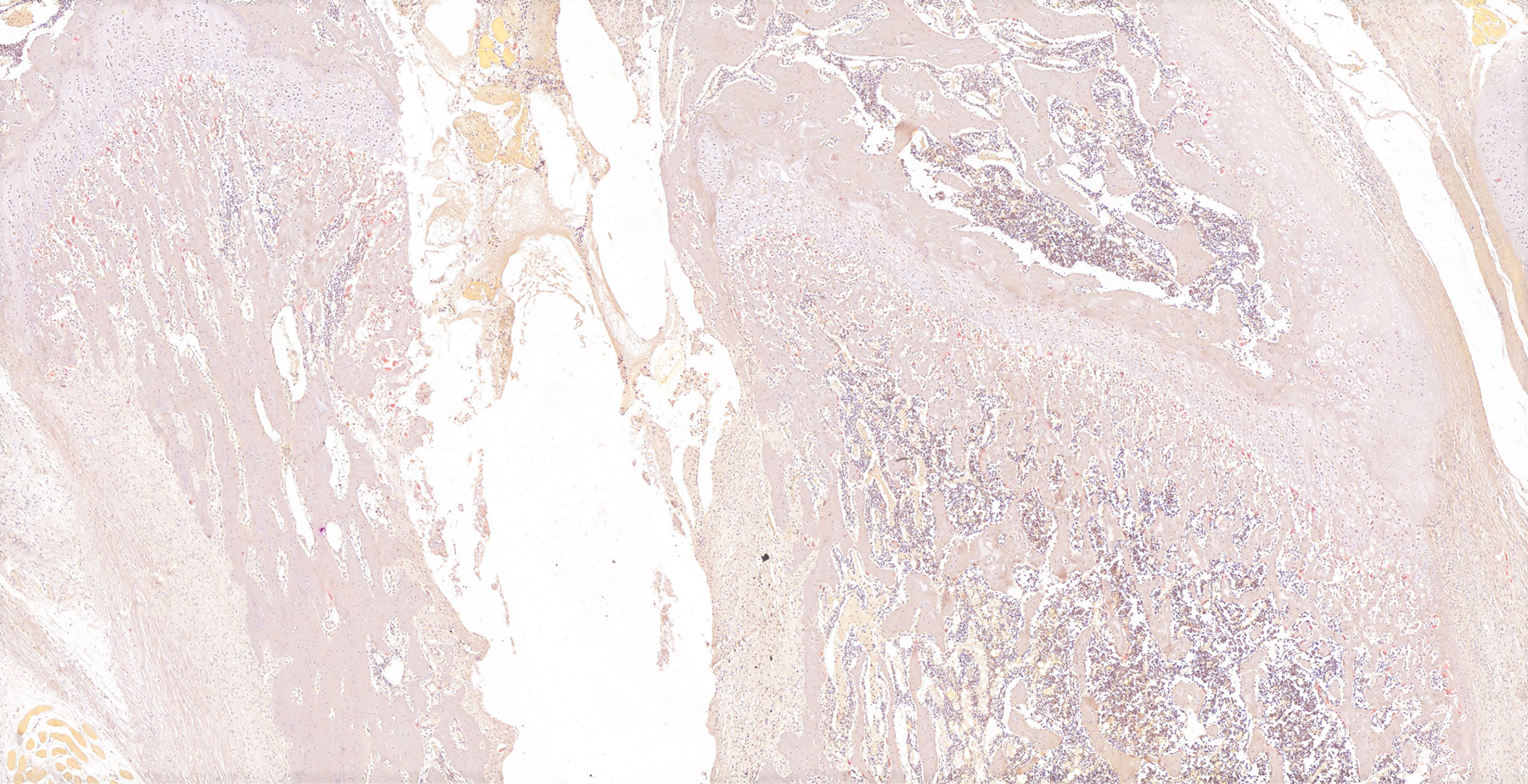

Supplement: Supplementary file 6 [file DataSheet2.ZIP › origin-TRAP(animal)/PJI+ECH+DEB-20X.tif]

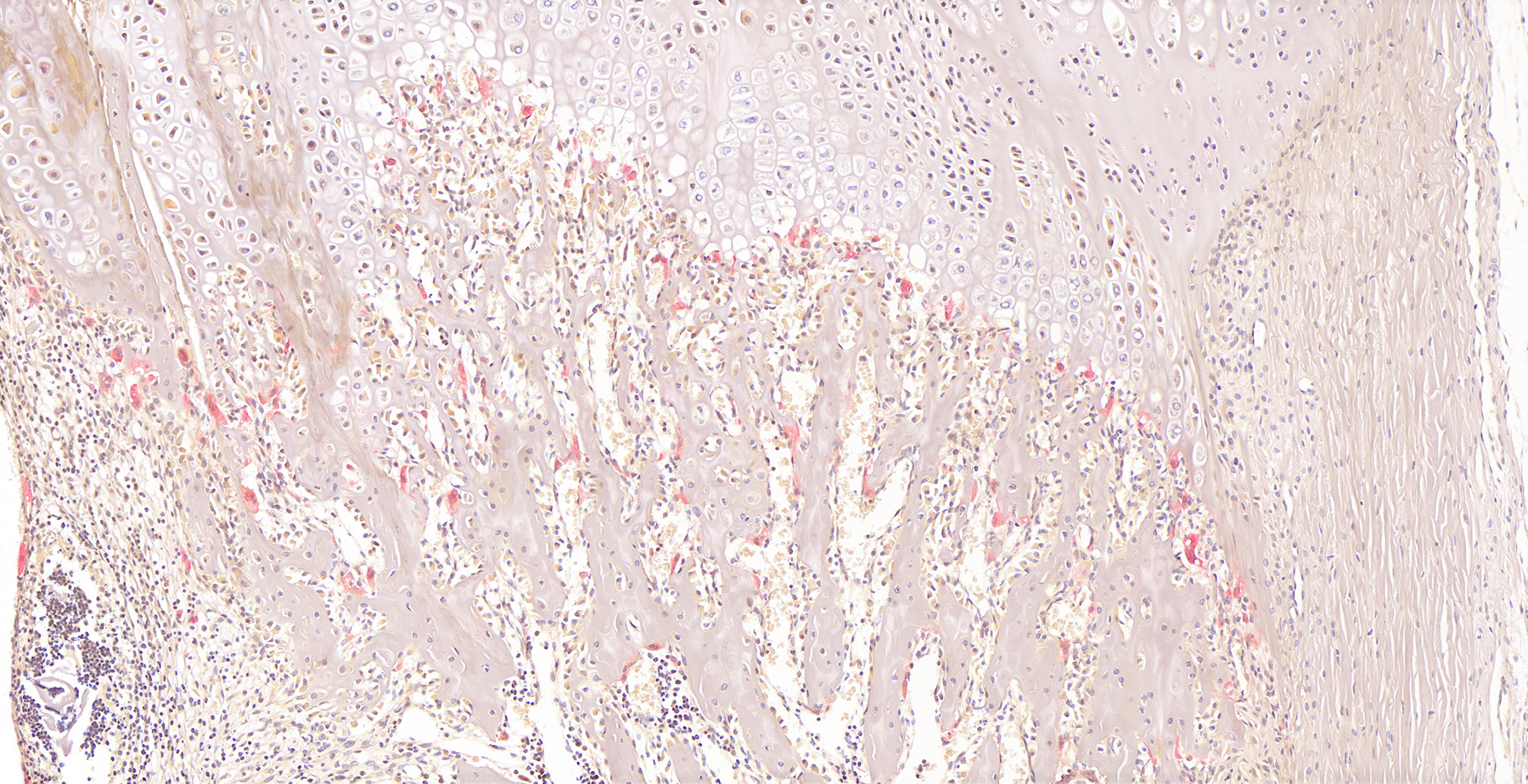

Supplement: Supplementary file 6 [file DataSheet2.ZIP › origin-TRAP(animal)/PJI-200X.tif]

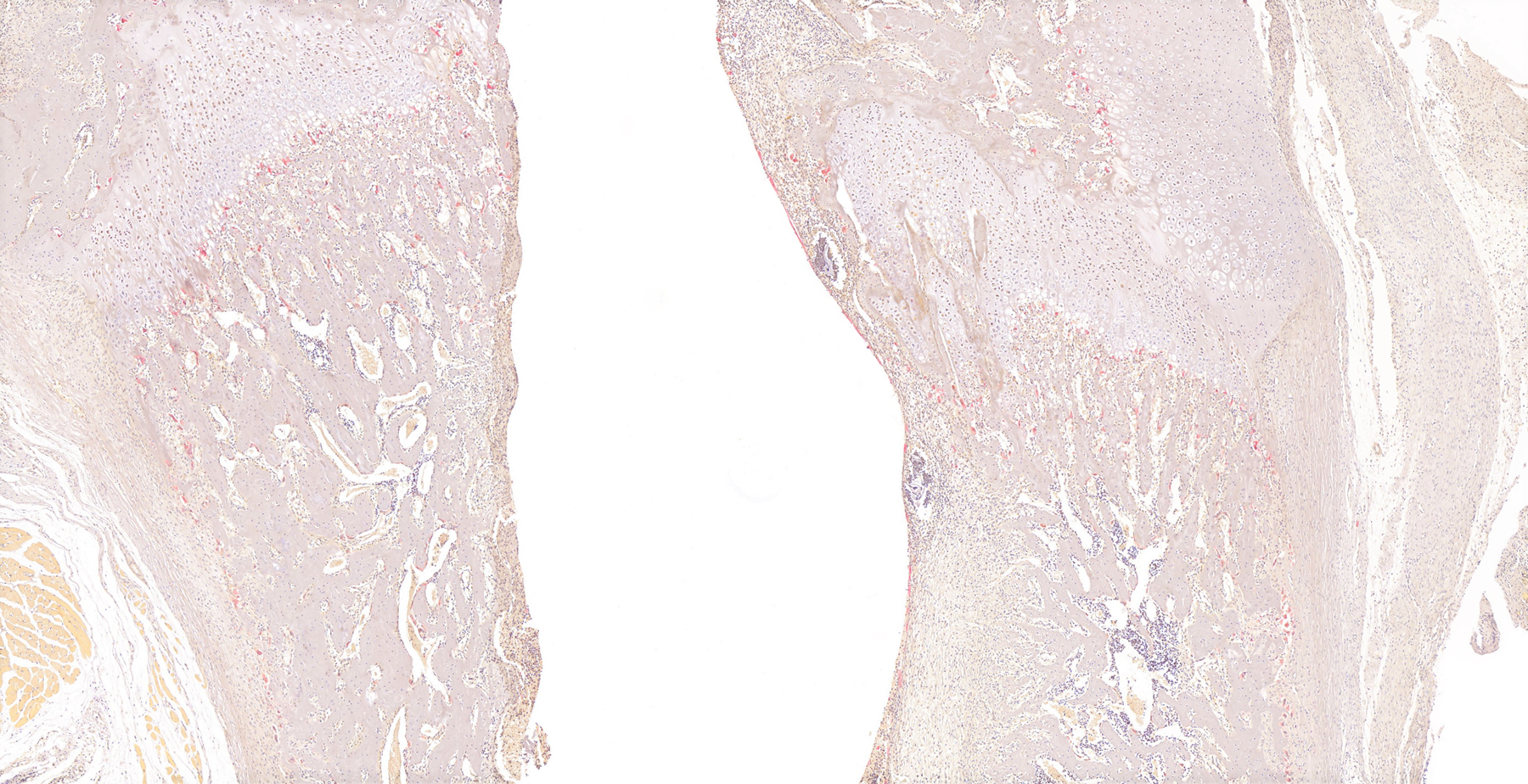

Supplement: Supplementary file 6 [file DataSheet2.ZIP › origin-TRAP(animal)/PJI-20X.tif]

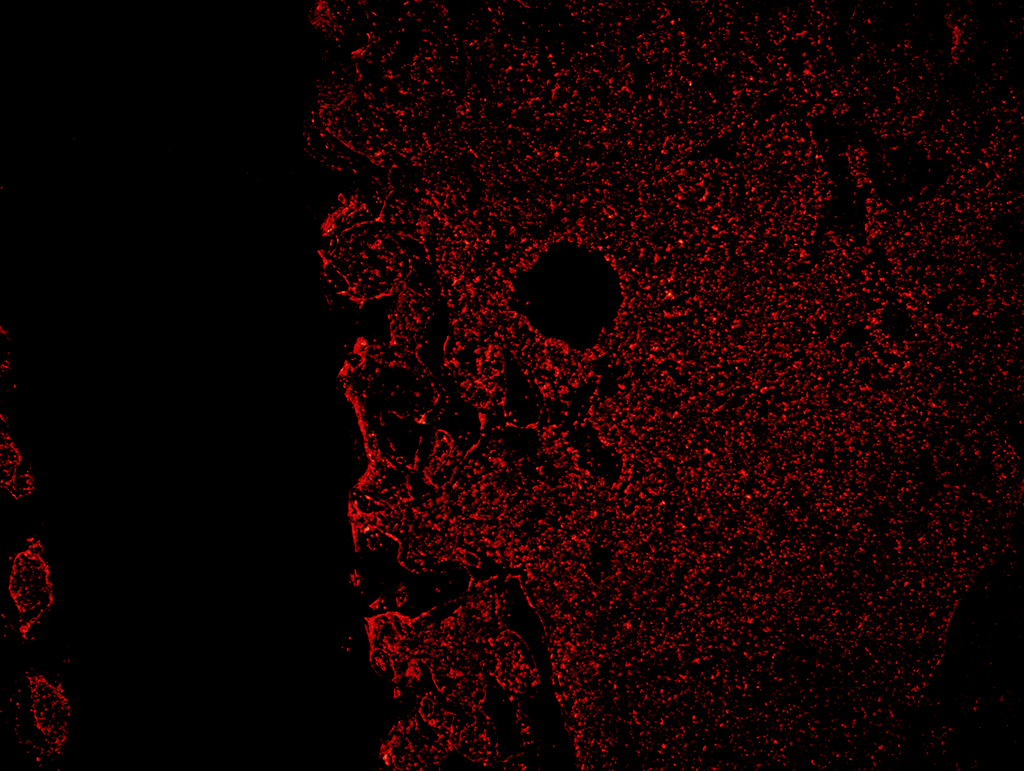

Supplement: Supplementary file 8 [file DataSheet5.ZIP › origin-IF(animal)/CON-c-Fos-40X.tif]

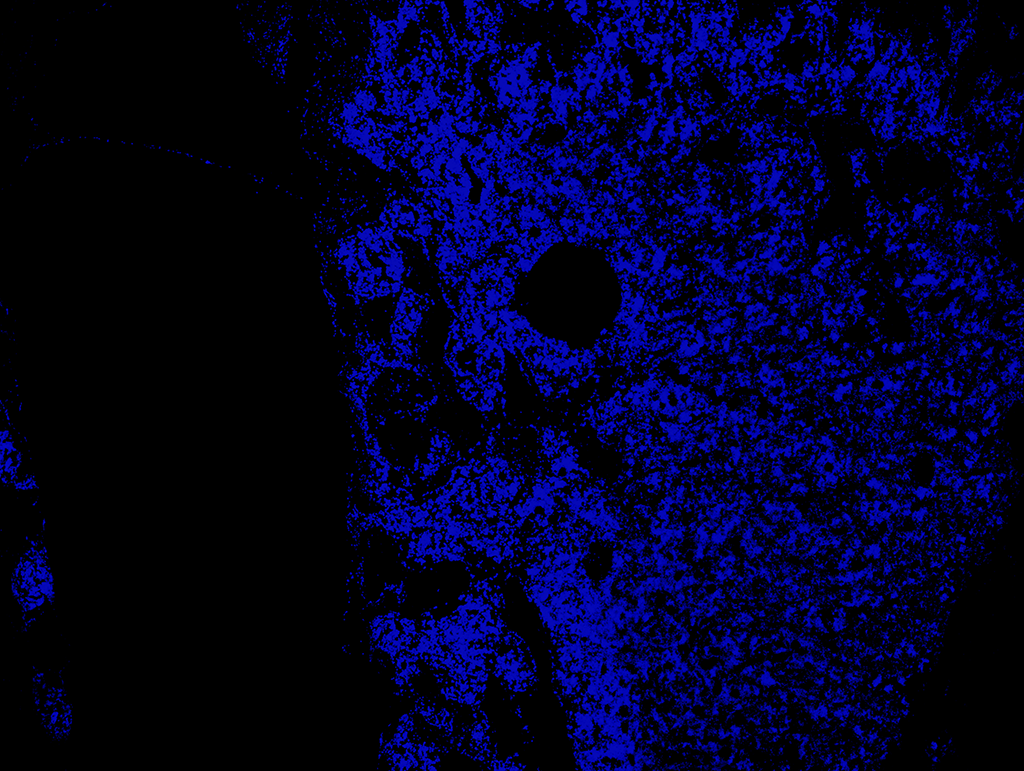

Supplement: Supplementary file 8 [file DataSheet5.ZIP › origin-IF(animal)/CON-DAPI-40X.tif]

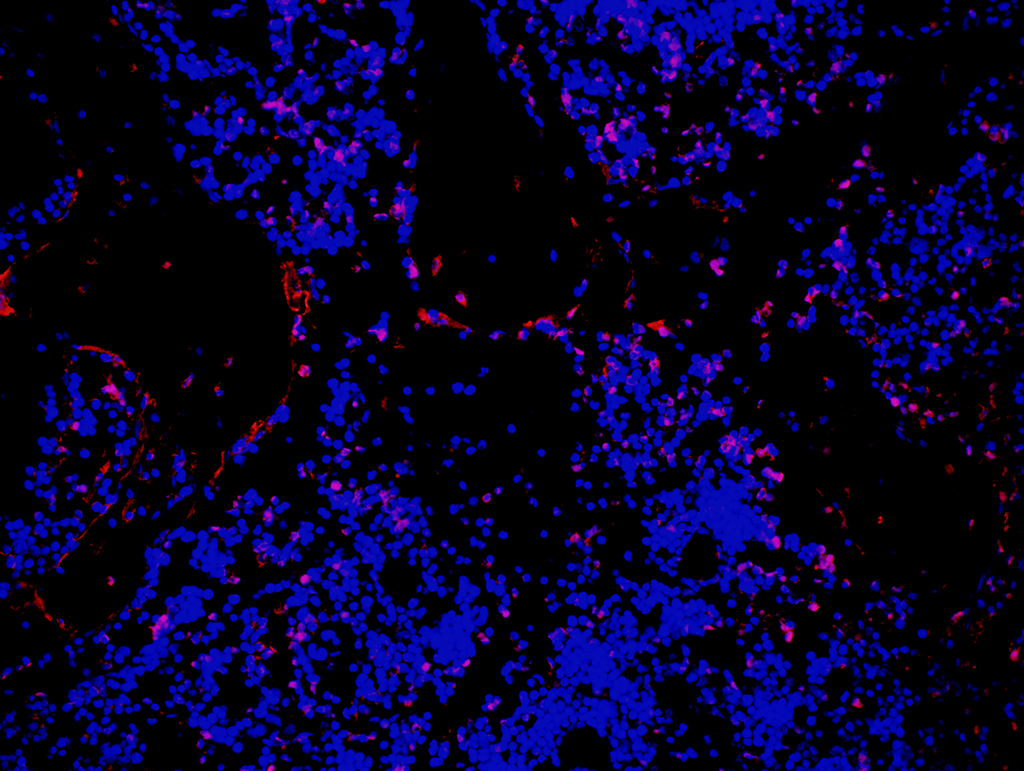

Supplement: Supplementary file 8 [file DataSheet5.ZIP › origin-IF(animal)/CON-merge-200X.tif]

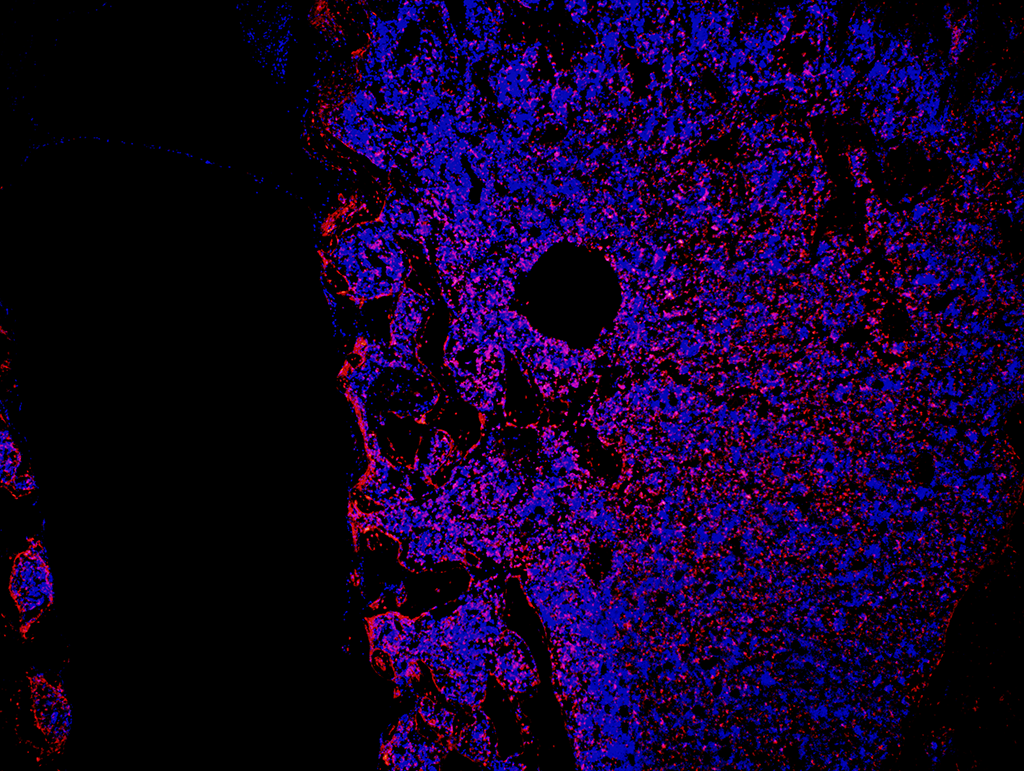

Supplement: Supplementary file 8 [file DataSheet5.ZIP › origin-IF(animal)/CON-merge-40X.tif]

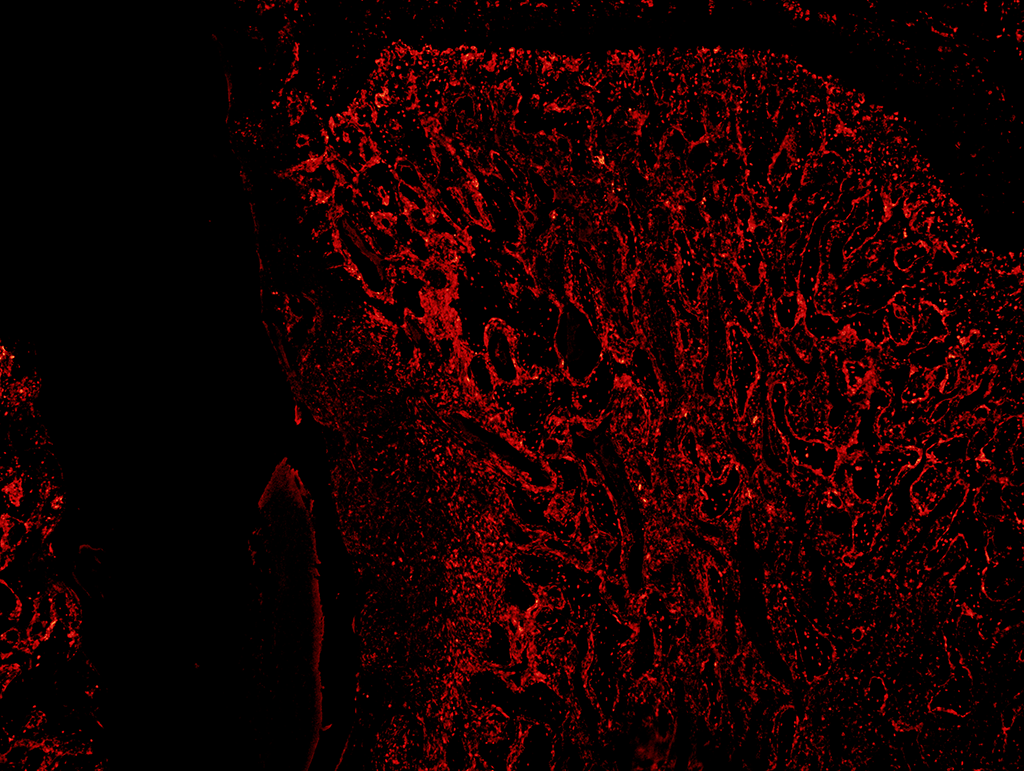

Supplement: Supplementary file 8 [file DataSheet5.ZIP › origin-IF(animal)/PJI+DEB-c-Fos-40X.tif]

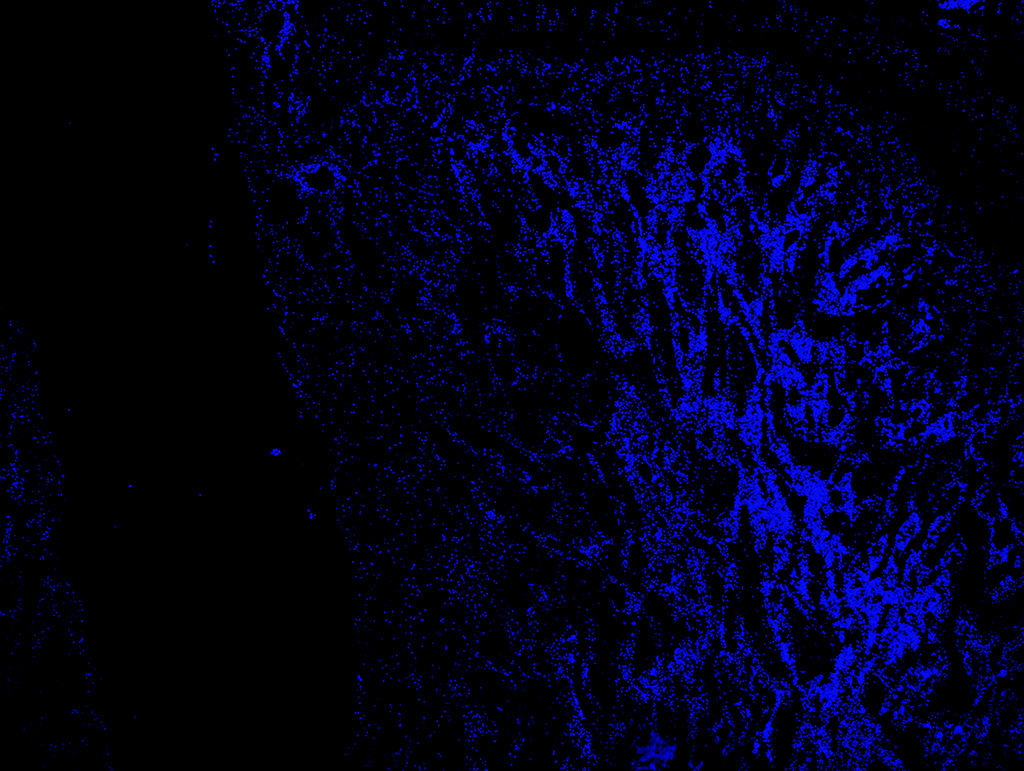

Supplement: Supplementary file 8 [file DataSheet5.ZIP › origin-IF(animal)/PJI+DEB-DAPI-40X.tif]

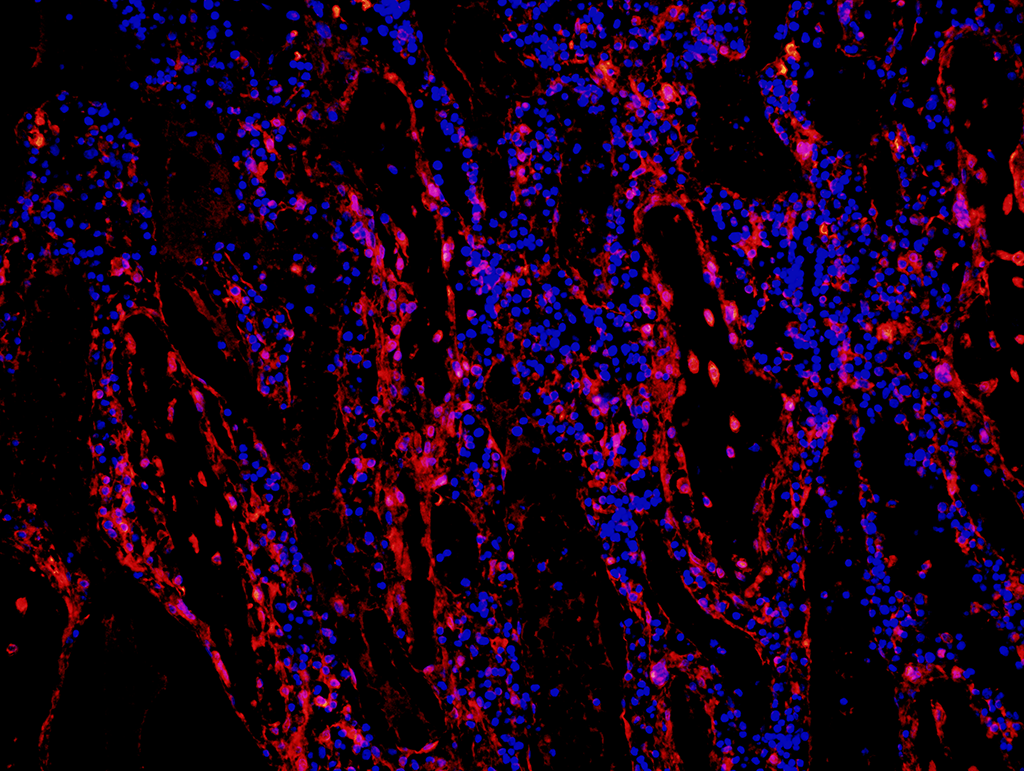

Supplement: Supplementary file 8 [file DataSheet5.ZIP › origin-IF(animal)/PJI+DEB-merge-200X.tif]

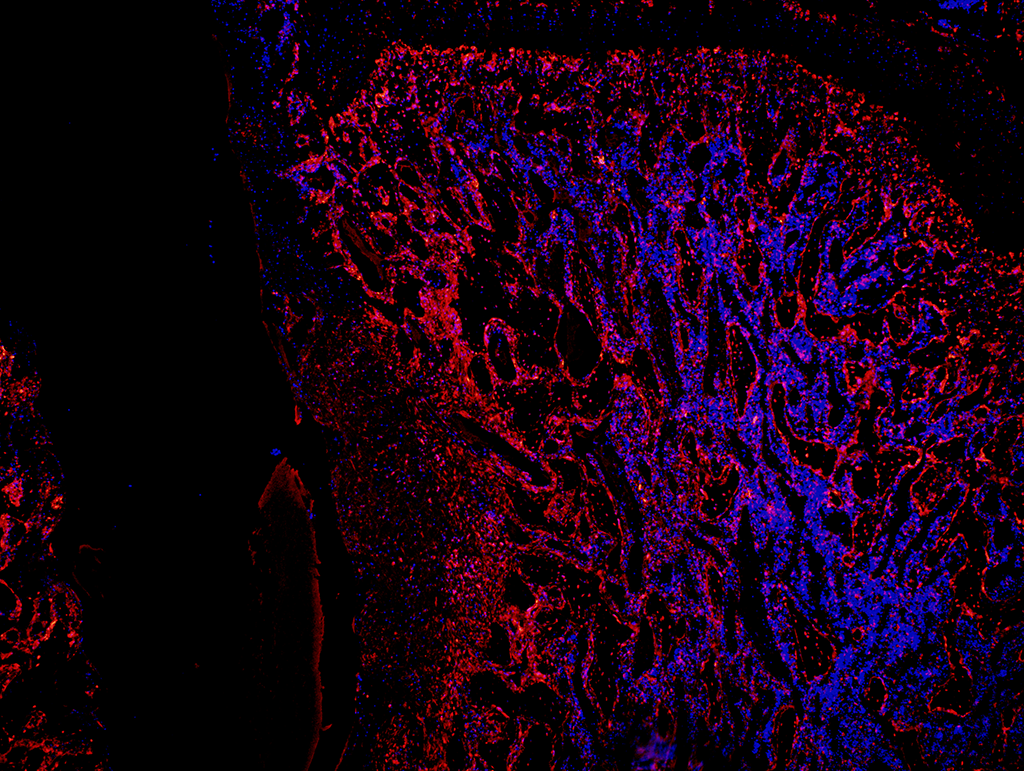

Supplement: Supplementary file 8 [file DataSheet5.ZIP › origin-IF(animal)/PJI+DEB-merge-40X.tif]

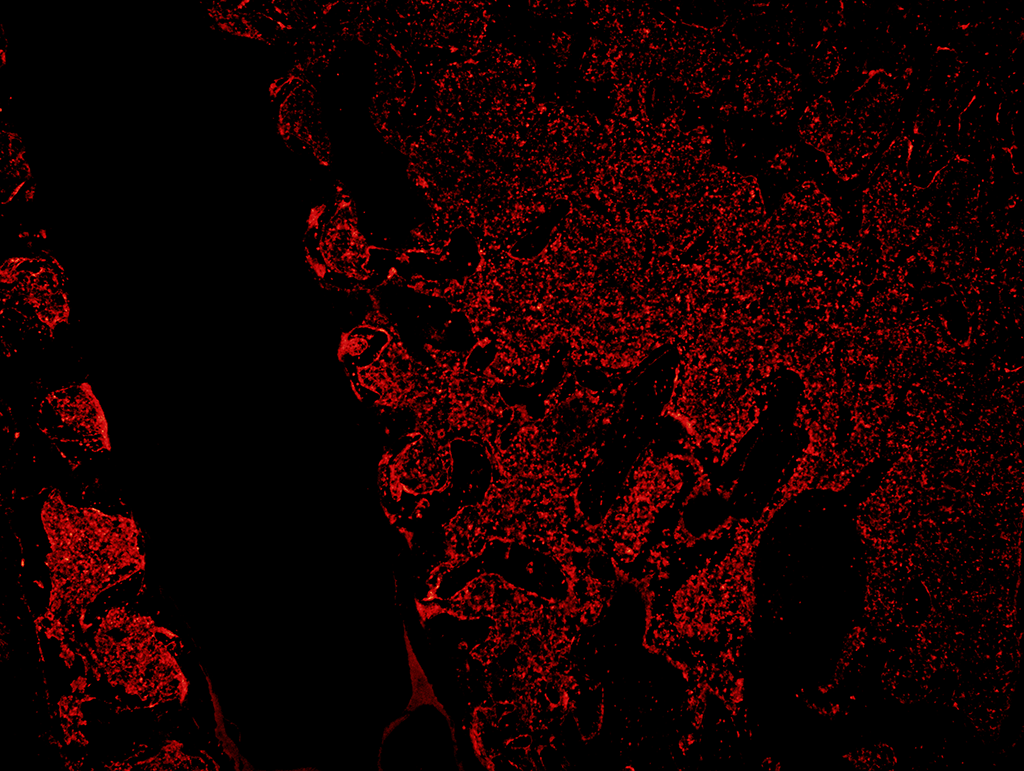

Supplement: Supplementary file 8 [file DataSheet5.ZIP › origin-IF(animal)/PJI+ECH+DEB-c-Fos-40X.tif]

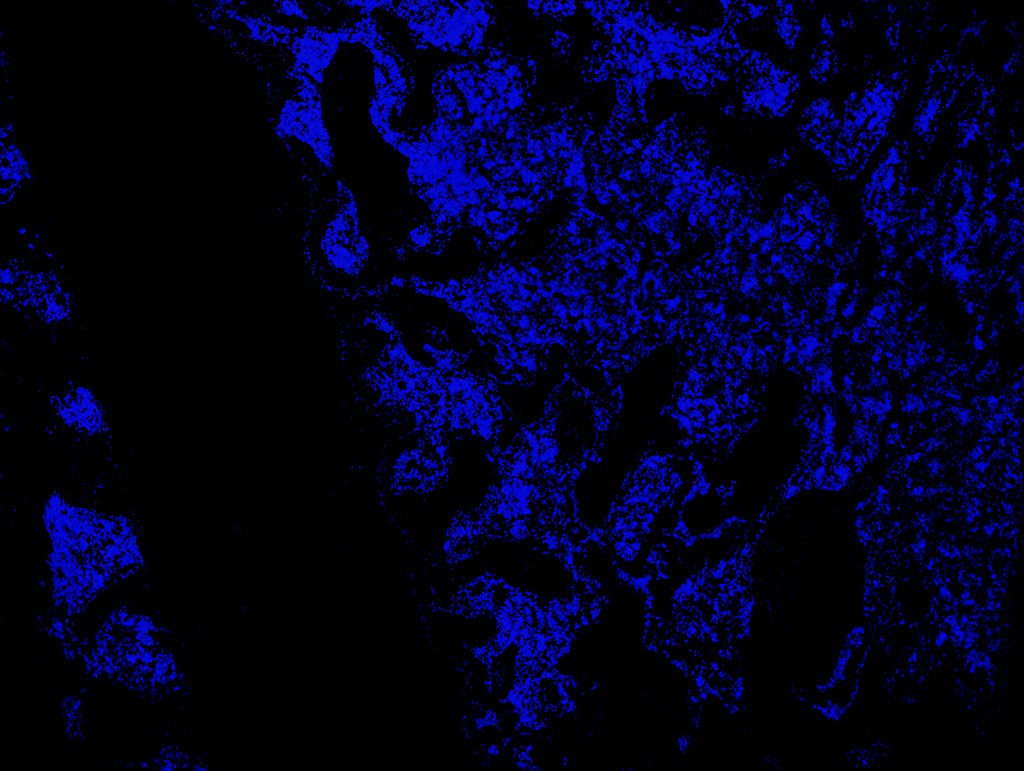

Supplement: Supplementary file 8 [file DataSheet5.ZIP › origin-IF(animal)/PJI+ECH+DEB-DAPI-40X.tif]

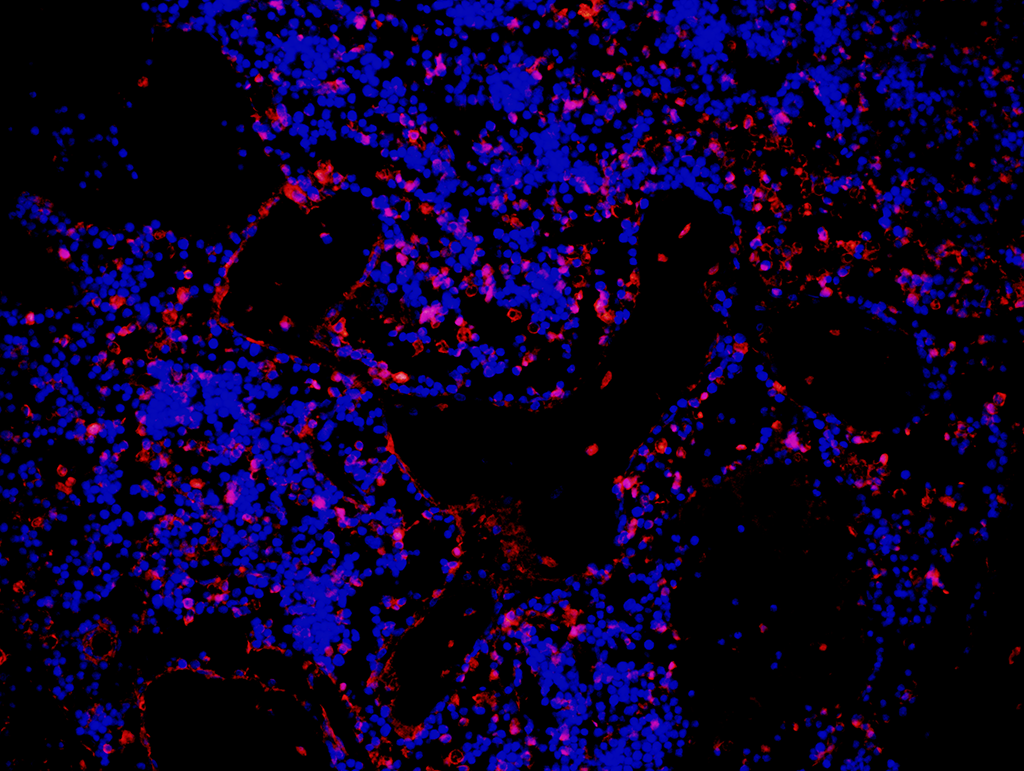

Supplement: Supplementary file 8 [file DataSheet5.ZIP › origin-IF(animal)/PJI+ECH+DEB-merge-200X.tif]

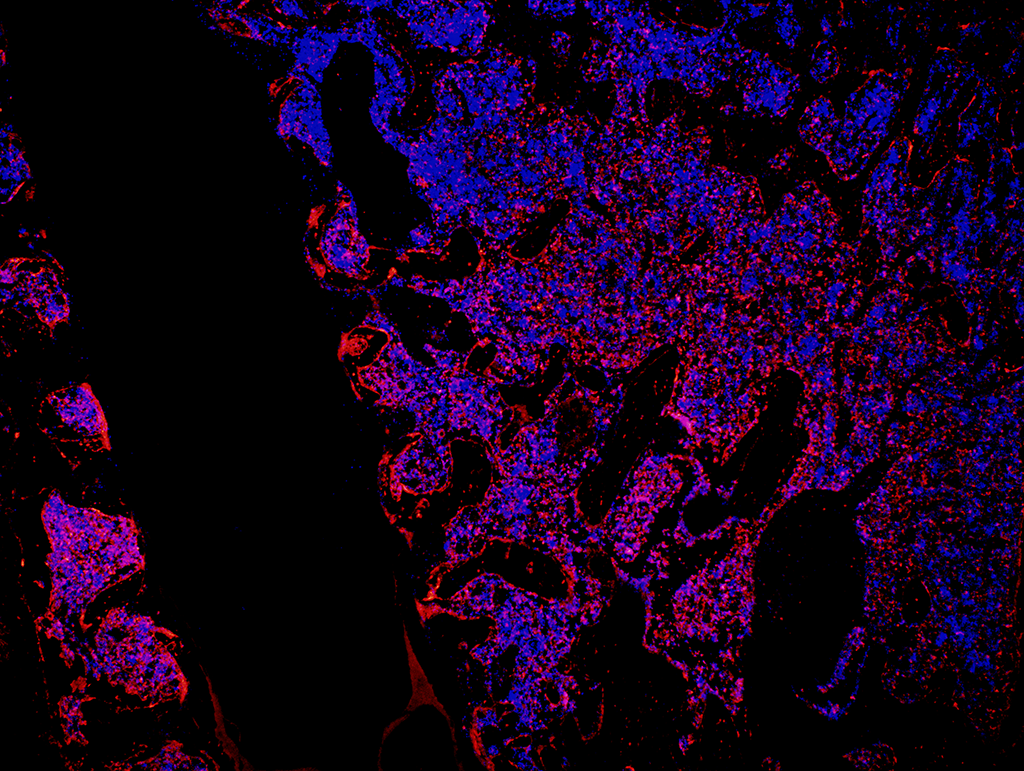

Supplement: Supplementary file 8 [file DataSheet5.ZIP › origin-IF(animal)/PJI+ECH+DEB-merge-40X.tif]

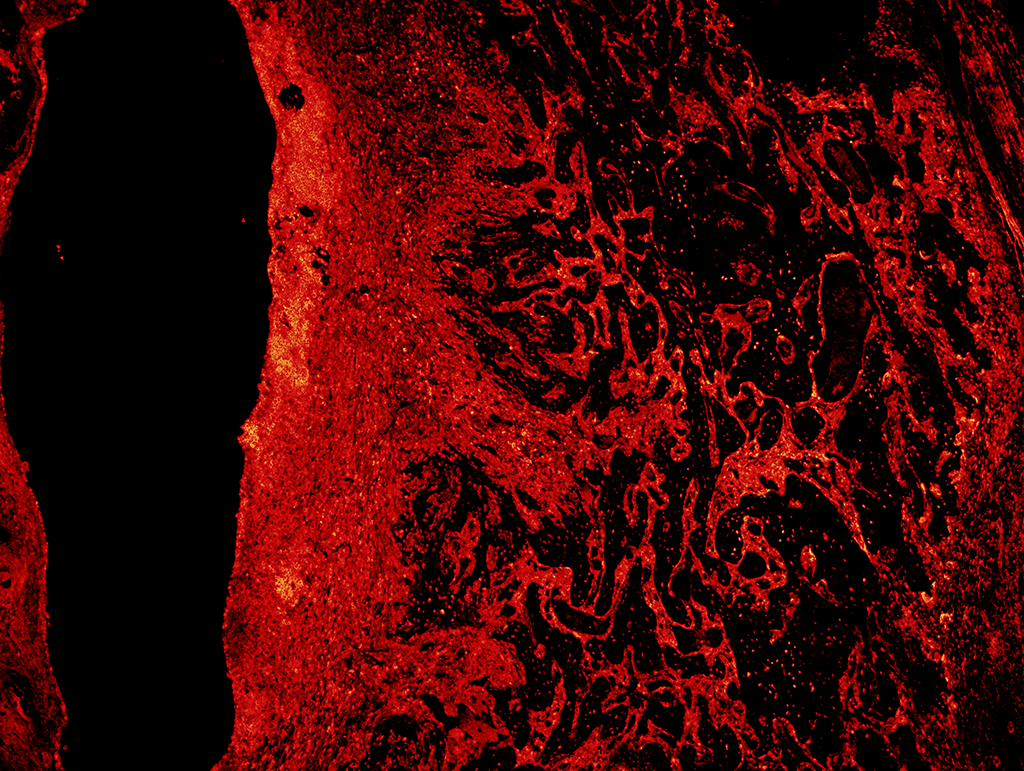

Supplement: Supplementary file 8 [file DataSheet5.ZIP › origin-IF(animal)/PJI-c-Fos-40X.tif]

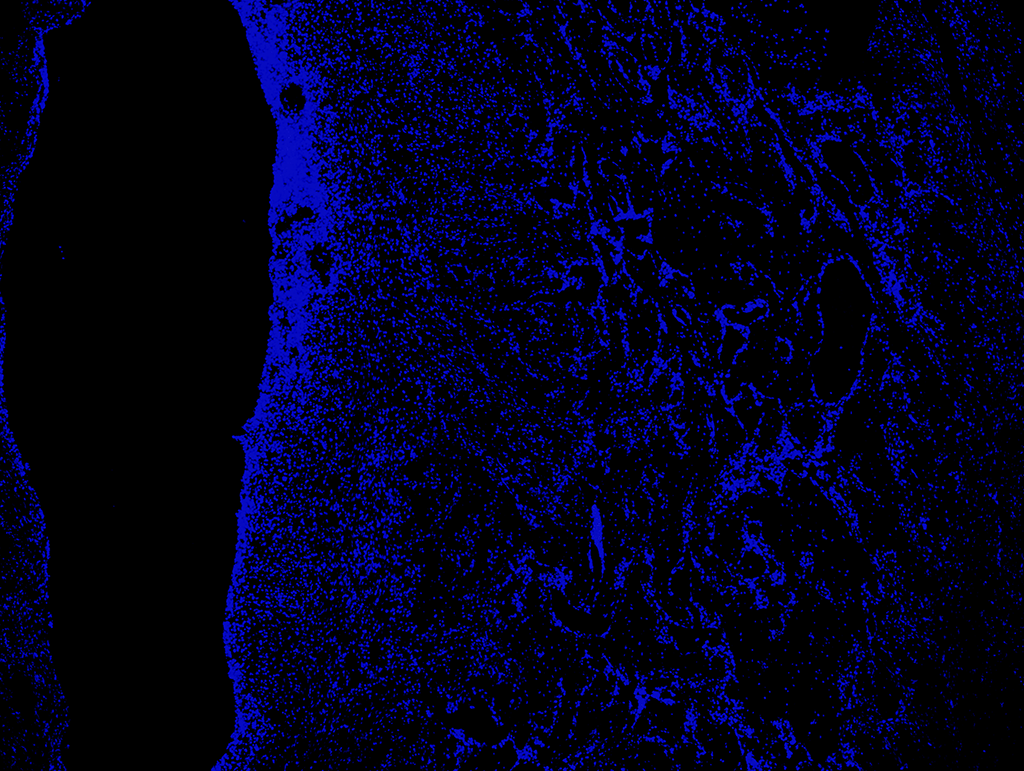

Supplement: Supplementary file 8 [file DataSheet5.ZIP › origin-IF(animal)/PJI-DAPI-40X.tif]

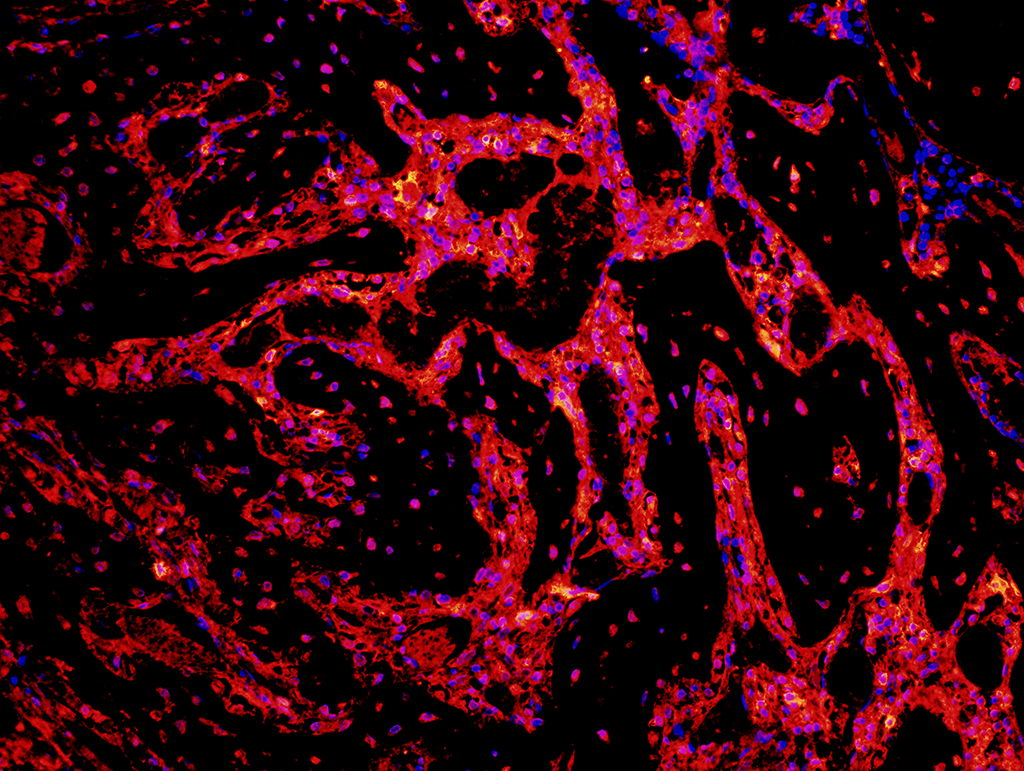

Supplement: Supplementary file 8 [file DataSheet5.ZIP › origin-IF(animal)/PJI-merge-200X.tif]

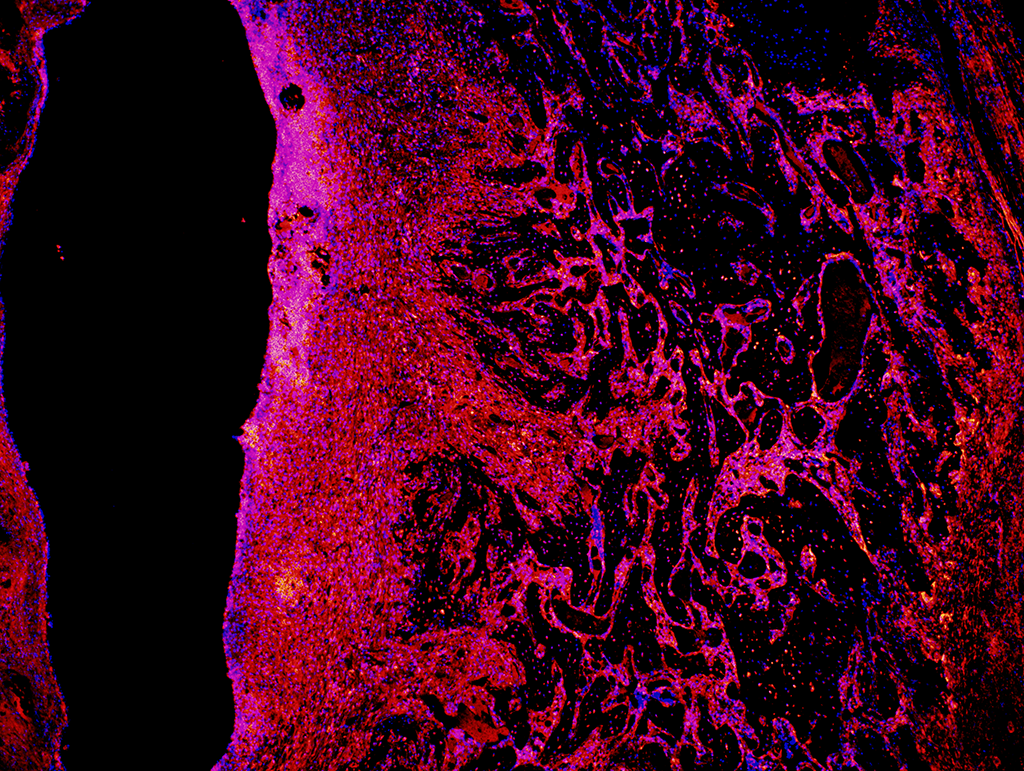

Supplement: Supplementary file 8 [file DataSheet5.ZIP › origin-IF(animal)/PJI-merge-40X.tif]

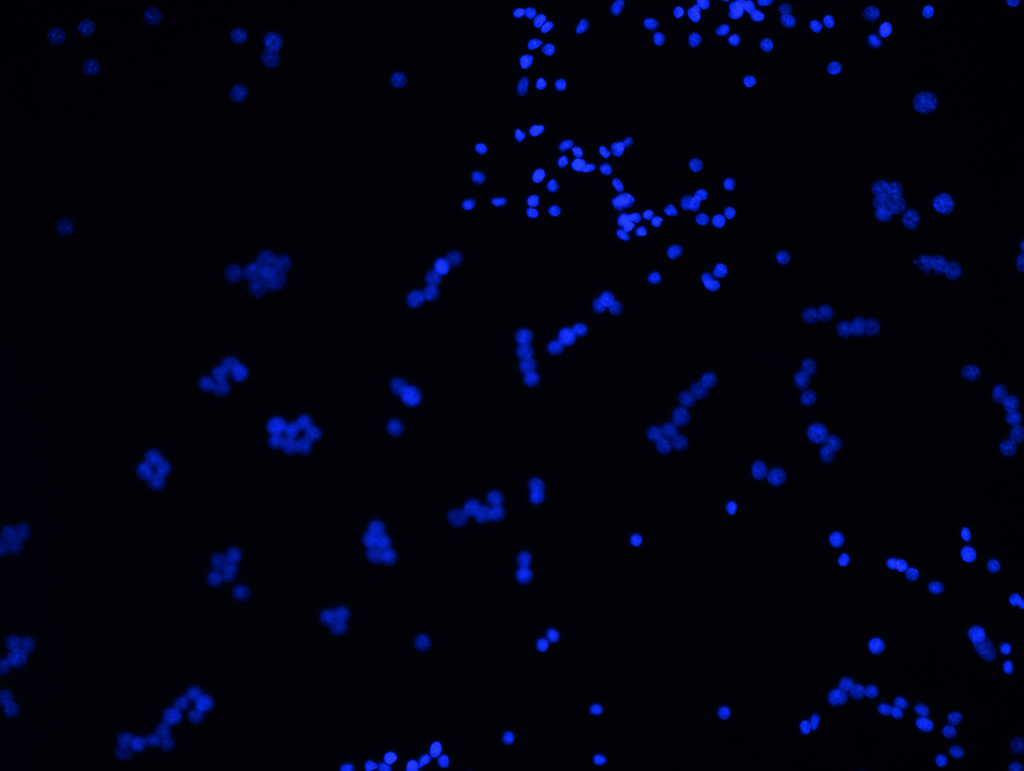

Supplement: Supplementary file 9 [file DataSheet7.ZIP › origin-F-actin(cell)/ECH-0 μM-DAPI.tif]

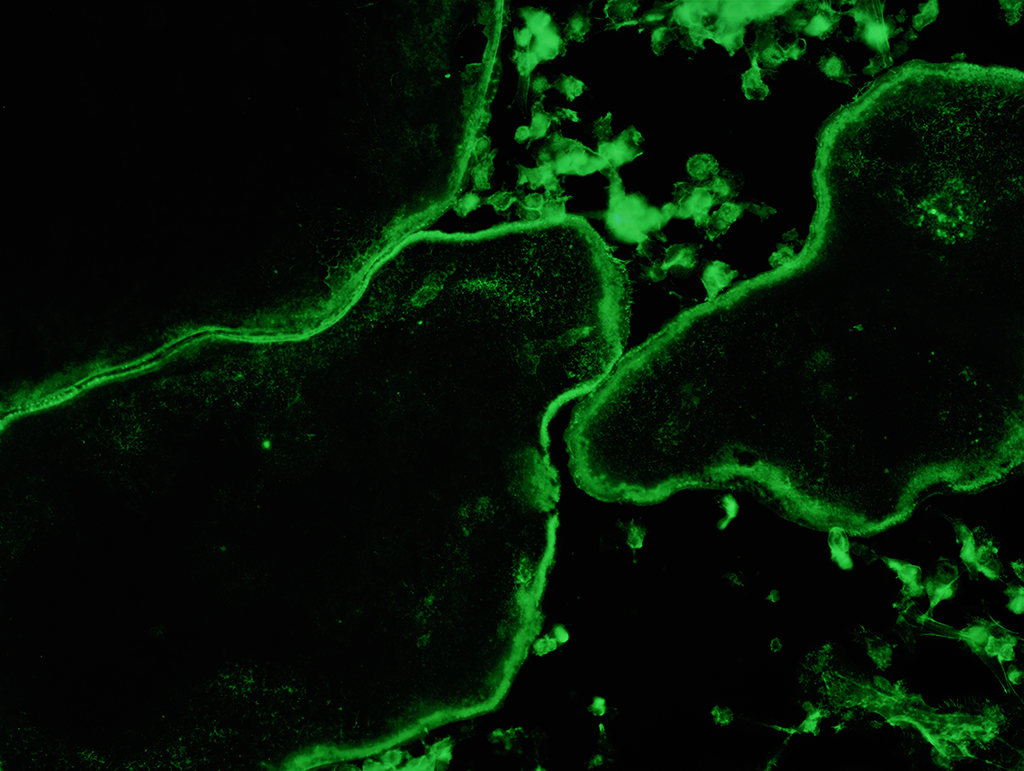

Supplement: Supplementary file 9 [file DataSheet7.ZIP › origin-F-actin(cell)/ECH-0 μM-F-actin.tif]

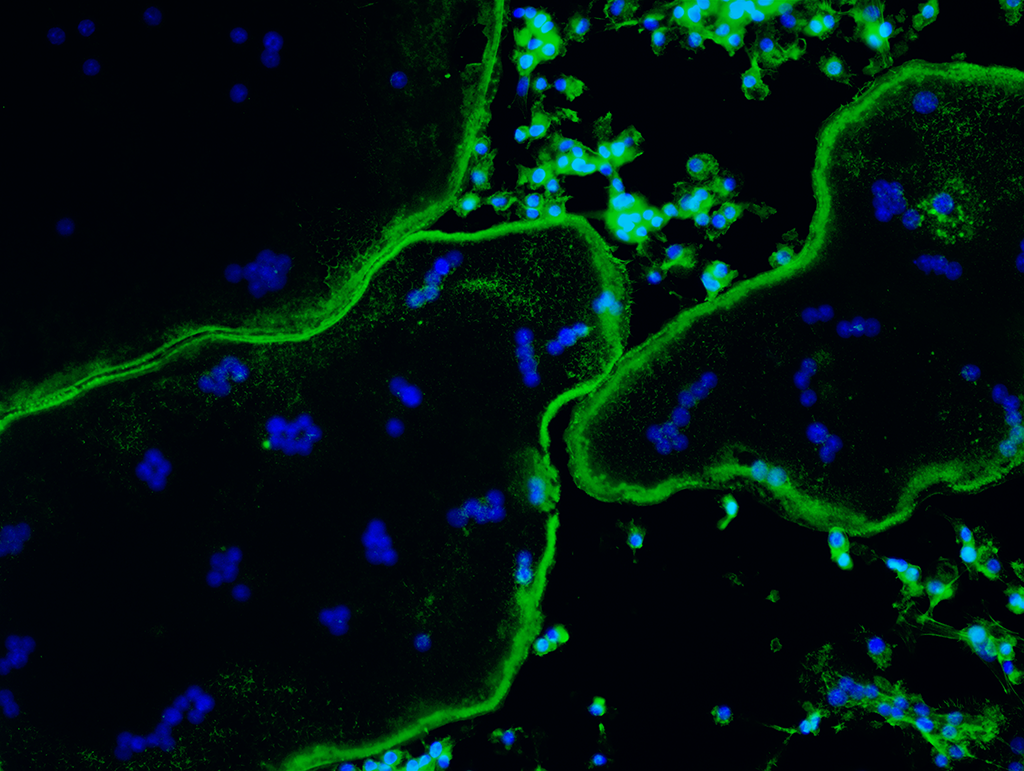

Supplement: Supplementary file 9 [file DataSheet7.ZIP › origin-F-actin(cell)/ECH-0 μM-merge.tif]

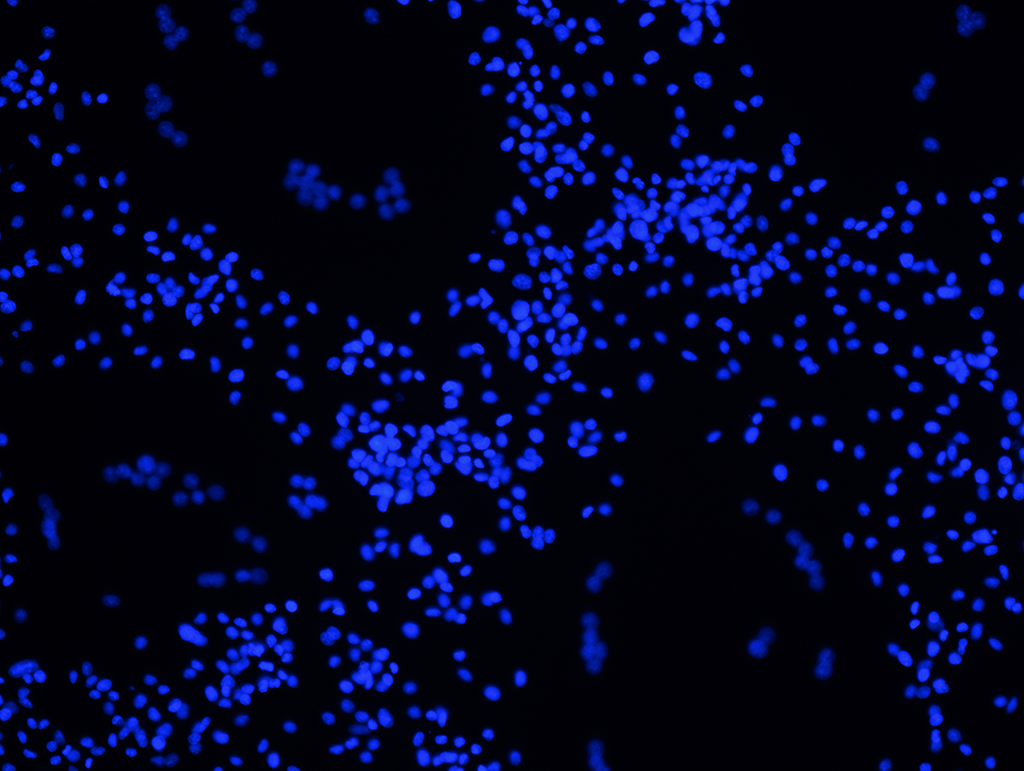

Supplement: Supplementary file 9 [file DataSheet7.ZIP › origin-F-actin(cell)/ECH-0.2 μM-DAPI.tif]

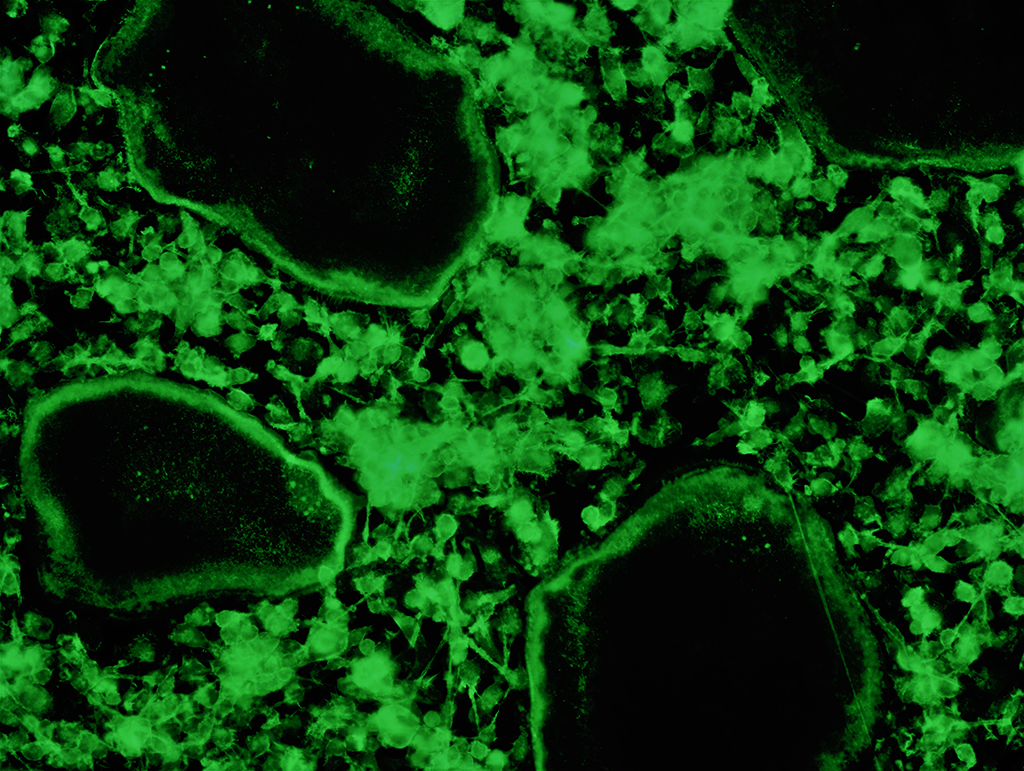

Supplement: Supplementary file 9 [file DataSheet7.ZIP › origin-F-actin(cell)/ECH-0.2 μM-F-actin.tif]

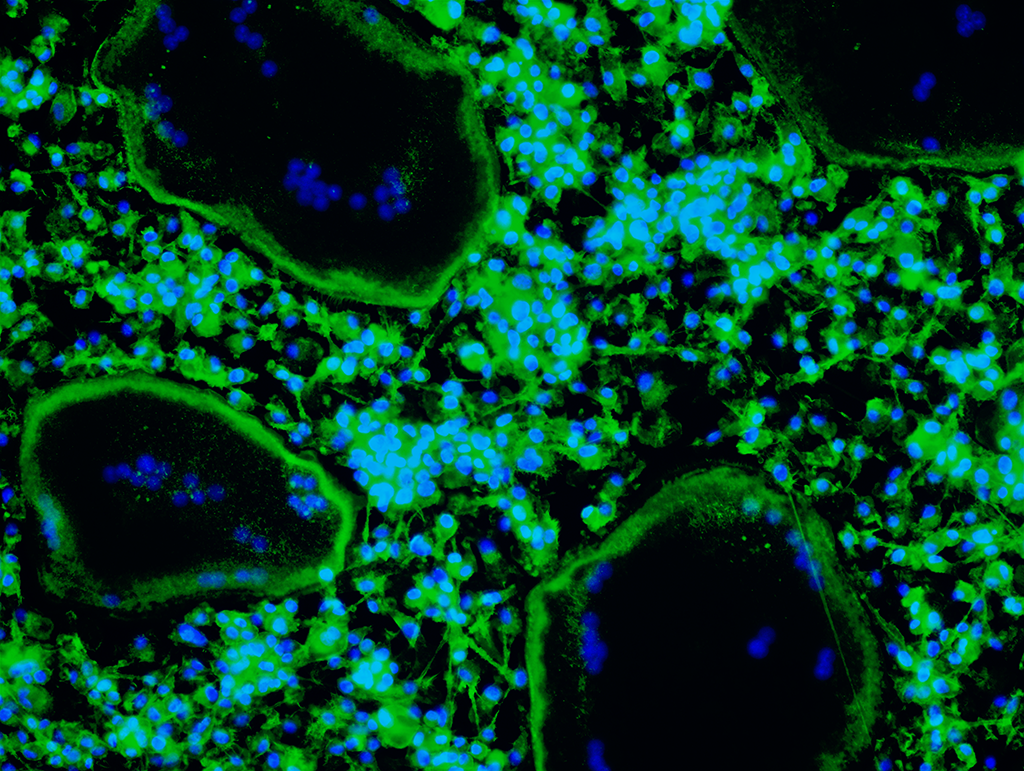

Supplement: Supplementary file 9 [file DataSheet7.ZIP › origin-F-actin(cell)/ECH-0.2 μM-merge.tif]

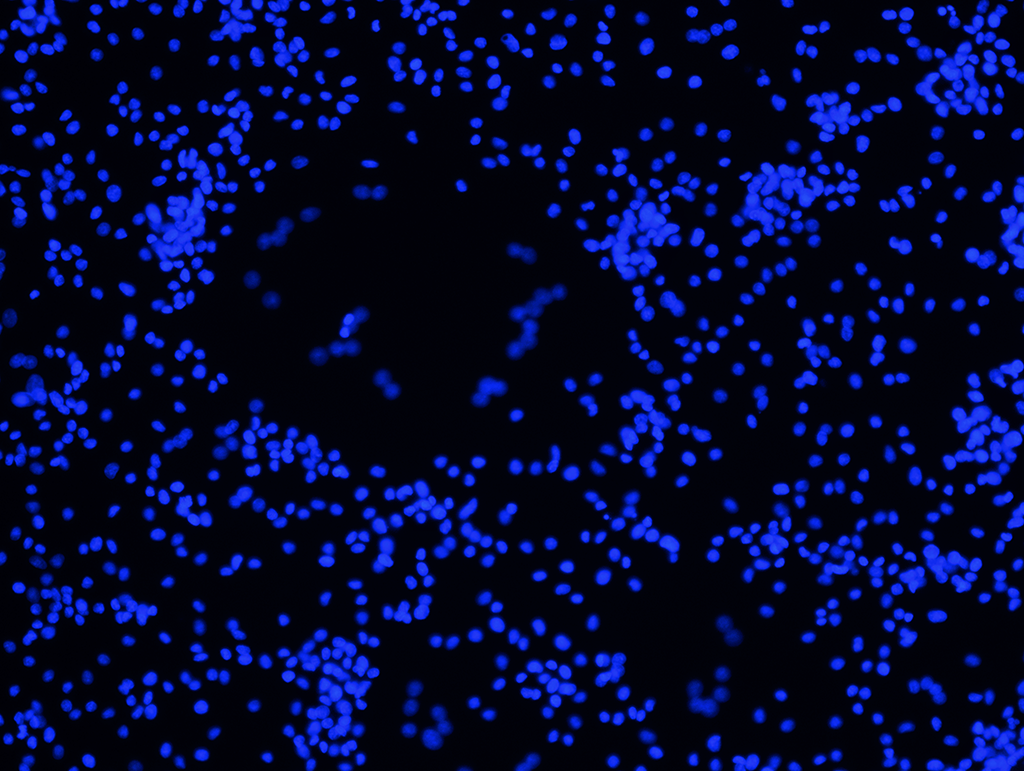

Supplement: Supplementary file 9 [file DataSheet7.ZIP › origin-F-actin(cell)/ECH-1 μM-DAPI.tif]

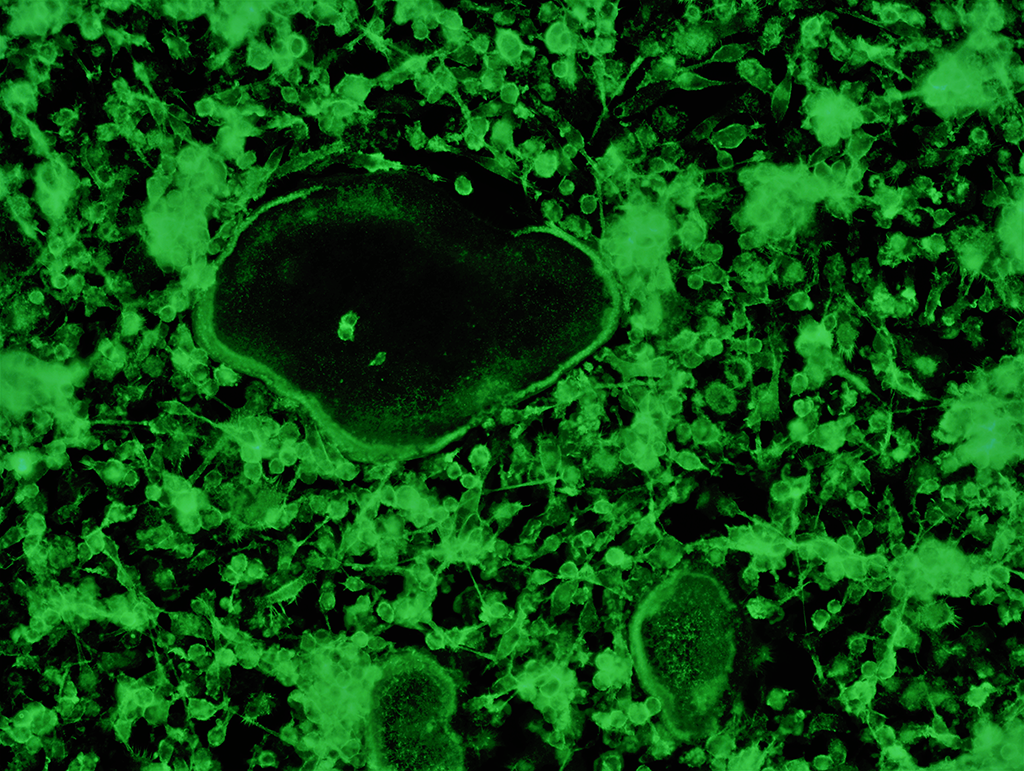

Supplement: Supplementary file 9 [file DataSheet7.ZIP › origin-F-actin(cell)/ECH-1 μM-F-actin.tif]

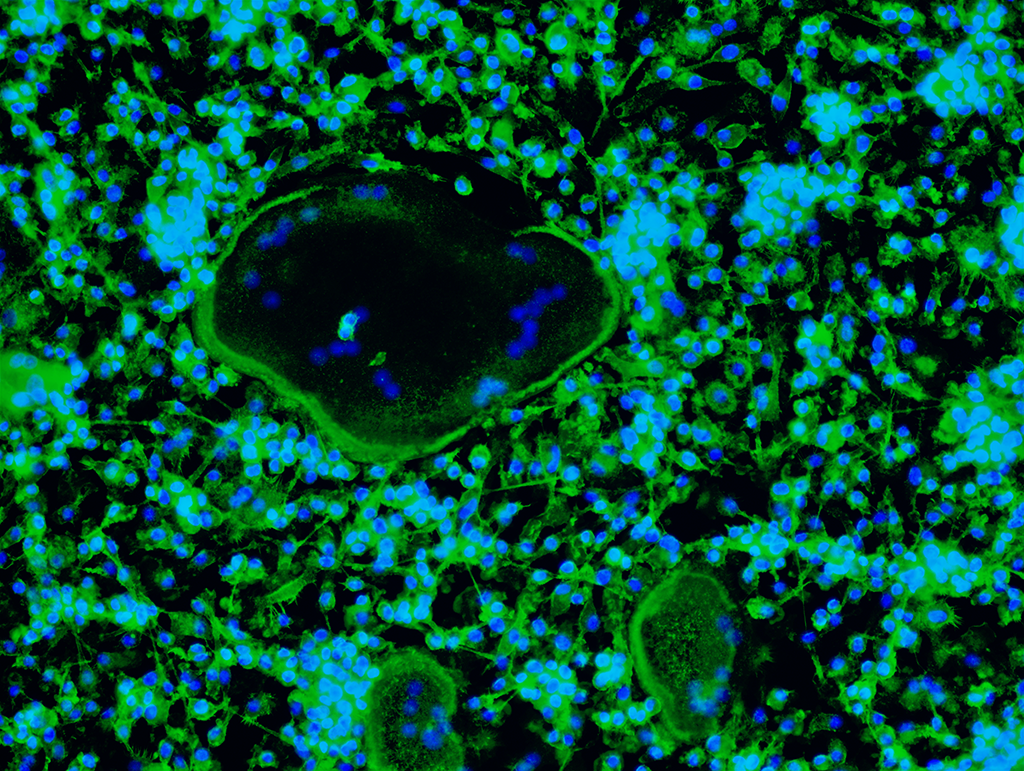

Supplement: Supplementary file 9 [file DataSheet7.ZIP › origin-F-actin(cell)/ECH-1 μM-merge.tif]

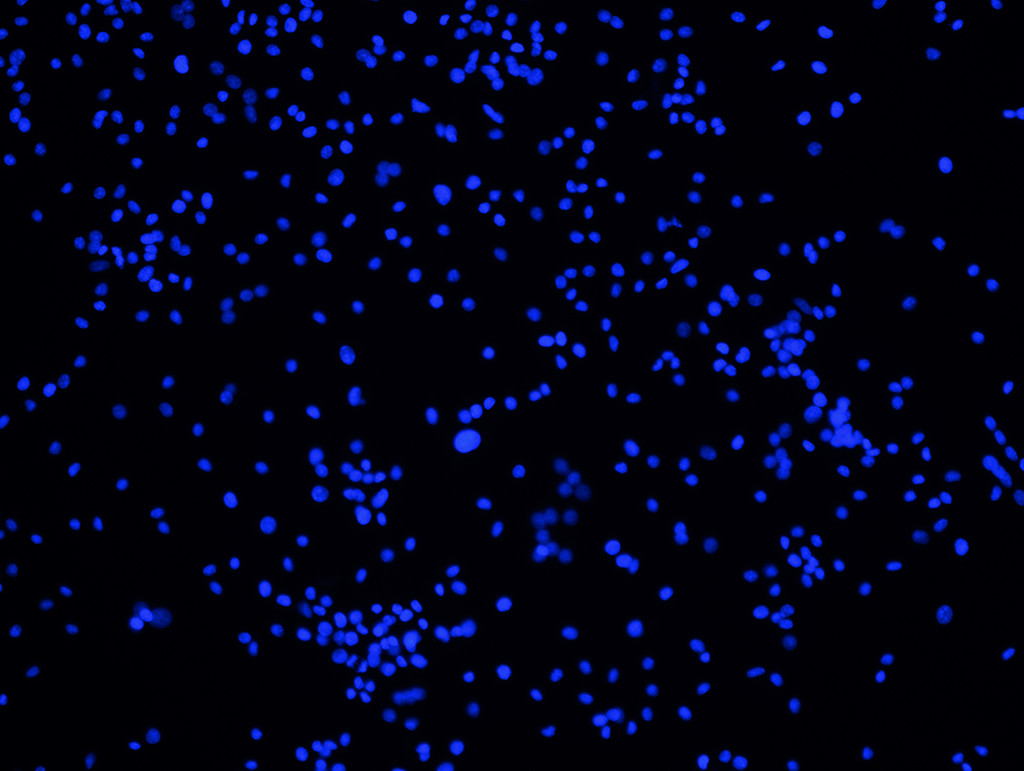

Supplement: Supplementary file 9 [file DataSheet7.ZIP › origin-F-actin(cell)/ECH-5 μM-DAPI.tif]

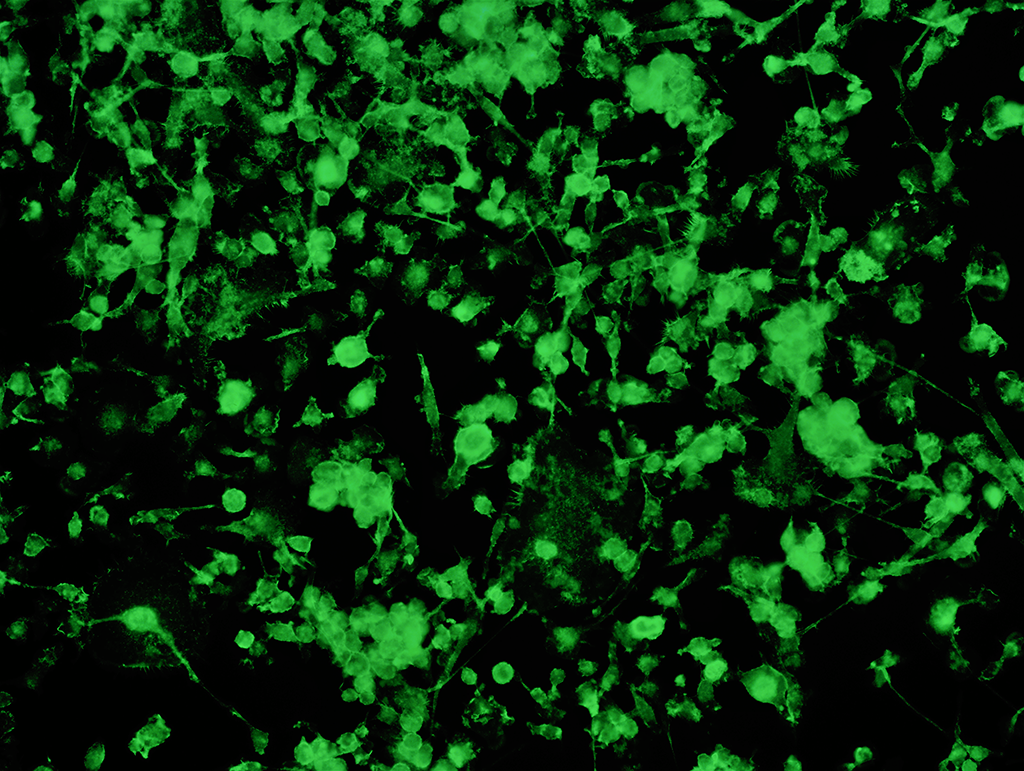

Supplement: Supplementary file 9 [file DataSheet7.ZIP › origin-F-actin(cell)/ECH-5 μM-F-actin.tif]

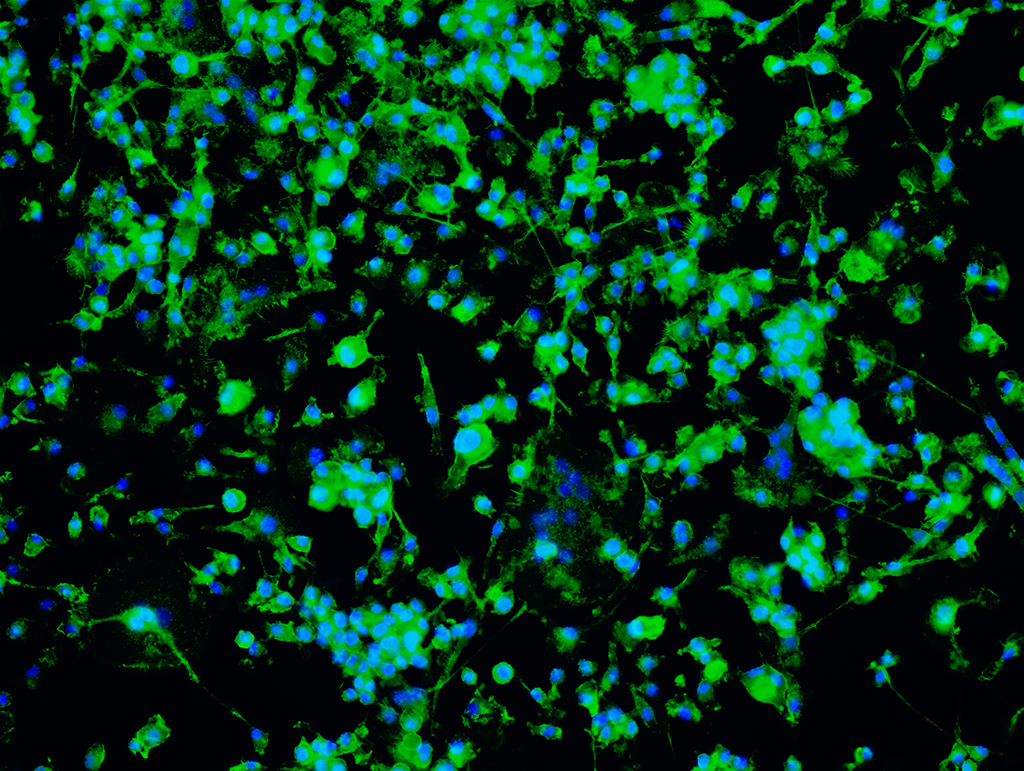

Supplement: Supplementary file 9 [file DataSheet7.ZIP › origin-F-actin(cell)/ECH-5 μM-merge.tif]
