# Supplementary material for: Echinacoside Inhibits Osteoclast Function by Down-Regulating PI3K/Akt/C-Fos to Alleviate Osteolysis Caused by Periprosthetic Joint Infection
Source: Front Pharmacol. 2022 Jun 24;13:930053. doi: 10.3389/fphar.2022.930053 (PMC9263215; doi:10.3389/fphar.2022.930053)
Supplement: Supplementary file 7 [file DataSheet8.pdf]

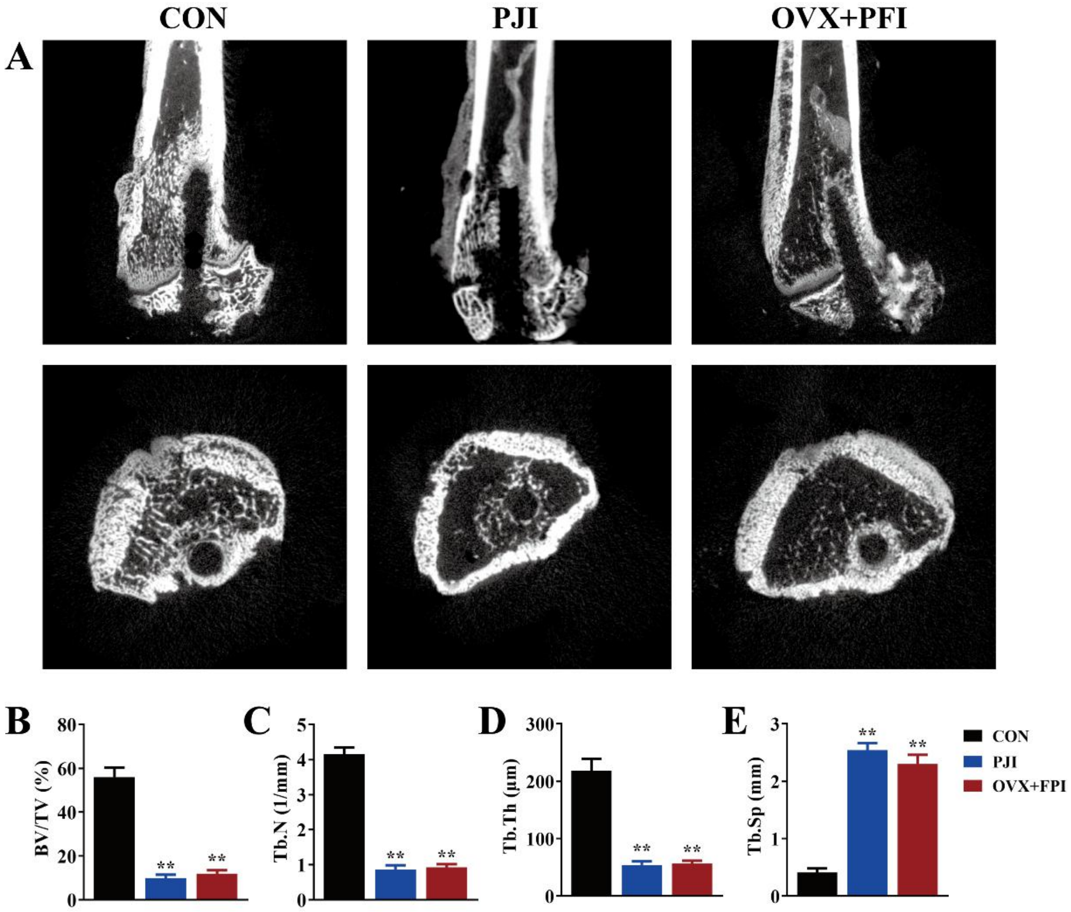

Supplementary Fig. S1 Bone mass between the PJI rat group and ovariectomy (OVX) + femur prosthesis implantation (FPI) rat group. (A) Representative micro-CT images in each treatment group of rats. (B-E) Quantification analyses of bone mass parameters, including BV/TV, Tb.N, Tb.Th, Tb.Sp. n = 8. **P<0.01(Compared with the control group). One-way ANOVA followed by Tukey's post hoc test was performed for multiple group comparisons. Control group (femur prosthesis implantation (FPI), surgery), PJI group (FPI surgery with infection), OVX + FPI group (ovariectomy + FPI surgery). BV/TV: bone volume per tissue volume; Tb.N: trabecula number; Tb.Th: trabecular thickness; Tb.Sp: trabecula separation.

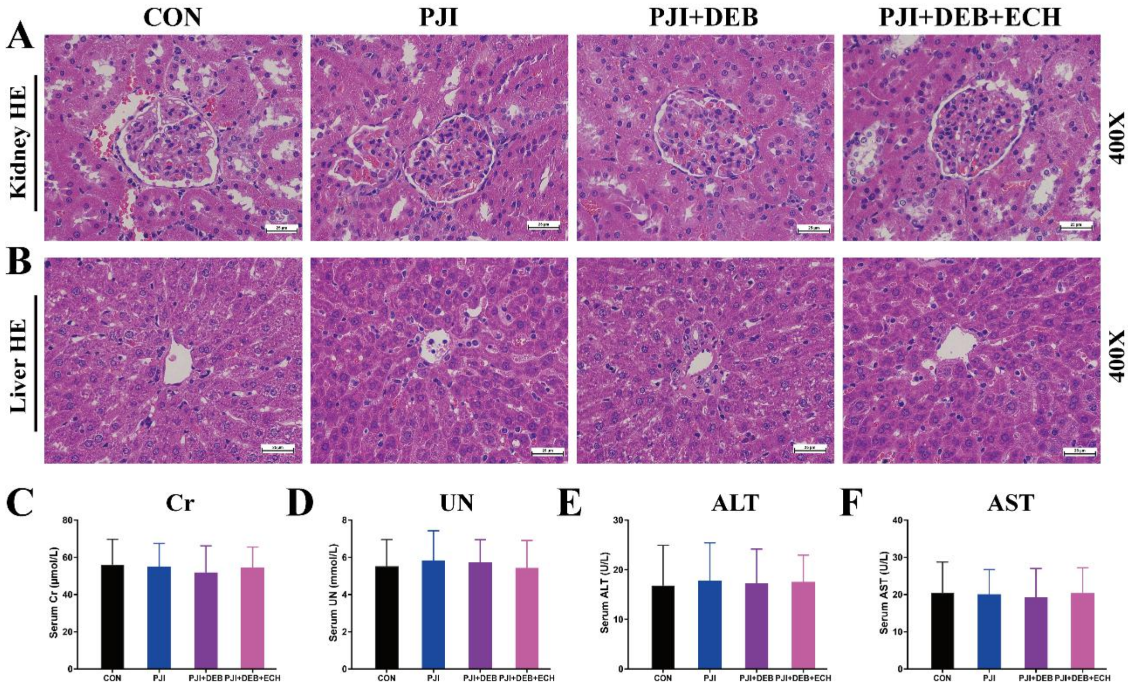

Supplementary Fig. S2 Safety evaluation of Echinacoside (ECH) treatment after debridement (DEB) of periprosthetic joint infection (PJI) in each rats group. (A) Representative pathological HE staining of the kidney (×400) at 4 weeks after debridement in each treatment group. (B) Representative pathological HE staining of the liver (×400) at 4 weeks after debridement in each treatment group. (C) Serum creatinine (Cr) at 4 weeks after debridement in each treatment group. (D) Serum urea nitrogen (UN) at 4 weeks after debridement in each treatment group. (E) Serum alanine aminotransferase (ALT) at 4 weeks after debridement in each treatment group. (F) Serum aspartate aminotransferase (AST) at 4 weeks after debridement in each treatment group. One-way ANOVA followed by Tukey's post hoc test was performed for multiple group comparisons. n = 10 per group.
